# Cationic Cyclodextrin-Based Carriers for Drug and Nucleic Acid Delivery

**DOI:** 10.3390/pharmaceutics17010081

**Published:** 2025-01-09

**Authors:** Adila Nazli, Milo Malanga, Tamás Sohajda, Szabolcs Béni

**Affiliations:** 1 Department of Pharmacognosy, Semmelweis University, 1085 Budapest, Hungary; nazli.adila@phd.semmelweis.hu; 2 CarboHyde Zrt., Berlini u. 47-49, 1045 Budapest, Hungary; milomalanga@gmail.com (M.M.); sohtam@gmail.com (T.S.); 3 Integrative Health and Environmental Analysis Research Laboratory, Department of Analytical Chemistry, Institute of Chemistry, Eötvös Loránd University, 1117 Budapest, Hungary

**Keywords:** targeted drug release, transfection efficiency, inclusion complex, plasmid DNA, microRNA, small interfering RNA

## Abstract

Cyclodextrins can serve as carriers for various payloads, utilizing their capacity to form unique host–guest inclusion complexes within their cavity and their versatile surface functionalization. Recently, cationic cyclodextrins have gained considerable attention, as they can improve drug permeability across negatively charged cell membranes and efficiently condense negatively charged nucleic acid due to electrostatic interactions. This review focuses on state-of-the-art and recent advances in the construction of cationic cyclodextrin-based delivery systems. First, we identified different cationic moieties that are commonly employed in the design of cyclodextrins with enhanced complexation ability. Subsequently, a wide range of cationic cyclodextrin-based drug delivery systems were analyzed with emphasis on chemistry, drug release profiles, and therapeutic outcomes. The evaluation of the delivery platforms was also based on the four major types of drugs, such as anticancer, anti-inflammatory, antibacterial, and antidiabetic agents. The delivery systems for nucleic acids were also summarized while focusing on their condensation ability, transfection efficiency, and biocompatibility in comparison to commercially available vectors such as PEI 25 kDa and lipofectamine 2000. Furthermore, we highlighted the potential of cationic cyclodextrins in constructing multimodal delivery systems for the simultaneous encapsulation of both drugs and nucleic acids. Finally, the challenges and limitations associated with cationic cyclodextrin setups were discussed.

## 1. Introduction

Cyclodextrins are cyclic oligosaccharides capable of entrapping the guest molecules within their hydrophobic cavity through non-covalent interactions. Cyclodextrin encapsulation of a guest molecule can improve its physicochemical properties and prevent its degradation in aqueous medium [1,2]. However, unmodified β-cyclodextrin is associated with nephrotoxicity and poor aqueous solubility, which limit its applications as a carrier for the delivery of therapeutic agents [3,4,5]. To overcome these drawbacks, various cyclodextrin derivatives have been developed by exploiting the numerous hydroxyl groups present on the cyclodextrin surface [2,6]. Charged cyclodextrins possess stronger complexation and solubilization abilities in comparison to other cyclodextrin derivatives. The ionic interactions on their surface and hydrophobic interactions inside the cavities could both contribute to improved drug solubilization [7]. Among charged cyclodextrins, cationic amphiphilic cyclodextrin derivatives have gained considerable attention as vectors for drugs and nucleic acid delivery in particular.

Cationic cyclodextrins interact with negatively charged nucleic acids through electrostatic interactions, which promote their condensation into stable structures termed polyplexes [8,9,10]. Moreover, the presence of these polyplexes, particularly the amine groups capable of protonation within their supramolecular structures, promotes the accumulation of protons and counter-ions inside the endosome (proton sponge effect). This leads to a rapid increase in acidity (lower pH) and causes an influx of water to balance the osmotic gradient, eventually resulting in endosomal rupture. This allows for the polyplexes to release their genetic payload into the cell’s cytoplasm, where it can exert its intended biological effect [11]. On the other hand, cell membranes contain negatively charged components (sialic acid and phospholipids), which can strongly interact with cationic drug carriers [12]. Hence, in drug delivery applications, cationic cyclodextrins can promote strong cellular interactions, improve drug permeability across biological membranes, and enhance cell uptake, resulting in increased therapeutic effectiveness [1,13]. However, the high positive charge of these systems can also pose significant challenges, particularly in terms of cell toxicity. Strong electrostatic interactions with cell membranes can lead to membrane destabilization, causing cell damage or death [14]. This cytotoxicity can limit the biocompatibility of cationic cyclodextrins and affect their overall safety in therapeutic applications. Therefore, achieving an optimal balance between effective drug delivery and minimizing toxicity is crucial. This balance presents a significant challenge, as enhancing delivery often involves increasing positive charges, which can simultaneously increase toxicity. Researchers must meticulously design and optimize these delivery systems to strike a balance between maximizing therapeutic benefits and minimizing adverse effects. This ongoing challenge underscores the need for continuous development and refinement of drug delivery technologies. This review aims to summarize recent advancements in the development of cationic cyclodextrin-based systems for delivering drugs and nucleic acids, highlighting progress and identifying areas for further research.

## 2. Cyclodextrin: Types and Functionalization with Cationic Moieties

**Scheme 1:**
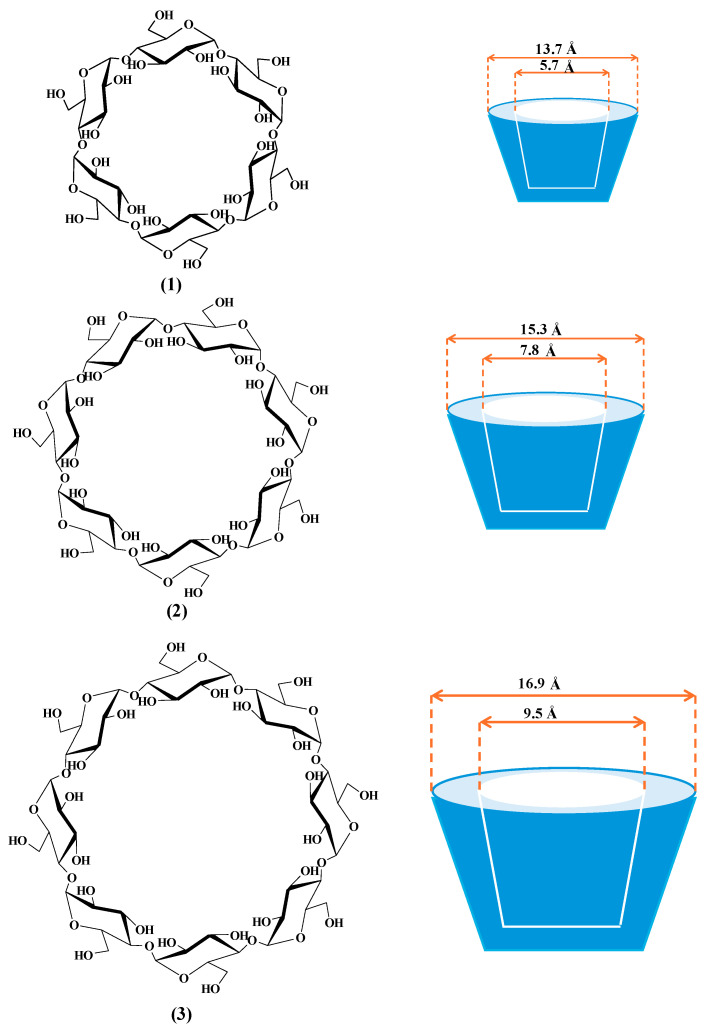
Chemical structures of (**1**) α-cyclodextrin, (**2**) β-cyclodextrin, and (**3**) γ-cyclodextrin, with illustrations showing their relative dimensions.

There are three types of natural cyclodextrins, including alpha-cyclodextrin (α-CD), beta-cyclodextrin (β-CD), and gamma-cyclodextrin (γ-CD), which consist of six, seven, and eight glucopyranose units, respectively (Scheme 1) [15,16].

Among these, α-CD contains a relatively smaller cavity for the entrapment of guest molecules. The cavity size of β-CD is large enough for the loading of most of the guest molecules, with efficient guest complexation and loading capacity. Moreover, β-CD is produced at a relatively low cost; hence, β-CD is the most commonly employed cyclodextrin for drug and nucleic acid delivery applications [17]. Furthermore, γ-CD contains an even larger cavity and is capable of entrapping larger-size guest molecules that could not be incorporated in α- and β-CDs [18]. However, γ-CD is more expensive than α-CD and β-CD due to its lower production efficiency. It requires more specialized and engineered enzymes for its synthesis, and the isolation process is more challenging, making it less commonly used in delivery applications [17,19].

**Figure 1:**
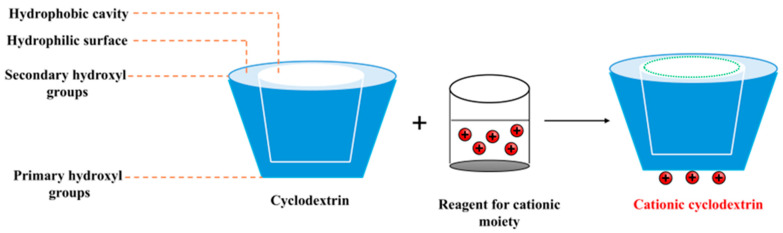
Pictorial depiction of cyclodextrin functionalization with cationic moiety.

When cyclodextrins are modified with cationic moieties, their potential as carriers based on electrostatic interactions is exponentially enhanced (Figure 1) [20,21,22].

CDs have been functionalized with a large variety of cationic moieties, including polyethylenimine (PEI), chitosan, poly(ε-lysine), poly (2-(dimethylamino) ethyl methacrylate) (PDMAEMA), and polyamidoamine (PAMAM). PEI is a commonly used polycation that contains a wide range of primary, secondary, and tertiary amines and imparts a high density of positive charge [23,24,25]. PEI is widely employed due to its defined size and molecular weight, high solubility in various solvents, and abundantly available amine groups for conjugation [26]. Chitosan is a cationic polymer containing an average amino group density of 0.837 per disaccharide unit [27]. The amino group at the C-2 position and hydroxyl group at C-6 position of chitosan are amenable to chemical modifications enabling the chitosan conjugation with a wide range of substances [28].

Poly(ε-lysine) is a homopolyamide composed of L-lysine residues that has been employed as a cationic agent for cyclodextrin complexation [29]. PDMAEMA serves as a cationic polyelectrolyte below pH 7.1 due to the protonation of its tertiary amine group [30]. PAMAM is another polycation containing numerous amine groups [31,32]. Moreover, cyclodextrins have also been coupled with cationic surfactants such as [hexadecyl(2-hydroxyethyl)dimethylammonium dihydrogen phosphate, HHDDP] [18], triethanolamine [33], benzalkonium chloride [34], and gemini-type surfactants [35].

Cyclodextrins are also allowed to react with choline chloride (CC), which acts as a precursor of quaternary ammonium groups [36]. Quaternary ammonium groups possess permanent positive charges and robust stability in the environment [20]. Furthermore, the quaternization of cyclodextrins enhances their aqueous solubility and reduces hemolytic as well as renal toxicities associated with parent cyclodextrins [37]. Guanidine has also been employed for the complexation of cyclodextrins, which contain an amidine functional group (-C(NH)-NH_2_) [20]. Moreover, imidazole containing a tertiary amine may serve as a cationic agent for cyclodextrins [13].

## 3. Cationic Cyclodextrins for Small-Molecule Drug Delivery

**Figure 2:**
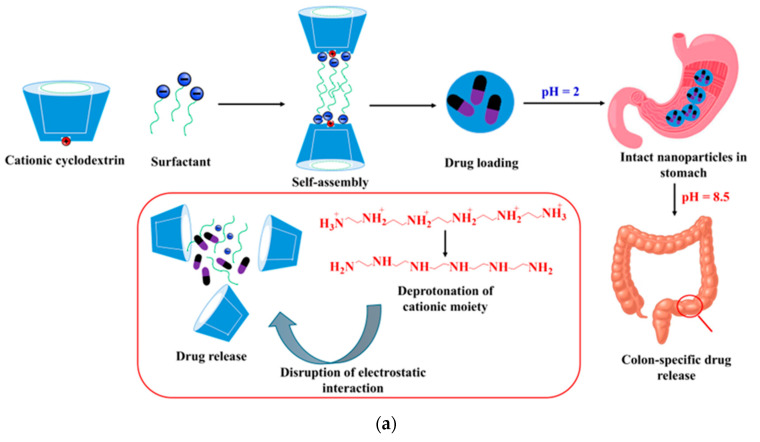

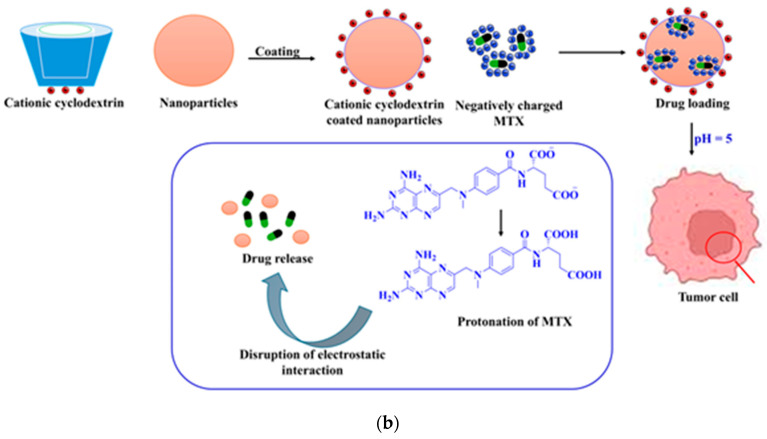
Cationic cyclodextrin-based systems release drugs either under (**a**) a basic environment or (**b**) an acidic environment.

A diverse array of small-molecule drugs has been successfully delivered using cationic cyclodextrins as carriers. This review categorizes these drugs into four main pharmacological groups, anticancer, anti-inflammatory, antibacterial, and antidiabetic agents, while also highlighting examples from other therapeutic classes. These cationic cyclodextrin-based delivery systems facilitate site-specific drug release under varying pH conditions (Figure 2) [13,38].

### 3.1. Anticancer Drugs

**Scheme 2:**
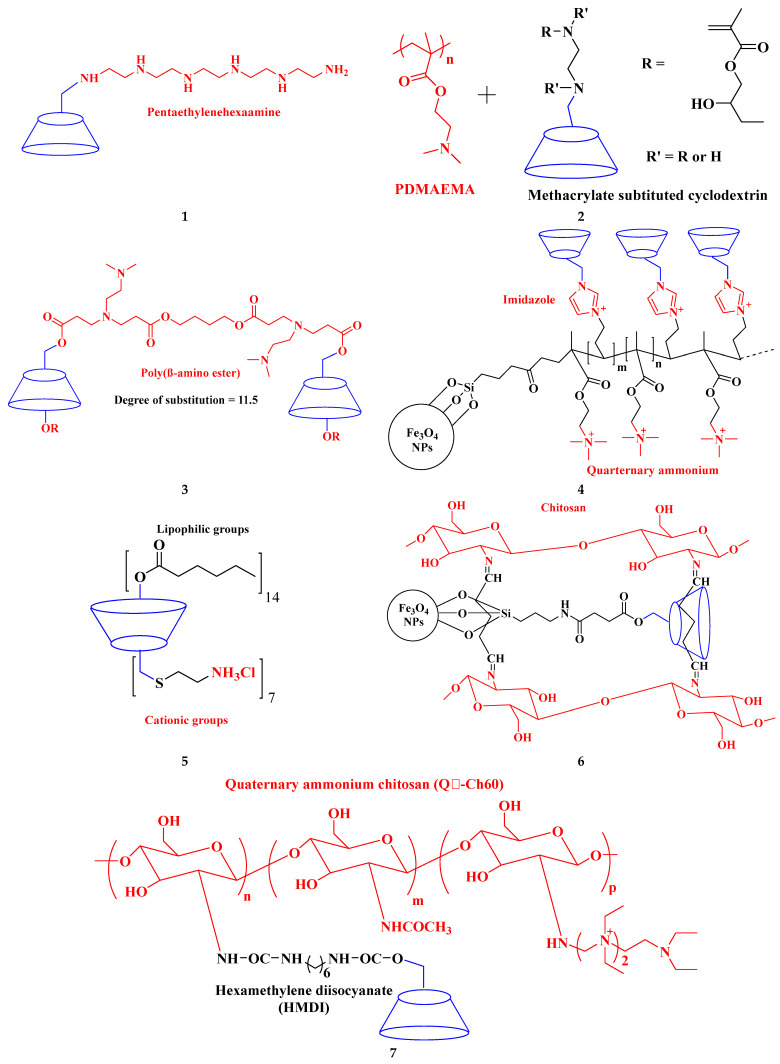

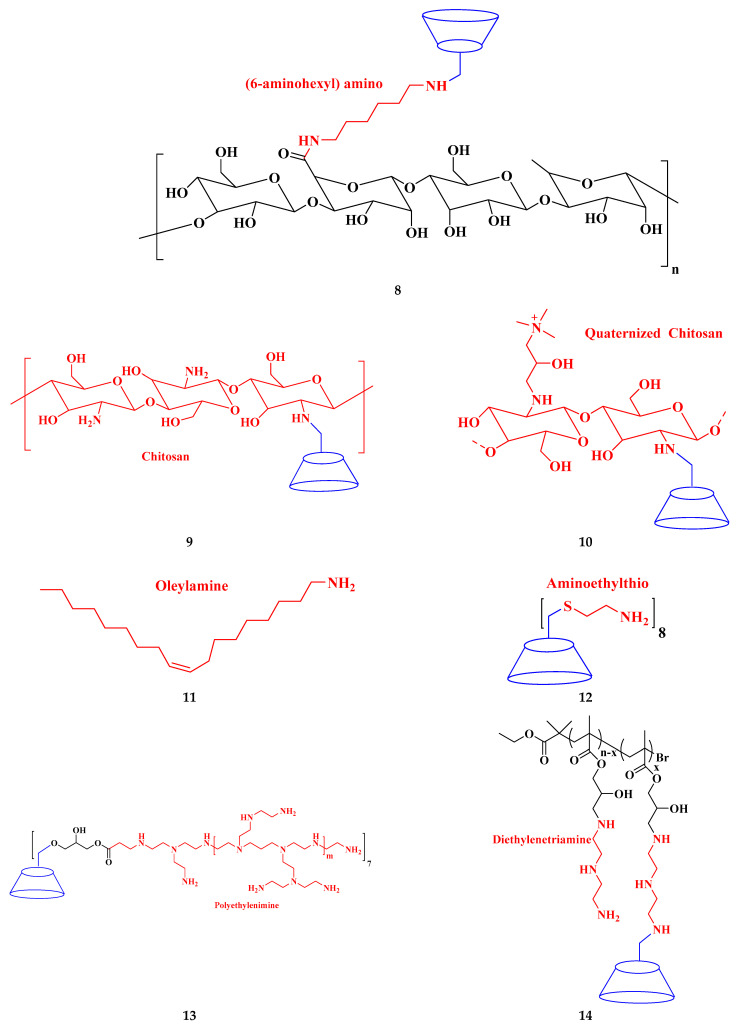
Chemical structures of cationic cyclodextrin-based systems employed for drug delivery.

Mono-(6-pentaethylenehexaamine)-β-cyclodextrin (PEHA-β-CD) is a polyamine derivative of cyclodextrin that is characterized by six positively charged amine groups under a neutral and acidic environment (Scheme 2 (1)). Li et al. synthesized nanoparticles (NPs) by establishing the electrostatic interaction between cationic PEHA-β-CD and an anionic surfactant, i.e., sodium dodecylbenzene sulfonate, that was loaded with **doxorubicin** (DOX) and celastrol (CLSL). The NPs were stable under acidic conditions while gradually disassembling with an increasing pH range from 2 to 10. It was suggested that the polyamine group of PEHA-β-CD undergoes deprotonation around and above its p*K*_a_ (6.3–7.0), which disrupts the electrostatic interaction leading to the disassembly of NPs (Figure 2a). The NPs liberated 7%, 14.8%, and 46.4% of DOX at pH of 1.2, 7.4, and 8.5, respectively. It indicates that PEHA-β-CD NPs may be a potential candidate for oral anticancer therapy with target specificity, as they can bypass the acidic environment of the stomach and selectively release the payload under alkaline conditions of the small intestine. The DOX(CSL)-loaded PEHA-β-CD NPs efficiently mediated the apoptosis of colon cancer cells (SW480) and liver cancer cells (SMMC-7721). Moreover, NPs showed lower toxicity to normal epithelial cells as compared to free DOX [38].

Zhang et al. fabricated a DOX-entrapped star polymer containing PDMAEMA and methacrylate-substituted β-cyclodextrin (Scheme 2 (2)) as a core along with a poly(ethylene glycol) (PEG) chain. The release rate of DOX was higher at pH 5 than that at pH 7.4, hence confirming the pH-responsive potential of star polymer. It was suggested that the core is slowly ruptured in acidic environment due to the repulsion of protonated PDMAEMA, which promotes the release of payload [39]. The DOX-loaded star polymers inhibited tumor growth at a higher rate than free DOX in xenograft mice bearing human cervical cell lines. The high anti-tumor activity of the star polymer system was attributed to its high cellular uptake followed by penetration into tumor tissues through an enhanced permeability and retention (EPR) effect and the pH-responsive release of DOX [30].

Gil et al. prepared CD-p-AE NPs by coupling β-cyclodextrin with poly(β-amino ester) (Scheme 2 (3)), which were entrapped with DOX. The permeability of NPs was investigated across the blood–brain barrier by using monolayers of bovine brain microvascular endothelial cells and human brain microvascular endothelial cells. The NPs showed 100% and 60% higher permeability than that of the dextran control across and the constructed monolayers [40]. It has been reported that cationic charges of NPs can enhance their permeability across the BBB due to adsorptive-mediated endocytosis [41]. The presence of tertiary amine groups might be attributed to the high permeability of NPs that are positively charged under physiological conditions. Moreover, NPs liberated DOX in a sustained manner for up to 1 month. The hydrophobic interaction between DOX and CD-p-AE NPs might be strong enough to restrict the dissociation of DOX from the NPs, leading to a sustained DOX release [40].

Ahmadi et al. prepared Fe_3_O_4_ NPs that were coated with imidazole and quaternary ammonium-grafted β-cyclodextrin (Scheme 2 (4)). **Methotrexate** (MTX) was efficiently entrapped inside the NPs through electrostatic interactions, as carboxylic acid groups of MTX exist in an anionic form at pH 7.4. The MTX release profile was investigated under simulated physiological (pH 7.4) and tumor (pH 5) environments. MTX was more efficiently released (73%) at pH 5 than at pH 7.4 (44%) within 300 h. The carboxylates of MTX are partially protonated under acidic conditions, which disrupt the electrostatic interactions with positively charged NPs and promote drug release (Figure 2b). As MTX is not completely protonated at pH 5, it still interacts with NPs, which might explain its incomplete drug release. MTX-loaded NPs showed relatively higher cytotoxicity than free MTX towards Saos-2 bone cancer cells. Moreover, flow cytometry cell analysis revealed that 9.21% of the MTX-loaded NPs were taken up by the Saos-2 cells during 1 h [13].

Varan et al. compared the potential of non-ionic (6OCapro βCD) and cationic (PC βCDC6) β-cyclodextrin derivatives for **paclitaxel** (PTX) delivery. PC βCDC6 possesses 7 cationic groups on the primary face and 14 lipophilic groups on the secondary face (Scheme 2 (5)) while 6OCapro βCD contains 7 lipophilic groups on the primary face. The anti-tumor potential of PTX-loaded NPs was investigated against MCF-7 cell lines. 6OCapro βCD- and PC βCDC6-loaded NPs showed 51.7% and 30.7% cell viability, respectively. The electrostatic interaction of positively charged NPs with cell membranes was attributed to the higher anti-tumor activity of PC βCDC6-loaded NPs [12].

These delivery systems have shown significant potential in enhancing the targeted release of anticancer drugs, with pH-responsive properties enabling controlled drug release in tumor environments, a characteristic common to other cationic β-cyclodextrin-based systems, as summarized in Table 1 (Table S1). Additionally, these systems improve drug permeability, as evidenced by enhanced blood–brain barrier penetration and sustained drug release, resulting in higher anti-tumor activity compared to free drugs. Overall, cationic β-cyclodextrin-mediated drug delivery holds great promise for improving the efficacy and specificity of anticancer treatments.

### 3.2. Anti-Inflammatory Drugs

The class of nonsteroidal anti-inflammatory drugs (NSAIDs) typically contains carboxylic acid or similar acidic functional groups that play a key role in their pharmacological activity and pharmacokinetics. However, these drugs often exhibit limited aqueous solubility. Combining cyclodextrins with positively charged functional groups in their delivery systems can provide significant advantages over using the drug alone. In contrast, glucocorticoid steroids, another class of anti-inflammatory drugs, are highly lipophilic. Utilizing cationic cyclodextrins as carriers for these compounds can enhance bioavailability and improve mucoadhesion, offering a more effective delivery approach.

**Indomethacin** (IDM) is an arylacetic acid drug that possesses poor aqueous solubility [7]. Anirudhan et al. fabricated a chitosan-coupled β-cyclodextrin-grafted silylated magnetic NP-based hydrogel (CTSCD-g-SilylMNP) (Scheme 2 (6)) that was loaded with IDM. The hydrogel attained 90% swelling at pH 1.4 and 60% swelling at pH 7.4, within 6 h. Consequently, a higher drug release rate was noticed at pH 1.4 than at pH 7.4. The pH-dependent behavior was attributed to the protonation of the basic sites of the hydrogel, which induced electrostatic repulsion between the hydrogel and IDM, resulting in its swelling and subsequent release of the payload. Hence, this cationic cyclodextrin-based formulation can be employed for targeted and controlled drug delivery [47].

**Dexamethasone** (DXM) is a lipophilic glucocorticoid steroid that depicts limited ocular bioavailability when instilled as eye drops.

Piras et al. connected methyl-β-cyclodextrin (MCD) with quaternary ammonium chitosan (QA-Ch60) through hexamethylene diisocyanate (HMDI) as a spacer (QA-Ch-MCD) (Scheme 2 (7)). The conjugates formed a stable complex with DXM while improving its aqueous solubility and retained significant mucoadhesion. Moreover, conjugate-treated RCE cell lines showed high viability, hence confirming their biocompatibility [48].

Choi et al. fabricated a 6-(6-aminohexyl)-amino-6-deoxy-β-cyclodextrin-gellan gum complex hydrogel (HCD-GG) (Scheme 2 (8)) that was loaded with DXM (Dx@HCD-GG). The DXM release rate was proportional to the cyclodextrin content incorporated in the hydrogel. The hydrogel was found to be biocompatible. The cell-cultured Dx@HCD-GG hydrogel showed the highest glycosaminoglycan (GAGs) and double-stranded DNA (dsDNA) contents, indicating the enhanced chondroprotective effect of DXM. During the in vivo study (cartilage defect model), the hydrogel-treated group showed highly dense regenerated tissues that interacted well with the surrounding tissues. Moreover, the formation of a glycosaminoglycan matrix was also noticed, hence confirming the cartilage regeneration potential of Dx@HCD-GG hydrogel [49]. 

**Ketoprofen** (KTP), a member of the arylpropionic acid group of NSAIDs, is characterized by poor aqueous solubility, limited bioavailability, and a short plasma half-life, necessitating frequent dosing. To address these challenges, Yuan et al. synthesized chitosan-grafted β-cyclodextrin nanoparticles (CD-g-CS) (Scheme 2 (9)) for KTP encapsulation. Unlike bare chitosan nanoparticles (NPs), which exhibited significant swelling in phosphate-buffered saline (PBS) within 48 h, CD-g-CS NPs remained stable for up to 7 days. This enhanced stability is likely due to the more compact core structure of CD-g-CS NPs, attributed to weak intermolecular interactions between cyclodextrin and chitosan. The drug release profile of CD-g-CS NPs was evaluated at pH 4.0 and 6.8, revealing an initial rapid release phase (0.5 h) followed by sustained release over 12 h at both pH levels. The initial burst release was attributed to the desorption of surface-bound or adsorbed drug, while the sustained release was driven by drug diffusion through the nanoparticle matrix and subsequent matrix erosion. Interestingly, the release rate of KTP from CD-g-CS NPs was higher in mildly acidic conditions compared to bare chitosan NPs. This phenomenon may result from reduced hydrogen bonding and fewer available amine groups in chitosan due to cyclodextrin coupling, which weakens the interaction between chitosan and KTP. At pH 6.8, CD-g-CS NPs demonstrated a more sustained release profile compared to bare chitosan NPs. Increasing the degree of cyclodextrin substitution further slowed the drug release rate. This effect is attributed to the ability of cyclodextrin-modified NPs to entrap more KTP through a network of weak intermolecular forces, promoting prolonged release. These findings highlight the potential of CD-g-CS NPs as a promising delivery system for improving the therapeutic profile of KTP [27].

**Diclofenac sodium** (DCF) faces challenges in transdermal delivery due to its low partition coefficient. Yan et al. developed hydroxypropyl–β-cyclodextrin-grafted polyethylenimine (HP-β-CD-PEI1800) as a penetration enhancer to address this issue. At equivalent doses, HP-β-CD-PEI1800 significantly improved DCF skin penetration, achieving up to 15 times higher penetration compared to the negative control. Additionally, HP-β-CD-PEI1800 demonstrated biocompatibility with minimal cytotoxicity and no signs of skin irritation [50].

**Meloxicam** (MLX) is another poor water-soluble drug whose ternary complex was coupled with ß-cyclodextrin and triethanolamine (MLX-β-CD-TEA). The resultant MLX-β-CD-TEA conjugate showed significantly improved dissolution with ~85% cumulative drug dissolved compared to pure MLX (~30%). Moreover, MLX-β-CD-TEA ternary complexes exhibited prolonged and almost complete drug release in an acidic environment. Followed by oral administration, MLX-β-CD-TEA ternary complexes showed higher edema inhibition as compared to pure MLX. The improved anti-inflammatory potential was attributed to the ameliorated solubility and dissolution of MLX in gastric fluid [33].

**Budesonide** (BUD) has low oral bioavailability due to poor solubility and hepatic first-pass metabolism. Pattanaik et al. enhanced its mucosal permeation by formulating a hydrogel film incorporating benzalkonium chloride (a quaternary surfactant) and β-cyclodextrin. This formulation significantly improved dissolution and mucosal permeation compared to a film without cyclodextrin and benzalkonium. Topical application to rabbit eyes reduced inflammation symptoms, such as redness, within 3 h [34]. Additional studies on cationic β-cyclodextrin-mediated anti-inflammatory drug delivery are summarized in Table 2 (Table S2).

### 3.3. Antibacterial Drugs

The development of cationic cyclodextrin-based drug delivery systems to enhance the antibacterial efficacy of antibiotics is driven by several key factors. The cyclodextrin structure improves drug solubility, stability, and release control, while the cationic charge facilitates bacterial targeting, membrane disruption, and biofilm penetration. Together, these features enable more effective, sustained, and targeted antibacterial therapy, which is crucial in combating the growing issue of antibiotic resistance.

**Ciprofloxacin** (CFX) is a broad-spectrum antibiotic that possesses a short half-life, poor water solubility, and is susceptible to lysosomal degradation, thus requiring a sustained drug delivery system. Dhiman et al. fabricated quaternized cyclodextrin-grafted chitosan (QCD-g-CH) NPs (Scheme 2 (10)) that were loaded with CFX. The nanoformulation initially released the CFX at a higher rate (~35% within 0.5–1 h) due to the desorption of the surface-adsorbed drug. Subsequently, CFX was released in a sustained manner (~90% within 24 h) that was mediated by CFX diffusion through the NPs matrix and erosion of the polymeric matrix. Moreover, the nanoformulation showed highly potent antibacterial activity for both *Staphylococcus aureus* and *Escherichia coli*. It was suggested that positively charged quaternized chitosan is efficiently adsorbed on negatively charged bacterial surfaces and promotes the leakage of proteinaceous and other intracellular constituents [55]. Keshavarz et al. reported the in situ fabrication of CFX-loaded polyamidoamine/β-cyclodextrin (β-CD)/silver NPs on polyester fabric. The resultant fabric showed higher drug absorption and sustained drug release as compared to raw fabric. Modified fabric showed 100% antibacterial efficiency for both *E. coli* and *S. aureus*. Moreover, the fabric did not impart any cytotoxic impact on fibroblast cells, hence confirming its biocompatibility [31].

Salih et al. developed a cationic amphiphile derivative (βCD-OLA) by the coupling of oleyl amine (Scheme 2 (11)) and β-cyclodextrin that was entrapped with **vancomycin** (VCM). βCD-OLA released the VCM in a sustained manner, i.e., ~65% and ~80% after 24 h and 48 h, respectively. Furthermore, BCD-OLA/VCM showed a fourfold reduced MIC towards methicillin-resistant *S. aureus* as compared to free vancomycin. BCD-OLA/VCM caused the 459-fold reduction in intracellular bacteria using infected human embryonic kidney cells (HEK) and an 8-fold reduction in infected macrophages as compared to free vancomycin. βCD-OLA was considered biocompatible based on high viability observed in various cell lines [56].

Evangelista et al. developed supramolecular polyelectrolyte complexes (SPEC) by exploiting the electrostatic interactions between a positively charged cyclodextrin-grafted chitosan derivative and negatively charged carrageenan for the encapsulation of **silver sulfadiazine** (SSD). The SSD/SPEC complexes exhibited smaller zones of inhibition compared to free SSD for *S. aureus*, *Klebsiella pneumoniae*, and *E. coli*, respectively. The enhanced antibacterial activity was likely due to the electrostatic interactions between the positively charged amine groups of chitosan and the negatively charged components of the bacterial cell wall. These interactions increase the permeability of the antibiotic across the bacterial membrane, leading to the leakage of intracellular components (such as nucleic acids and proteins) and ultimately causing bacterial cell death [57].

The β-lactamase-mediated degradation of the β-lactam ring is the most common mechanism of resistance to penicillins. Agnes et al. prepared a positively charged octakis [6-(2-aminoethylthio)-6-deoxy]-γ-cyclodextrin derivative (γCys) (Scheme 2 (12)) that was complexed with **oxacillin** (OXA). The study demonstrated that γCys complexation resulted in a 2.3-fold reduction in β-lactamase-induced OXA hydrolysis. Additionally, γCys was efficiently internalized by macrophages, with 25% internalization occurring within the first 15 min and 99.8% within 24 h. Phagocytosis of invading pathogens is a key component of host immune defense, suggesting that the increased concentration of OXA within macrophages via Cys complexation may significantly enhance antibacterial effects. Furthermore, γCys was found to be biocompatible, as shown by the high viability of L929 cells. [58]. Thomsen et al. used octakis[6-(2-aminoethylthio)-6-deoxy]-γ-cyclodextrin for delivering **rifampicin** (RFP) to biofilms. The cyclodextrin tagged with fluorescein (FITC) was evenly distributed throughout live *S. epidermidis* biofilms, demonstrating that the cyclodextrin fully penetrates the biofilm. Moreover, it was primarily localized around the bacterial cells, rather than intracellularly, suggesting its distribution across the extracellular matrix of the biofilm. The bare cationic cyclodextrin had a noticeable impact on biofilm viability, reducing it by 20–50%, indicating the inherent antibacterial potential of this derivative. The drug-loaded RFP/cyclodextrin complex reduced biofilm viability to near-background levels (~100%), while free RFP showed only moderate anti-biofilm activity (~60% reduction). The remarkable anti-biofilm potential of this combination may be attributed to the enhanced solubility of RFP upon complexation and/or synergistic interactions with the components of the biofilm [59].

Wei et al. developed metal–organic frameworks (MOFs) based on γ-cyclodextrin and potassium ions (γ-CD-MOF) that were loaded with **enrofloxacin** (ENF). The γ-CD-MOF/ENF complex showed lower minimum inhibitory concentrations for *E. coli* and *S. aureus* compared to free ENF. The γ-CD-MOF released 40% of ENF in 1 h and 87.5% in 4 h. The antibacterial activity of the released ENF was significantly higher, with γ-CD-MOF/ENF achieving nearly 100% bacterial inhibition within 6 min and maintaining around 95% inhibition for 24 min. In contrast, free ENF showed 85% inhibition at 6 min and 70% at 24 min. γ-CD-MOFs also demonstrated biocompatibility, with no cytotoxic effects observed on L929 cells [60]. Further studies based on cationic β-cyclodextrin-mediated antibacterial drug delivery are summarized in Table 3 (Table S3).

### 3.4. Antidiabetic Drugs

Developing cationic cyclodextrin-based systems for antidiabetic drug delivery is promising due to their ability to enhance drug stability, solubility, and controlled release. Cationic cyclodextrins can improve the bioavailability of antidiabetic drugs by protecting them from degradation in the gastrointestinal tract, promoting better absorption and ensuring sustained therapeutic effects. Their electrostatic interactions with the antidiabetics and biological membranes can also enhance targeted delivery, reduce side effects, and improve patient compliance by enabling oral or non-invasive administration routes. This makes cationic cyclodextrin-based systems an effective strategy for improving the efficacy of antidiabetic treatments.

**Metformin hydrochloride** (MTF) is a first-line antidiabetic drug primarily absorbed in the small intestine. However, it degrades easily in the gastrointestinal tract before reaching the target site, limiting its therapeutic efficacy. Teng et al. developed MTF-loaded supramolecular nanoparticles (NPs) by exploiting the electrostatic interactions between cationic mono-[3,3-(diaminodipropylamine)-6-deoxy]-β-cyclodextrin (DPN-β-CD) and anionic sodium dodecylbenzene sulfonate (SDBS). These NPs released significantly less MTF in simulated gastric fluid compared to the free drug solution. In contrast, the NPs released MTF at higher rates in more alkaline conditions, suggesting that DPN-β-CD/SDBS-based NPs could reduce MTF degradation in the stomach and enhance absorption in the small intestine [67].

Oral drug administration is preferred for patient compliance, but challenges like low oral bioavailability due to poor intestinal permeability and pre-systemic degradation remain. Presas et al. developed a nanoformulation based on cationic amphiphilic cyclodextrin (click propyl-amine cyclodextrin, PA-CD) for the intestinal delivery of **liraglutide** (LTD). This formulation prevented LTD degradation in simulated intestinal fluid (up to 4 h), while the LTD solution rapidly degraded (within 5 min). Following intestinal administration, LTD-NPs reduced glucose levels, closely matching the hypoglycemic effect of subcutaneous LTD solution. Thus, PA-CD-based NPs effectively deliver LTD through the gastrointestinal tract with comparable hypoglycemic potential to subcutaneous administration [68].

Pulmonary delivery is a non-invasive route for systemic drug administration, but some macromolecules still show lower bioavailability compared to intravenous delivery. Zhang et al. used hydroxypropyl-β-cyclodextrin-grafted polyethylenimine (HP-β-CD-PEI) (Scheme 2 (13)) to enhance the pulmonary absorption of **insulin**. Among different polymers, HP-β-CD-PEI1800 showed the highest absorption efficiency, followed by HP-β-CD-PEI10000 and HP-β-CD-PEI600. The positive charge of HP-β-CD-PEI promoted electrostatic interactions with the negatively charged mucus membrane, improving insulin absorption [69].

Wang et al. prepared cationic polymers by the functionalization of linear and star-shaped poly(glycidyl methacrylate)s with mono(6-(diethylenetriamine)-6-deoxy)-β-cyclodextrin (CD-DETA-PGOHMAs) (Scheme 2 (14)). Moreover, bare DETA-PGOHMAs were also prepared without involving cyclodextrins while both types of polymers were employed to form polyelectrolyte complexes (PECs) with insulin. The cumulative release of insulin from CD-series complexes (~80–95%) was higher than that of D-series complexes (~57–67%). The strong electrostatic attraction between amino PGOHMAs and insulin formed extremely compact PECs, hence limiting the insulin release. However, the inclusion interaction between CD-DETA-PGOHMAs and insulin is relatively weaker than electrostatic interaction, which could easily mediate the insulin release. Moreover, star-shaped polymers liberated the insulin at a lower rate (~80% in 6 h) than the linear polymers (~95% in 6 h), which might be due to their steric hindrance effect. The CD-series showed less toxicity towards L929 cells (nearly 100% viability) than the D-series (~10–70% viability); hence, the introduction of cyclodextrin mitigated the toxicity of amino PGOHMA by decreasing the density of amino groups [70].

Zhang et al. developed quaternary ammonium-containing cationic β-cyclodextrin polymers (CPβCDs) for insulin-loaded alginate/chitosan nanoformulations. Simple alginate/chitosan NPs released up to 60% insulin in simulated gastric fluid (pH 1.2), but only 18% in simulated intestinal fluid (pH 6.8), resulting in significant drug loss before reaching the target site. The optimized CPβCD-insulin-loaded NPs released 48% insulin in simulated gastric fluid and 40% in simulated intestinal fluid. The polymeric chain and positive charge of the CPβCDs helped retain insulin within the core of the alginate/chitosan NPs, reducing degradation in the stomach and improving its release in the intestine [71]. All examples discussed in this section ae compiled in Table S4.

### 3.5. Miscellaneous Drugs

**Vitamin B2**, an essential vitamin for human health, suffers from low water solubility, resulting in a poor pharmacokinetic profile. To address this, Hydari et al. developed a water-soluble cationic polymer, poly(β-CD-co-guanidine) (CAβCDP), using β-cyclodextrin (β-CD) and guanidine (GN) as building blocks, with epichlorohydrin (EP) as a crosslinker. Vitamin B2 was effectively incorporated into CAβCDP, resulting in a significant improvement in aqueous solubility. The solubility of the CAβCDP/vitamin B2 complex (673 g/L) was markedly higher than that of bare vitamin B2 (0.078 g/L). This enhancement was attributed to the formation of inclusion complexes between vitamin B2 and CAβCDPs. Additionally, CAβCDP-5 (β-CD/GN molar ratio 1:5) exhibited pH-dependent release profiles, with 98.5%, 81.5%, and 97.6% release at pH 10, 7.4, and 1.2, respectively, after 23 h. A more sustained release was observed with CAβCDP-15 (β-CD/GN molar ratio 1:15), which showed 89.1%, 71.5%, and 91.7% release under the same conditions. The slower release from CAβCDP-15 was attributed to stronger electrostatic interactions between the vitamin and the cationic polymer [72].

**Heparin**, a widely used anticoagulant, has poor oral bioavailability due to its high molecular weight and significant negative charge density, which limit its therapeutic potential. Its chemical structure contains sulfate groups, imparting negative charges across a wide pH range (3 to 11). Solatani et al. utilized a quaternary ammonium-containing cationic β-cyclodextrin polymer (CPβCD) to complex heparin through electrostatic interactions. A self-nano-emulsifying drug delivery system (SNEDDS) was developed, incorporating heparin in the internal oil phase of nano-emulsions via hydrophobic complexation with CPβCD. The cumulative release of heparin from SNEDDS after 120 min in simulated gastric fluid (pH 1.2) followed by 240 min in simulated intestinal fluid (pH 6.8) was 47.31%. While full release was not achieved, it was noted that a 6 h in vitro release profile is necessary to predict in vivo behavior, considering the physiological emptying time of the gastrointestinal tract. Additionally, the SNEDDS formulation could be further protected from gastric fluid by employing enteric-coated hard gelatin capsules, making it a promising candidate for oral delivery of heparin [73].

**Rebamipide**, a quinolinone derivative that enhances mucin production in corneal epithelial and conjunctival goblet cells, is currently administered as a suspension due to its poor solubility. Efforts to improve drug retention and solubility in corneal tissue remain ongoing. Otake et al. synthesized a β-CD polymer (CD-MSAm-co-QA, CDQA) by polymerizing co-monomers *N*,*N*,*N*-trimethyl-*N*-(2-hydroxy-3-methacryloyloxypropyl)-ammonium chloride (QA) and 6-deoxy-6-(2-methacryloyloxyethylsuccinamide)-β-cyclodextrin (CD-MSAm). CDQA was then used to form inclusion complexes with rebamipide (REB), and the potential of REB@CDQA was evaluated in a rabbit model for dry eye treatment. CDQA exhibited no corneal toxicity, as HCE-T cell viability remained above 90% at concentrations of 0.2–1%. The solubility of rebamipide in CDQA solution (10.27 μM) was significantly higher than in β-CD solution (3.8 μM). Furthermore, CDQA enhanced rebamipide penetration across the cornea. Instillation of REB@CDQA increased lacrimal fluid volume (1.3-fold) and mucin levels (1.5-fold) compared to the control (rebamipide suspension), with significant improvement in tear film stability [74].

Kaminski et al. synthesized two cationic derivatives of γ-cyclodextrin (γ-CD), namely, GCD-GTMAC and GCD-EDA, using glycidyltrimethylammonium chloride (GTMAC) and ethylenediamine (EDA), respectively. These derivatives were used to form inclusion complexes with the isoflavone **daidzein** (DAI). Both cationic GCD derivatives facilitated daidzein penetration across human fibroblasts and murine hippocampal neuronal cells. At a concentration of 100 μg/mL, the cellular glycosaminoglycan levels were reduced to 72% and 62% of the control for GCD-GTMAC/DAI and GCD-EDA/DAI, respectively. These findings suggest that cationic γ-CD derivative-based daidzein inclusion complexes may have potential applications in reducing glycosaminoglycan accumulation in mucopolysaccharidoses and lysosomal storage diseases [75]. All the studies discussed in this section have been summarized in Table S5.

## 4. Cationic Cyclodextrins for Nucleic Acid Delivery

**Figure 3:**
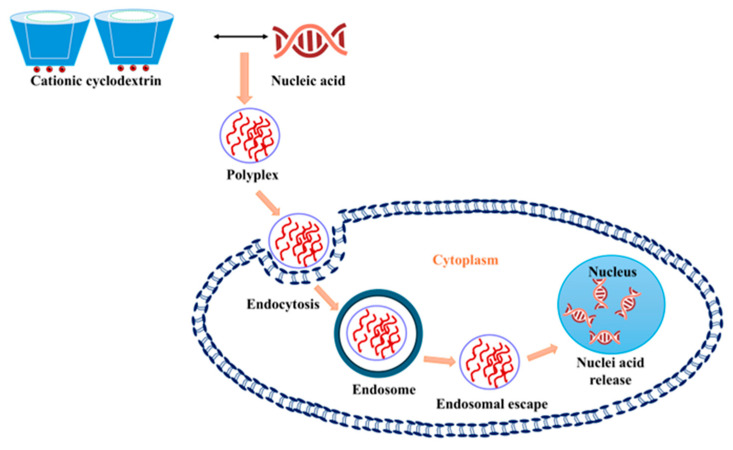
Mechanism of cationic cyclodextrin-mediated nucleic acid delivery.

Nucleic acids, such as plasmid DNA (pDNA), microRNA (miRNA), and small interfering RNA (siRNA), are emerging as promising therapeutic agents for treating acquired and genetic diseases, including cancer, cardiovascular conditions, monogenic disorders, and infectious diseases. However, these agents require targeted drug delivery systems due to their poor cellular uptake and rapid degradation in biological media [76]. These challenges can be addressed by using cationic cyclodextrins as carriers for nucleic acids [77,78]. Cationic cyclodextrins condense negatively charged nucleic acids into nano-sized particles known as polyplexes. These polyplexes are then internalized by cells through endocytosis, where the cyclodextrin polymers protect the nucleic acids from nuclease degradation and enhance their cellular uptake, leading to improved gene transfection efficiency (Figure 3) [79].

### 4.1. DNA

**Scheme 3:**
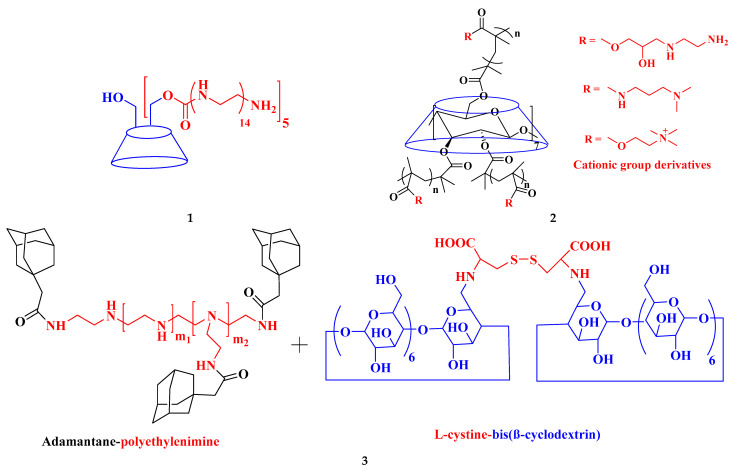

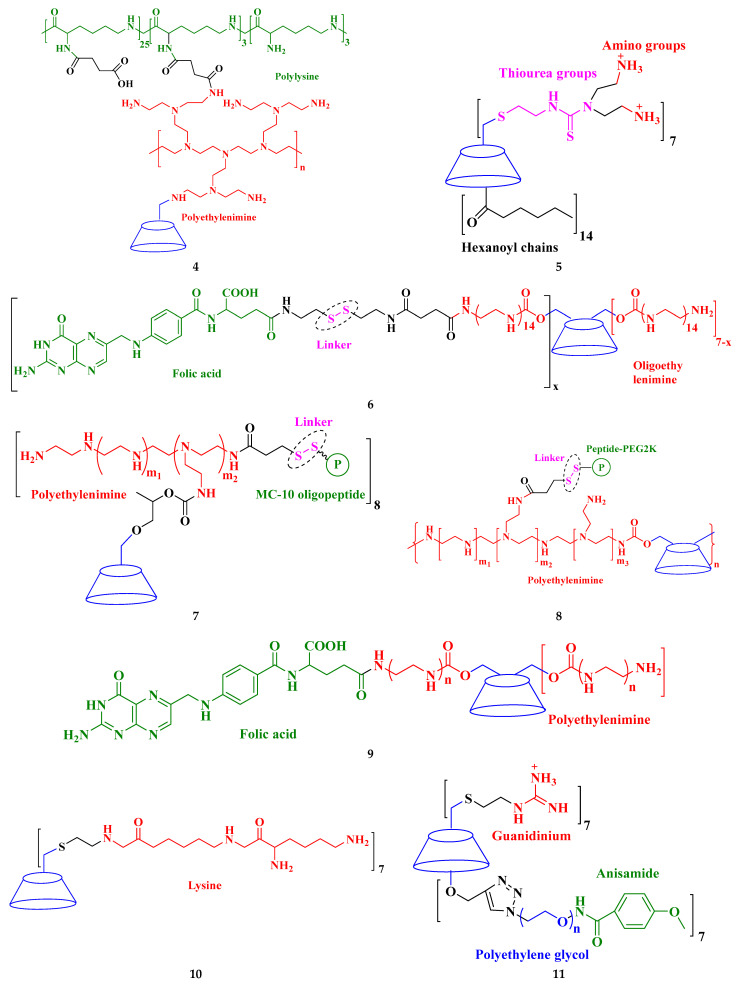

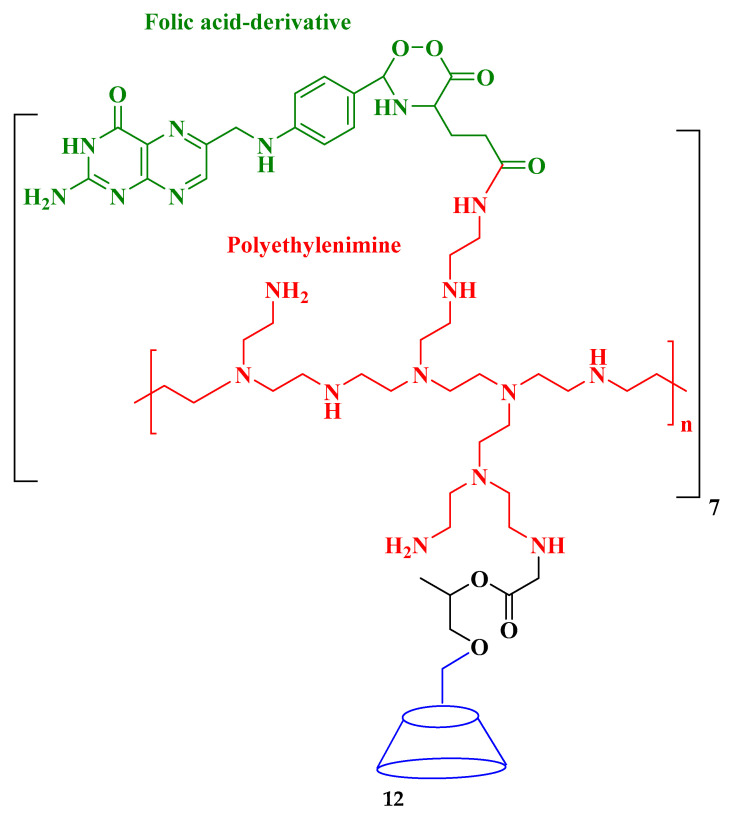
Chemical structures of cationic cyclodextrin-based systems employed for nucleic delivery.

Yang et al. fabricated a cationic star polymer (α-CD-OEI) by the grafting of oligoethylenimine (OEI) arms onto an α-cyclodextrin (α-CD) core (Scheme 3 (1)). The α-CD-OEI star polymer condensed the pDNA into 200 nm particles at a nitrogen/phosphate (N/P) ratio of 2 while PEI 25 kDa formed relatively larger polyplexes (390 nm) at the same N/P ratio. The star polymer exhibited 50-fold higher pDNA transfection efficiency in HEK293 cells than that of PEI 25 kDa in a serum-free environment; however, both agents showed comparable transfection efficiencies under serum conditions. Moreover, α-CD-OEI was less cytotoxic to HEK293 cells (~35% viability) as compared to PEI 25 kDa (~5% viability). The lower cytotoxicity of star polymer might be attributed to the reduced density of amino groups and the inclusion of α-cyclodextrin. It is considered that the high amino density and high molecular weight are responsible for the high cytotoxicity of PEI 25 kDa [80].

Li et al. fabricated a series of cationic star polymers with 21 arms (21ACSPs) by the polymerization of monomers containing primary, tertiary, and quaternary ammonium groups with Heptakis[2,3,6-tri-*O*-(2-bromo-2-methylpropionyl)]-β-cyclodextrin(21Br-β-CD) (Scheme 3 (2)). Subsequently, polyplexes were prepared from 21ACSPs and pDNA (luciferase plasmid (pCMV-Luc)) with a size range of 80–180 nm. 21ACSPs with primary and tertiary amino groups showed satisfactory transfection efficiency to CHSE-214 cells, i.e., ~23 and ~28 ng luciferase/mg protein. 

However, quaternary ammonium containing 21ACP was unable to show any transfection efficiency, as the quaternary ammonium cannot be further protonated in the acidic environment of the endosome. In this case, the polyplex might have entrapped within the endosome but it could not be decomposed within this organelle to mediate the DNA release. Moreover, transfection with 21ACSP/pDNA polyplexes showed high viability of CHSE-214 cells (77–88%), thus confirming their biocompatibility [81].

Zhang et al. coupled adamantane-grafted polyethylenimine with L-cystine-bridged bis(β-cyclodextrin) (PEI-Ada-LCD) (Scheme 3 (3)). The resultant supramolecular systems were employed for pDNA condensation, resulting in the formation of polyplexes with a 260–80nm size at 10-20 N/P ratio. The optimized PEI-Ada-LCD formulation showed almost twofold higher gene transfection efficiency (54%) than that of the gold standard, i.e., PEI 25 kDa (~32%), in 293T cells. Moreover, PEI-Ada-LCD was less cytotoxic toward 293T cells as compared to PEI 25 kDa. It was suggested that PEI-Ada-LCD contains enzyme-responsive disulfide bonds, which were cleaved by the reductive enzymes to mediate the DNA release [82].

Lv et al. fabricated epsilon-polylysine-grafted–succinic acid-grafted–β-cyclodextrin–polyethylenimine600 (PPC) (Scheme 3 (4)), which was entrapped with adamantane-functionalized poly-(ethylene glycol) derivative (PEG-AD). The resultant PPC/PEG-AD vectors could inhibit the migration of pDNA at an N/P ratio of 5 while bare PEI600 was unable to condense DNA at all tested N/P ratios. Moreover, compact polyplexes (200 nm) were formed by PPC/PEG-AD at a 20 N/P ratio. The pDNA transfection efficiency of PPC/PEG-AD was almost similar to PEI 25 kDa. However, PPC/PEG-AD showed extremely lower cytotoxicity to HEK293 cells (~95% viability) as compared to PEI 25 kDa (~10% viability) at an equivalent concentration [83].

The β-cyclodextrin derivative T2 contains 14 primary amino groups and 7 thioureido groups at the primary surface while having 14 hexanoyl chains at the secondary face (Scheme 3 (5)). Aranda et al. fabricated pDNA (luciferase-encoding plasmid DNA, pCMVLuc) polyplexes by employing T2 cyclodextrin derivatives (plain-CDplexes) that were further grafted with folic acid (fol-CDplexes). The fol-CDplexes were (263 nm) relatively larger than plain-CDplexes (254 nm) at a 5 N/P ratio. The fol-CDplexes showed significantly higher gene transfection efficiency than that of plain-CDplexes and standard PE1 25 kDa polyplexes in HeLa cells, i.e., ~68, ~38, and ~18 ng luciferase/mg protein, respectively. Both plain-CDplexes and Fol-CDplexes showed less cytotoxicity (above 80% viability) as compared to PEI 25 kDa (~70% viability). Further in vivo study demonstrated that gene expression induced by fol-CDplexes was 2-fold and 4.6-fold higher in liver and lung tissues, respectively, as compared to plain-CDplexes [84].

Cheng et al. developed the star-like amphiphilic β-cyclodextrin-graft-(poly(ε-caprolactone)-blockpoly(2-(dimethylamino) ethyl methacrylate)x (β-CD-g-(PCL-b-PDMAEMA)x)) copolymer, composed of a cyclodextrin core, hydrophobic poly(ε-caprolactone) PCL segments, and hydrophilic PDMAEMA blocks. The β-CD-g-(PCL-b-PDMAEMA)x was capable of condensing pDNA (220 nm) at a 0.5 N/P ratio, which is lower than previously reported for PEI 25 kDa. Moreover, β-CD-g-(PCL-b-PDMAEMA)x showed significantly higher pDNA transfection efficiency in RAW264.7 macrophages (10.8%) than that of standard lipofectamine (2.6%). Moreover, the copolymer showed low cytotoxicity (IC50 40 µg/mL), hence confirming its biocompatibility [51].

Zhao et al. fabricated cationic star-shaped polymers based on γ-cyclodextrin and oligoethylenimine, which were grafted with folic acid through disulfide bonds (γ-CD-OEI-SS-FA) (Scheme 3 (6)). The cationic γ-CD-OEI-SS-FA condensed pDNA to compact polyplexes (100 to 150 nm) at a 2.5 N/P ratio, which is the same as for standard PEI 25 kDa. The optimized γ-CDOEI-SS-FA formulation showed a sixfold higher gene transfection efficiency in KB cells as compared to standard PEI 25 kDa while imparting relatively less cytotoxic impacts. It was referred that folic acid ligand mediates the uptake of polyplexes to folate receptor-expressed KB cells where the disulfide linker of γ-CD-OEI-SS-FA is broken down by glutathione within the cells, resulting in efficient pDNA delivery [85].

Huang et al. synthesized cationic polymers by the coupling of polyethylenimine 600 Da (PEI) with hydroxypropyl-**γ**-cyclodextrin (HP-**γ**-CD). The polymeric carriers were grafted with MC-10 oligopeptide (P) to target human epidermal growth factor receptor 2 (HER2), which is overexpressed in several tumor cells. The resultant HP-**γ**-CD-PEI-P vectors (Scheme 3 (7)) condensed pDNA (pGL3-Luc) into 175 nm polyplexes at a 40 N/P ratio. The HP-**γ**-CD-PEI-P showed 4- and 3.7-fold higher pDNA transfection efficiency to SKOV-3 cells than that of PEI 25 kDa and non-functionalized HPg-CD-PEI, respectively. It was suggested that peptide conjugation promoted the interaction of HP-**γ**-CD-PEI-P/DNA complexes with HER2 receptors present on SKOV-3 cells followed by their internalization through receptor-mediated endocytosis. Moreover, HP-**γ**-CD-PEI-P did not show any noticeable cytotoxicity towards SKOV-3 cells up to a 120 N/P ratio while standard PEI 25 kDa showed less than 20% cell viability at a 40 N/P ratio. The less cytotoxic impact of HP-γ-CD-PEI-P might be attributed to the lower amino group density as compared to the branched PEI 25 kDa possessing high molecular weight [86].

### 4.2. RNA

Zeng et al. prepared cationic copolymers by linking ß-cyclodextrin with polyethylenimine (CD-PEI). Subsequently, polyethylene glycol (PEG) derivatives were prepared by coupling with matrix metalloproteinase (MMP)-2-cleavable peptide (C-PEG) and a non-cleavable peptide (unC-PEG). The resultant PEG derivatives were grafted onto CD-PEI, thus forming CD-PEI-C-PEG (Scheme 3 (8)) and CD-PEI-unC-PEG as vectors for miR-34a delivery. The CD-PEI, CD-PEI-C-PEG, and CD-PEI-unC-PEG transformed miR-34a into 54.83, 99.17, and 105.53 nm particles, respectively, at a 4 N/P ratio. The polyplexes formed by CD-PEI-C-PEG and CD-PEI-unC-PEG were relatively larger due to the presence of PEG along with peptides. The CD-PEI-C-PEG-based polyplexes were more efficiently internalized by 4T1 cells as compared to CD-PEI-unC-PEG. Moreover, the internalized polyplexes were capable of escaping the lysosome/endosome-mediated degradation. During the in vivo study, CD-PEI-C-PEG polyplexes exhibited significantly higher tumor inhibition potential (51.79%) than that of CD-PEI (7.01%) and CD-PEI-unC-PEG (5.08%). It was demonstrated that PEG coating reduces the nonspecific interaction between cationic particles and serum proteins and facilitates their accumulation in tumor tissues where the peptide is broken down by MMP-2, leading to miR-34a release [87].

Li et al. fabricated ß-cyclodextrin–polyethylenimine (ß-CD-PEI) conjugates that were coupled with folic acid (β-CD-PEI-FA) (Scheme 3 (9)). β-CD-PEIFA and β-CD-PEI condensed miR-34a-5p into 203.13 and 213.06 nm particles at a 5 N/P ratio. Furthermore, β-CD-PEI-FA and ß-CD-PEI prevented the miR-34a-5p degradation after RNase, heparin, and fetal bovine serum treatment. This indicates that both carriers could effectively protect miR-34a-5p from serum and nuclease degradation. As compared to the control, β-CD-PEI-FA showed 306.12 and 2.73 times higher miR-34a-5p expression in BCBL-1 and SK-RG cells while β-CD-PEI exhibited 353.67 and 2.19 times higher expression for both cells. Moreover, β-CD-PEIFA and β-CD-PEI showed up to 70% viability of BCBL-1 and SK-RG cells, hence confirming their biocompatibility [88].

Fitzgerald et al. synthesized a cationic derivative by the attachment of two lysine amino acids on the primary surface of β-cyclodextrin (Scheme 3 (10)). Subsequently, a PEGylated adamantane derivative (PEG-Ad-AA) was prepared containing anisamide-targeting ligand, which selectively interacts with sigma receptors overexpressed on the surface of prostate cancer cells. The dilysine β-cyclodextrin was employed for the complexation of siRNA at a 10 N/P ratio followed by treatment with PEG-Ad-AA, forming 288.9 nm particles. The resultant polyplexes were efficiently internalized by prostate cancer cells (DU145, VCaP, and PC3 cells) while maintaining >80% cell viability. Further, competitive uptake inhibition analysis confirmed that uptake of these nanoplexes was induced through sigma receptor-mediated endocytosis. The nanoplexes exhibited efficient silencing of the PLK1 gene, which is involved in numerous types of cancers. The carriers protected siRNA from nuclease digestion for up to 24 h, while naked siRNA was partially degraded within 8 h [89].

Guo et al. attached guanidinium on the primary surface and a polyethylene glycol chain on the secondary surface of β-cyclodextrin (G-CD-PEG) while the resultant conjugate was attached with anisamide (G-CD-PEG-AA) (Scheme 3 (11)). The complete complexation of siRNA was carried out by G-CD-PEG and G-CD-PEG-AA at N/P ratios of 50 and 75, respectively, forming ~210–260 nm particles. In contrast to non-targeted carriers, the G-CD-PEG-AA was efficiently internalized by PC-3 cells (sigma receptor-positive), i.e., 65–70% fluorescein-positive cells. Moreover, G-CD-PEG-AA was unable to be taken up by CT26 cells (sigma receptor-negative). This indicates that cellular internalization of G-CD-PEG-AA is mediated by sigma receptor-mediated endocytosis. Moreover, G-CD-PEG-AA induced prostate cell-specific internalization of siRNA resulting in approximately 80% knockdown luciferase (reporter gene). Followed by intravenous administration, G-CD-PEG-AA/vascular endothelial growth factor (VEGF) siRNA exhibited a threefold reduction in tumor volume as compared to PBS while non-targeted carriers were unable to demonstrate a noticeable anti-tumor effect [90]. 

Li et al. prepared conjugates based on polyethylenimine and 2-hydroxypopyl-β-cyclodextrin (PEI-HP-β-CD), which were functionalized with folic acid (FA-PEI-HP-β-CD) (Scheme 3 (12)). FA-PEI-HP-β-CD completely condensed the siRNA into 250 nm particles at a 24 N/P ratio. FA-PEI-HP-β-CD/siRNA were internalized by folate receptor-enriched HeLa cells to a greater extent (fourfold) than non-targeted PEI-HP-β-CD while maintaining 90% cell viability. FA-PEI-HP-β-CD protected up to 90% of siRNA from nuclease degradation within 12 h while naked siRNA was completely degraded during the specified period. During the in vivo study, FA-PEI-HP-β-CD/siVEGF showed a better reduction in tumor volume (~600 mm^3^) as compared to non-targeted PEI-HP-β-CD/siVEGF (~1000 mm^3^) and control (~1700 mm^3^). Moreover, VEGF expression was significantly reduced by FA-PEI-HP-β-CD/siVEGF (35.7%) in comparison to PEI-HP-β-CD/siVEGF (72.5%) and control (100%). These findings indicate that FA-PEI-HP-β-CD was taken up by folate receptor-mediated endocytosis and released siRNA into cancer cells, which induced VEGF gene silencing, resulting in better anti-tumor potential [91]. Li et al. fabricated cationic star-shaped polymers consisting of a β-cyclodextrin core and poly(amidoamine) dendron arms (β-CD-PAMAM). The resultant carriers blocked the migration of MMP-9-siRNA at a 4 N/P ratio while the size of the polyplexes varied from 268 nm to 110 nm with an increasing N/P ratio from 6 to 60. β-CD-PAMAM and standard lipofectamine 2000 exhibited transfection efficiency of 98.78% and 64.89% while inducing 5.57% and 1.21% fibroblast cell death. Furthermore, MMP-9 expression was reduced by 68% and 80% for the cells treated with β-CD-PAMAM/MMP-9-siRNA and lipofectamine 2000/MMP-9-siRNA, respectively. β-CD-PAMAM/MMP-9-siRNA-treated diabetic rats showed >50% wound closure within seven days. Diabetic wounds are characterized by the overexpression of MMP-9; hence, the downregulation of MMP-9 mediated by β-CD-PAMAM-based polyplexes was attributable to the wound healing activity [92]. Further studies based on cationic cyclodextrin-mediated nucleic delivery are summarized in Table 4 (Table S6).

## 5. Cationic Cyclodextrins for Combinatorial Delivery of Drugs and Nucleic Acids

**Scheme 4:**
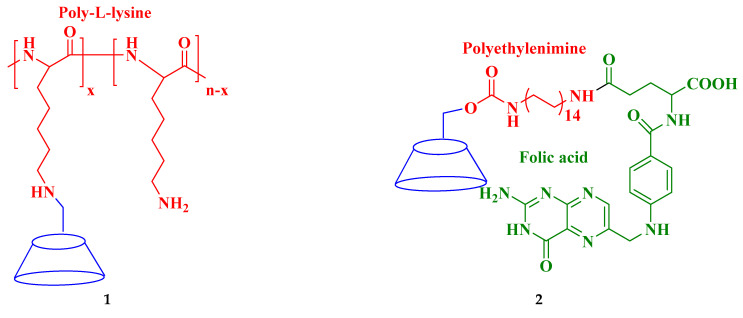

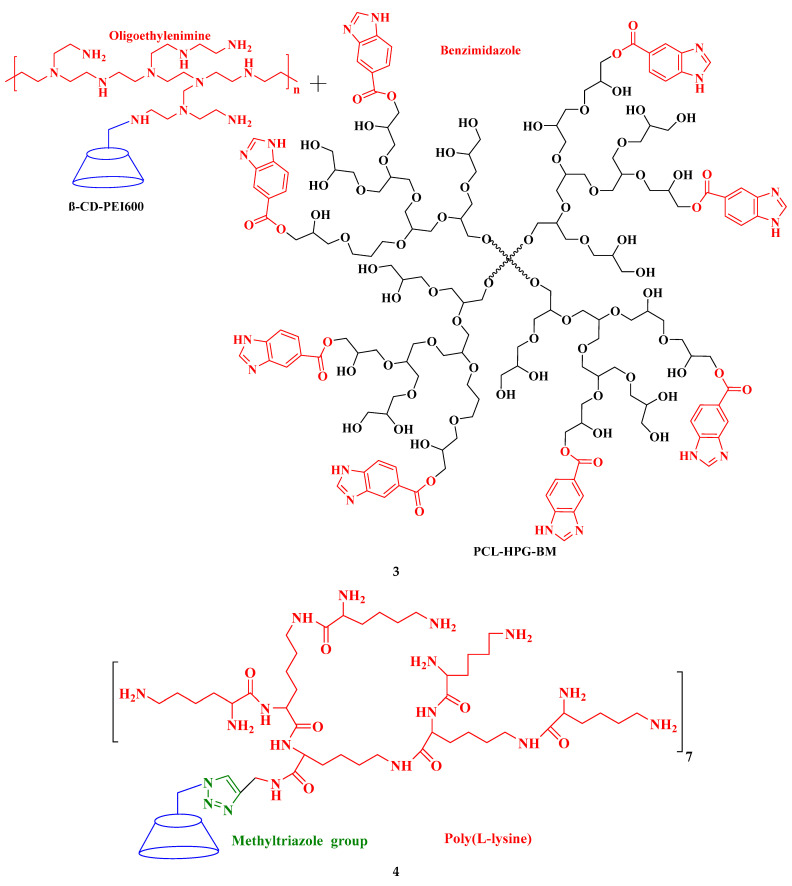

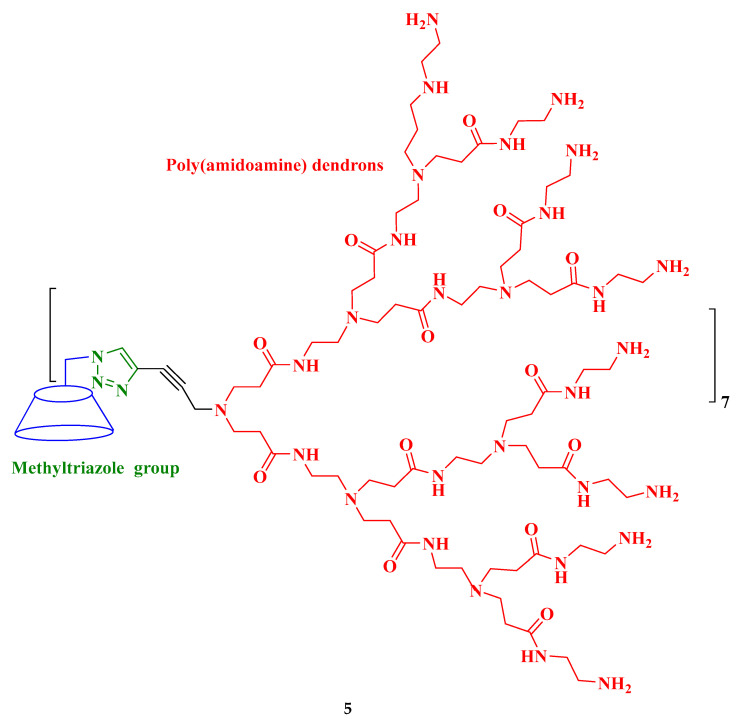
Chemical structures of cationic cyclodextrin-based systems employed for co-drug and nucleic acid delivery.

Cationic cyclodextrins can hold two or more payloads with variable properties. The surface hydroxyl groups can be exploited for one payload while cyclodextrin pocket can entrap a second payload [11]. Hence, cyclodextrins have been employed for the simultaneous delivery of drugs and nucleic acids. Xiong et al. fabricated β-cyclodextrin and poly-L-lysine conjugates (Scheme 4 (1)) that were entrapped with DOX via host–guest interactions. The resultant nanocomplexes were employed to condense oligoRNA through electrostatic interactions (PDR) at an N/P ratio of 30 and grafted with hyaluronic acid (HPDR) forming 195.8 nm particles. During the in vitro study, HPDR NPs were efficiently taken up by MHCC-97H cells as compared to PDR NPs. It was demonstrated that hyaluronic acid interacted with CD44 receptors on MHCC-97H cells, thus mediating their internalization through CD44-mediated endocytosis. As a result, HPDR NPs showed higher toxicity to MHCC-97H cells (IC_50_ 6.58 µg/mL) than that of PDR NPs (IC_50_ 11.26 µg/mL). Subsequently, MHCC-97H tumor-bearing mice were treated with free Cy5.5 dye, PDR-Cy5.5, and HPDR-Cy5.5 NPs. Free Cy5.5 was rapidly cleared from the body of the mouse while PDR-Cy5.5 NPs were distributed both in the liver and tumor tissues after 24 h. However, HPDRCy5.5 NPs were mostly distributed in the tumor tissues indicating that hyaluronic acid functionalization enabled the NPs to escape from the recognition of reticuloendothelial system and promoted the release of payload at the target site [103].

Mousazadeh et al. fabricated folate-appended polyethylenimine–β-cyclodextrin (FPC) (Scheme 4 (2)) and benzimidazole-modified magnetic–hollow mesoporous silica NPs (BM-MHMSNs). DOX was first encapsulated in BM-MHMSNs and capped by FPC as smart nano-gates. Subsequently, the FPC polycationic gatekeepers formed electrostatic complexes with human telomerase reverse transcriptase (hTERT) siRNA, thus forming FPC/BM-MHMSNs/DOX/siRNA NPs, i.e., 178 nm at an N/P ratio of 20. Folic acid was grafted on the surface of these NPs as a tumor-targeting ligand. The FPC/BM-MHMSNs/DOX/siRNA NPs were efficiently taken up by MCF-7/ADR cells, showing the highest fluorescence intensity among all treated groups. Free DOX was unable to impart cytotoxic impact (IC_50_ > 500 µg/mL) while FPC/BM-MHMSNs/DOX/siRNA NPs showed remarkable cytotoxicity towards MCF-7/ADR cells (IC_50_ > 6.20 µg/mL), which was further increased under the influence of an alternating magnetic field (IC_50_ > 4.42 µg/mL).

Moreover, non-functionalized PC/BM-MHMSNs/DOX/siRNA NPs showed lower knockdown efficiency of hTERT protein (37%) than FPC/BM-MHMSNs/DOX/siRNA carriers (68%) [104].

Zhou et al. fabricated oligoethylenimine-conjugated β-cyclodextrin (β-CD-PEI600) and benzimidazole-modified forearm polycaprolactone-initiated hyperbranched polyglycerol (PCL-HPG-BM) (Scheme 4 (3)). DOX was encapsulated into the hydrophobic core of PCL-HPG-PEI600 while MMP-9 shRNA plasmid (pMMP-9) was attached through electrostatic interaction between PEI600 segment and pMMP-9 thus forming a co-delivery carrier (PCL-HPG-PEI/DOX/pMMP-9). The PCL-HPG-PEI600/pMMP-9 formed ~220 nm particles at 20 N/P ratio. Moreover, PCL-HPG-PEI600/pMMP-9 exhibited >50% transfection efficiency to MCF-7 cells while standard PEI 25 kDa showed only 17% transfection efficiency. The co-delivery carrier PCL-HPG-PEI/DOX/pMMP-9 showed better inhibition of MCF-7 cell proliferation (IC_50_ 1.95 µg/mL) than that of bare PCLHPG-PEI600/DOX (IC_50_ 2.61 μg/mL). Tumor-bearing mice treated with PCL-HPG-PEI/DOX/pMMP-9 showed only 21% tumor volume as compared to PBS [105].

Ma et al. conjugated 6-azido-β-cyclodextrin with poly(L-lysine) dendron (CD-PLLD) (Scheme 4 (4)), which formed electrostatic complexes with pDNA, into ~200 nm particles at an N/P ratio of 20. The CD-PLLD/pDNA exhibited >30% transfection efficiency to MCF cells. Subsequently, MTX was loaded to CD-PLLD with higher entrapment efficiency (>20%) as compared to the bare 6-azido-β-cyclodextrin (~17%) and poly(L-lysine) dendron (10%). Moreover, CD-PLLD released MTX in a more sustained manner (~45%) than 6-azido-β-cyclodextrin (~50%) and poly(L-lysine) dendron (~70%) within 25 h. CD-PLLD-MTX-treated MCF cells exhibited < 70% viability at 50 µg/mL concentration. It was concluded that CD-PLLD could be employed for the co-delivery of nucleic acid and lipophilic anticancer drugs without a complicated micellization process [106].

During a subsequent study, Liu et al. employed CD-PLLD (Scheme 4 (4)) as a carrier for docetaxel (DOC) and MMP-9 siRNA plasmid (pMR3) forming 125 nm particles at an N/P ratio of 20. CD-PLLD/pMR3 exhibited >26% transfection efficiency to HNE-1 cells. The CD-PLLD/DOC/pMR3-treated HNE-1 cells exhibited a 50% reduction of MMP-9 mRNA expression as compared to PBS. Moreover, CD-PLLD/DOC/pMR3 showed greater apoptosis of HNE-1 cells (55.5%) as compared to CD-PLLD/DOC (13.8%) and CD-PLLD/pMR3 (40.7%). The greater anti-tumor potential of CD-PLLD/DOC/pMR3 was attributed to the fact that released DOC could induce DNA damage while pMR3 could mediate the downregulation of protein expression. Moreover, bare CD-PLLD showed >90% cell viability while standard PEI 25 kDa showed <50% viability, hence confirming the biocompatibility of CD-PLLD. Further in vivo studies also confirmed the non-observed toxicity of CD-PLLD, which might be due to its lower molecular weight (<5 kDa), biodegradable nature, and presence of positive charge on the surface [107].

Deng et al. fabricated star-shaped polymers by the coupling of per-6-azido-β-cyclodextrin with poly(amidoamine) dendrons (β-CD-(D3)7) (Scheme 4 (5)) for the co-delivery of siRNA and MTX. β-CD-(D3)7/siRNA complexes were formed at an N/P ratio of 30 with 100 to 200 nm size. β-CD-(D3)7/siRNA complexes showed better transfection efficiency to fibroblast cells than PAMAM dendrimers, i.e., ∼85% vs. ∼75% with serum and ~97% vs. ~82% without serum. Moreover, β-CD-(D3)7/siRNA complexes maintained high cell viability (54%) as compared to PAMAM dendrimers (35%). β-CD-(D3)7/siRNA complexes released the DOX in a sustained manner for up to 10 h. The controlled drug release might be attributed to the hindrance of conjugated D3 arms to the diffusion of the MTX entrapped in the β-CD core [108]. All studies discussed in this section are summarized Table S7. Moreover, we have also categorized all drug and nucleic acid delivery examples included in this manuscript based on the type of cationic agents in Table S8.

## 6. Challenges and Research Gaps Associated with Cationic Cyclodextrin-Based Delivery Systems

The development of cationic cyclodextrin-based systems for drug and gene delivery is an emerging area that is still encountering some challenges. A wide range of polymerization reactions can be employed for the synthesis of cationic cyclodextrin polymers such as chemical oxidative polymerization, ring-opening polymerization, radical polymerization, azide–alkyne polymerization, polycondensation of functionalized cyclodextrins with difunctionalized comonomers, nucleophilic substitution polycondensation, and Menshutkin reaction [20]. However, the synthesis and characterization of ionic cyclodextrin polymers may be hindered due to the complexity and high cost of the process. Hence, there is still room for further improvements in synthetic and characterization strategies.

Achieving precise control over the charge density and hydrophobicity of cationic cyclodextrins remains a significant challenge [109]. Further research is needed to enable the controlled and reproducible incorporation of cationic groups into the cyclodextrin scaffold. This will require the development of characterization techniques capable of providing detailed information about the chemical composition, degree, and pattern of substitution, as well as the molar mass and distribution of polymeric cationic cyclodextrins [21]. Additionally, a thorough understanding of the acid–base behavior of polybasic cyclodextrin monomers and polymers is crucial, as the charge state plays a key role in small molecule encapsulation, membrane interaction, and polyplex formation. Currently, cationic cyclodextrins are expensive, as they are synthesized by only a few research groups and companies. Therefore, there is a need for more efficient and cost-effective synthesis methods on a commercial scale. The application of cyclodextrins in vaccines is expected to significantly accelerate their development.

Among the thousands of cyclodextrin derivatives (cationic, anionic, and non-ionic) described in scientific publications and patents, only a small fraction is viable for industrial-scale synthesis and application. Most derivatives are synthesized through complex multistep reactions that rely on expensive, toxic, and environmentally harmful reagents and solvents. These processes often require extensive purification, typically achieved through chromatography on a laboratory scale. However, chromatography is impractical and prohibitively expensive for large-scale production.

Moreover, industrial-scale synthesis necessitates the adoption of “green” synthetic methodologies, which are particularly challenging in carbohydrate chemistry due to the complexity of cyclodextrin structures. Overcoming the reliance on common reagents and solvents used in laboratory processes presents additional hurdles. As a result, only about a dozen cyclodextrin derivatives can currently be considered feasible for production at scale, ensuring acceptable costs and environmental impact [110,111].

Another challenge associated with cationic cyclodextrins is the poor understanding of their mechanism of action and interaction with biological components at the molecular level. The effectiveness of cationic cyclodextrin-based delivery systems has mainly been investigated through in vitro models including specific cell lines or bacterial strains, limiting the direct applicability of these findings to humans or animals [109]. Hence, in vivo studies should be carried out to explore the safety, biocompatibility, and biodistribution profiles of cationic cyclodextrins [112]. The impact of particle size, polydispersity index, and other related characteristics on the biocompatibility of cyclodextrins should also be investigated. Moreover, long-term studies are still lacking to investigate stability, safety, and long-term gene expression. Cationic cyclodextrin-based delivery systems have not yet been extensively entered into the clinical trial phase, which is pivotal for determining their safety and efficacy in humans [109]. These challenges highlight the need for further detailed studies to translate lab findings into real-world applications.

## 7. Conclusions

The current review elaborates the recent developments in the cationic cyclodextrin-mediated delivery of drugs and nucleic acids. Cationic cyclodextrin-based delivery systems have been extensively employed for the delivery of anticancer, anti-inflammatory, and antibacterial drugs; however, limited data are available for antidiabetic drug delivery. During most of these studies, cationic cyclodextrins improved drug solubility, stability, and permeability across biological membranes and ensured site-specific drug release, resulting in outstanding therapeutic outcomes. Cationic cyclodextrin-based delivery systems have also been employed for nucleic acid delivery, including pDNA, miRNA, siRNA, etc. Cationic cyclodextrins showed efficient nucleic condensation and improved transfection efficiency while maintaining high cell viability as compared to commercially available vectors such as PEI25 kDa and lipofectamine 2000. Moreover, cationic cyclodextrins have also shown promising potential for the co-delivery of drugs and genes.

## Figures and Tables

**Table 1:** Development of cationic cyclodextrin-based systems for anticancer drug delivery.

Drug	Cationic Motif	Type of Cyclodextrin	Type of Formulation	Outcomes	References
Doxorubicin (DOX)	Polyethylenimine (PEI)	β-cyclodextrin (β-CD)	Ternary conjugates of bisphosphonate and PEI β-CD	In vitro study demonstrated the mitochondrial targeting and potential to induce targeted drug delivery to different bone-related cancer cells, i.e., MG-63 and MDA-MB-231, resulting in superior cytotoxicity. The in vivo evaluation also confirmed the targeting of these conjugates in xenograft mice bearing MG-63 and MDA-MB-23 cells.	[26]
Methotrexate (MTX)	Poly (2-(dimethylamino) ethyl methacrylate) (PDMAEMA)	β-cyclodextrin	Hydrogels	Hydrogels showed higher cytotoxicity (IC_50_ 27 μg/mL) towards MCF-7 cells than free MTX (IC_50_ 55 μg/mL) and showed a pH-sensitive drug release profile (32.56% at pH 7.4 vs. 85.3% at pH 5.2).	[42]
Doxorubicin (DOX)	Quaternary ammonium	β-cyclodextrin	Nanoparticles (NPs)	Cationic cyclodextrin NPs improved the permeability of DOX across the * BBMVEC monolayer up to 2.2-fold. NPs were less cytotoxic to the BBMVEC monolayer than free DOX. Moreover, NPs killed U87 tumor cells as effectively as bare DOX.	[37]
4-hydroxy-tamoxifen (TMX)	Quaternary ammonium	β-cyclodextrin	Cationic poly(cyclodextrin)/ alginate nanocapsules	In vitro study demonstrated that nanocapsules can efficiently deliver the TMX to immortalized mouse podocyte cells, which mediates Cmip exon 8 deletion.	[43]
5-Fluorouracil (5-FU)	Quaternary ammonium	β-cyclodextrin	Cationic β-CD loaded alginate/chitosan nanoflowers	The nanoformulation showed a pH-dependent and sustained 5-FU release profile up to 24 h. Further, in vivo studies are still in progress to evaluate the effectiveness of these nanoflowers in animal models.	[44]
Camptothecin (CPT)	Quaternary ammonium	Polycationic ß-cyclodextrin derivative (PC ßCDC6)	Nanoparticles (NPs)	NPs showed a promising mucus layer penetration rate (73%) and released a smaller CPT content (48%) in ** SGF and *** SIF, hence demonstrating the potential for colon-targeted drug delivery. Moreover, nanoformulation reduced the viability of HT-29 cells (52.44%) to a greater extent than free CPT solution (83.98%) at an equivalent concentration (0.1 µg/mL).	[45]
Melphalan (MLP)	Gemini surfactant	β-cyclodextrin	Inclusion complexes	MLP complexes showed a significantly reduced IC_50_ compared to free MLP (27 µM vs. 98 µM) against A375 cell lines. Hence, the inclusion of MLP with cationic cyclodextrin can improve its efficacy.	[35]
Scutellarin (SCU)	Poly(ε-lysine) (ε-PL)	β-cyclodextrin (β-CD)	ε-PL and glycine β-CD (ε-PL-GLY-CD) based inclusion complexes	ε-PL-GLY-C exhibited improved aqueous solubility of SCU (52.82 mg/mL) compared to bare SCU (0.16 mg/mL). Moreover, these inclusion complexes exhibited higher cytotoxicity against HCT116 and LOVO cells (IC_50_ 8.2 and 19.4 µM) as compared to bare SCU (IC_50_ 72.3 and 80.6 µM), respectively.	[29]
Scutellarin (SCU)	Triethylenetetramine	β-cyclodextrin	Inclusion complexes	Complexes showed improved water solubility (50.7 mg/mL) over that of free SCU (0.16 mg/mL). Furthermore, the cytotoxic potential of these inclusion complexes was remarkably higher towards HCT116 and LOVO cells (IC5_0_ 0.9, 8.3 µM) than that of free SCU (IC_50_ 72.3, 80.6 µM), respectively.	[46]

Abbreviations: * BBMVEC—bovine brain microvascular endothelial cell; ** SGF—simulated gastric fluid; *** SIF—simulated intestinal fluid.

**Table 2:** Development of cationic cyclodextrin-based systems for anti-inflammatory drug delivery.

Drug	Cationic Motif	Type of Cyclodextrin	Type of Formulation	Outcomes	References
Indomethacin (IDM)	Chitosan (CS)	β-cyclodextrin	Electrospun nanofibers	Nanofibers showed a sustained release of IDM and equilibrium was attained within 125 h while ~80% payload was liberated within 300 h. Moreover, nanofibers were biocompatible, as the growth of L929 cells was not inhibited to a significant extent.	[28]
Indomethacin (IDM)	Quaternary ammonium	β-cyclodextrin	Hydrogel	The solubility of IDM (0.0615 mmol/L) was 100-fold enhanced after complexation with cationic β-CD (6.5 mmol/L). In simulated gastrointestinal fluid, the hydrogel was completely swollen within 14-16 h and 100% of IDM was released within 21 h. Hence, these hydrogels can serve as a controlled release system for IDM.	[7]
Dexamethasone (DXM)	Poly (2-(dimethylamino) ethyl methacrylate) (PDMAEMA)	β-cyclodextrin (β-CD)	β-CD-graft-(poly(ε caprolactone)-block-PDMAEMA) (β-CD-g-(PCL-PDMAEMA)) star-like amphiphilic polymer-based inclusion complex	β-CD-g-(PCL-b-PDMAEMA)-loaded DXM efficiently reduced the lipopolysaccharide-mediated release of cytokines (interleukin-1β, interleukin-6, and interleukin-10) in RAW264.7 macrophages compared to free DXM.	[51]
Naproxen (NPX)	Quaternary ammonium	β-cyclodextrin (β-CD)	Inclusion complexes	The cationic β-CD complexation improved the aqueous solubility of NPX up to 120-fold. Conjugates exhibited a faster dissolution rate and higher amount of drug dissolution. Moreover, cationic β-CD exhibited relatively low hemolytic activity compared with parent β-CD, hence confirming the biocompatibility of these drug carriers.	[6]
Ketoprofen (KTP)	Chitosan	β-cyclodextrin	Nanoparticles (NPs)	NPs exhibited a sustained KTP release profile in * PBS (at pH 7.4) and attained the equilibrium after 23 h while bare chitosan nanoparticles liberated the entire payload within 9 h. Moreover, NPs were considered biocompatible based on the high viability of L929 cells.	[52]
Diclofenac sodium (DCF)	Amino groups	Poly-β-amino-cyclodextrin derivative	Nanoassemblies	PolyCD-based nanoassemblies were efficiently taken up by ** hMSCs (within 2 h) without imparting any cytotoxic impact. Subsequently, interleukin-1β and tumor necrosis factor-α levels were reduced to a significant extent as compared to other treatment groups. Moreover, DCF was released in a controlled manner (~35% within 10 days).	[1]
Ibuprofen (IBU)	K^+^ cations	β-cyclodextrin	Metal–organic frameworks (MOFs)	The aqueous solubility of IBU entrapped in MOFs was improved up to 17-fold.	[53]
Niflumic acid (NIF)	K^+^ cations	γ-cyclodextrin	Metal–organic frameworks (MOFs)	MOFs showed a pH-dependent drug release, i.e., 14% NIF in 1 h and 40% NIF in 6 h at pH 1.6, while 40% NIF was released in 2 h and the remaining amount was completely released in 8 h. NIF incorporated in the frameworks also showed improved solubility.	[54]
Ketotifen hydrogenfumarate (KHF)	*** HHDDP surfactant	γ-cyclodextrin (γ-CD)	Hyaluronic acid (HA)/γ-CD/HHDDP polymer assemblies	The water-insoluble polymer matrix released KHF (water-soluble drug) slowly while free KHF was completely dissolved in a physiological saline solution over 35 min. Hence, this delivery system can be employed to attain a sustained release profile for water-soluble drugs.	[18]

Abbreviations: * PBS—phosphate-buffered saline, ** hMSCs—human mesenchymal stromal cells, *** HHDDP—[hexadecyl(2-hydroxyethyl)dimethylammonium dihydrogen phosphate].

**Table 3:** Development of cationic cyclodextrin-based systems for antibacterial drug delivery.

Drug	Cationic Motif	Type of Cyclodextrin	Type of Formulation	Outcomes	References
Sulfadiazine (SSD) Sulfamonomethoxine (SMMX) Sulfamethoxazole (SMZ)	Chitosan (CS)	β-cyclodextrin (β-CD)	Inclusion complexes based on CD grafted with β-CD (CD-g-CS)	The aqueous solubilities of free SSD, SMMX, and SMZ were noticed as 0.026, 0.069, and 0.329 mg/mL, respectively. However, CD-g-CS-based SSD, SMMX, and SMZ complexes showed significantly improved solubilities up to 5.6-fold (0.147 mg/mL), 4.1-fold (0.283 mg/mL), and 2.3-fold (0.747 mg/mL), respectively.	[61]
Levofloxacin (LVX)	Chitosan (CS)	β-cyclodextrin (β-CD)	CS-β-CD inclusion complexes	Simple β-CD conjugates released 100% LVX in 180 min while CS-β-CD complexes released the complete payload in 800 h. Hence, CS-β-CD complexation provided a fourfold slower drug release in comparison to bare β-CD. These complexes can serve as sustained drug delivery systems.	[62]
Linezolid (LZD)	Amino groups	β-cyclodextrin	Au@Ag bimetallic nanoparticles (BMNPs)	MIC values of LZD loaded NPs and free LZD were noticed as 2 vs. 1.95 µg/mL for * MRSA, 2 vs. 0.97 for *S. aureus*, 2 vs. 15.6 µg/mL for *E. coli*, and 3.9 vs. 15.6 µg/mL for *P. aeruginosa*. Hence, LZD nanoformulation showed a broad spectrum of activity than free LZD.	[63]
Triclosan (TR)	Quaternary ammonium	β-cyclodextrin	Inclusion complexes	The water solubility of TR increased linearly until the solubility limit was achieved, i.e., 1.4 mg/mL TR with 3.8 mg/mL cationic β-CD. Moreover, cationic β-cyclodextrin polymers were considered biocompatible due to the high viability (99%) of human colon carcinoma Caco-2 cells.	[64]
Triclosan (TR) and Butylparaben (BP)	Quaternary ammonium	β-cyclodextrin (β-CD)	Inclusion complexes	The *E. coli* growth inhibition potential of TR/β-CD was higher than that of BP/β-CD at lower than 0.5% concentration, while above 0.5% concentration, both antibiotics exhibited 100% inhibition of bacterial growth. Hence, TR/β-CD complex was more potent than BP/(β-CD). However, BP complex showed a faster growth inhibition rate than the TR complex in a short contact time (10 min).	[65]
Nitric oxide (NO)	Poly(amidoamine) dendron (PAMAM)	β-cyclodextrin (β-CD)	β-CD-PAMAM/NO inclusion complexes	β-CD-PAMAM/NO complexes (50 µg/mL) showed 80% and 94% biofilm inhibition for *E. coli* and *S. aureus* within 18 h. Moreover, β-CD-PAMAM/NO exhibited excellent biocompatibility and did not impart any cytotoxic impact on NIH 3T3 cells.	[32]
α-mangostin (α-MG)	Quaternary ammonium and chitosan	β-cyclodextrin (β-CD)	Quaternized β-CD-grafted chitosan (QCD-g-CS)-based inclusion complexes	QCD-g-CS/α-MG complexes showed an initial burst release of α-MG (up to 5 h) followed by sustained release (up to 24 h). QCD-g-CS/α-MG showed significantly lower MIC, i.e., 0.6 and 1.25 mg/mL, for *Streptococcus mutans* and *Candida albicans* while bare α-MG showed >10 mg/mL MIC for both microbes.	[66]

Abbreviations: * MRSA—methicillin-resistant *Staphylococcus aureus*.

**Table 4:** Development of cationic cyclodextrin-based systems for nucleic acid delivery.

Nucleic Acid	Cationic Motif	Type of Cyclodextrin	Description of Vector	Size of Polyplexes	Outcomes	References
pDNA	Dimethylaminoethyl (DMAE)	α-cyclodextrin (α-CD)	Polyrotaxanes based on cationic α-CD and disulfide-grafted poly(ethylene glycol) (PEG)	178-189 nm at 0.5 * N/P ratio	The polyrotaxanes formed stable polyplexes at a 0.5 N/P ratio with positive zeta potential (+4.8 mV) while ** LPEI22k was unable to form compact polyplex and showed negative zeta potential. Moreover, polyrotaxane exhibited concentration-dependent transfection efficiency and precluded lysosomal degradation of pDNA due to the proton sponge effect.	[93]
pDNA	Oligoethylenimine (OEI)	α-cyclodextrin (α-CD)	Fe_3_O_4_ nanoparticles functionalized with α-CD-OEI	120 nm at 20 N/P ratio	The implication of magnetic field improved the gene transfection efficiency of nanocarriers up to 10-fold. Magnetic field mediated the accumulation of polyplexes on the cell membrane and facilitated their penetration into the cell.	[94]
siRNA	*N*,*N*′-dimethylethylenediamine (DMEDA)	α-cyclodextrin (α-CD)	α-CD/poly(ethylene glycol) polyrotaxanes	150–200 nm at 5 N/P ratio	The cationic polyrotaxanes exhibited >10^2^-fold lower cytotoxicity than the standard PEI 25 kDa. Moreover, polyrotaxanes showed comparable gene silencing efficiencies (60–70% reduced *** GFP expression) to those of lipofectamine 2000 and PEI 25 kDa.	[95]
siRNA	Ionene	α-cyclodextrin + β-cyclodextrin	Polyrotaxanes	127 nm at 2 N/P ratio	Polyrotaxanes mediated the siRNA internalization into A549 cell line (10 times higher fluorescence intensity than that of original cells) while exhibiting 60% knockdown efficiency of luciferase gene.	[96]
DNA	Polyethylenimine 600 (PEI 600)	β-cyclodextrin (β-CD)	Amphiphilic β-CD-based vectors	116.6, 96.6, and 100.2 nm at N/P ratio of 5, 10, and 30	PEI600-β-CD showed better gene transfection efficiency (~110% vs. ~80% relative to control) than that of PEI 25 kDa in 10% serum while both carriers showed almost similar efficiency, i.e., 100% in a serum-free environment. Moreover, PEI600-β-CD were less cytotoxic to 293T cells at all the tested concentrations (10–100 µg/mL), i.e., ~60–90% viability vs. ~50–80%. Polyplexes were up-internalized through caveolae-mediated endocytosis, which could prevent the lysosomal degradation of entrapped genes.	[97]
pDNA	Poly(2-(dimethylamino) ethyl methacrylate (PDMAEMA)	β-cyclodextrin (β-CD)	β-CD-grafted poly(ε-caprolactone)–PDMAEMA copolymer-based vectors	218 nm at 2 N/P ratio	β-CD-based vectors showed significantly higher gene transfection efficiency than that of **** PEI 25 kDa, i.e., 84% vs. 52.9% in HEK293T cells and 23.6% vs. 10.6% in HepG2 liver cancer cells. Moreover, β-CD based vectors were less cytotoxic to both cell lines (up to 67.8% viability) as compared to PEI 25 kDa (12.7% viability).	[98]
pDNA	Tetraethylenepentamin (TEPA)	β-cyclodextrin (β-CD)	TEPA-β-CD nanoparticles	~350 nm at 1.71 N/P ratio	TEPA-BCD-based polyplexes were efficiently internalized by pigment epithelial cell line and a mouse embryonic fibroblast cell line (3T3) through clathrin- and caveolae-mediated endocytosis. TEPA-β-CD/pDNA showed 97% transfection efficiency while maintaining 83% cell viability.	[99]
***** EGFP-mRNA, ****** OVA-mRNA	Quaternary ammonium	β-cyclodextrin	Hyper-branched cyclodextrin-based polymer nanoparticles (Ppoly)	201, 167, 301 nm at 1:1, 1:5, 1:10 N/P ratio	77%, 72%, and 26% EGFP-mRNA was taken up by B16–F10 cells at 1:10, 1:5, and 1:1 N/P ratios of Ppoly while 58% lipofectamine-treated EGFP-mRNA was internalized at a 3:1 N/P ratio. As compared to the untreated group, OVA-mRNA exhibited three times higher tumor suppression by triggering a robust adaptive immune response.	[100]
siRNA	Spermidine amino acid	β-cyclodextrin (β-CD)	β-CD/adamantane-poly (vinyl alcohol)-poly (ethylene glycol) (β-CD/Ad-PVA-PEG) complexes	132 nm at 2:1 N/P ratio	The optimized β-CD/Ad-PVA-PEG complexes showed 90.5% siRNA binding efficiency. The complexes showed almost equivalent siRNA uptake to A549 cells and cell viability (67% vs. 65%) to the standard lipofectamine.	[101]
siRNA	Polyethylenimine 600 (PEI 600)	β-cyclodextrin (β-CD)	Polyethylene glycol–chitosan-grafted-PEI-β-CD copolymers (PEG-CT-PEI-β-CD)	80–150 nm at N/P ratio of 8	Standard PEI 25 kDa/siRNA showed 20–30% luciferase knockdown while CT-PEI-β-CD/siRNA resulted in 60% knockdown in L929 cells. PEGlyation of these carriers resulted in 84% luciferase knockdown, which was comparable to that of commercial DharmaFECT.	[102]

Abbreviations: * N/P ratio—nitrogen/phosphate ratio; ** LPEI22k—linear polyethylenimine with 22000 molecular weight; *** GFP—green fluorescent protein; **** PEI—polyethylenimine; ***** EGFP—enhanced green fluorescent protein; ****** OVA—oval albumin.

## Supplementary Materials

The following supporting information can be downloaded at: https://www.mdpi.com/article/10.3390/pharmaceutics17010081/s1, Table S1: Development of cationic cyclodextrin-based systems for anticancer drug delivery, Table S2: Development of cationic cyclodextrin-based systems for anti-inflammatory drug delivery, Table S3: Development of cationic cyclodextrin-based systems for antibacterial drug delivery, Table S4: Development of cationic cyclodextrin-based delivery systems for antidiabetic drugs, Table S5: Development of cationic cyclodextrin-based delivery systems for miscellaneous drugs, Table S6; Development of cationic cyclodextrin-based systems for nucleic acid delivery, Table S7: Development of cationic cyclodextrin-based systems for combinatorial delivery of drugs and nucleic acids, Table S8: Categorization of drug/nucleic acid delivery systems based on types of positively cyclodextrins. Note: Tables S1, S2, S3 and S6 are same as Table 1, Table 2, Table 3 and Table 4 included in this manuscript however, these tables also compile the examples discussed in their respective sections.
