# Supplementary material for: Cationic Cyclodextrin-Based Carriers for Drug and Nucleic Acid Delivery
Source: Pharmaceutics. 2025 Jan 9;17(1):81. doi: 10.3390/pharmaceutics17010081 (PMC11768558; doi:10.3390/pharmaceutics17010081)
Supplement: Supplementary file 1 [file pharmaceutics-17-00081-s001.zip › pharmaceutics-3346071-supplementary.pdf]

Table S1. Development of cationic cyclodextrin-based systems for anticancer drug delivery.

Drug	Cationic motif	Type of cyclodextrin	Type of formulation	Outcomes	References
Doxorubicin (DOX) + Celastrol (CSL)	Mono-(6-pentaethylenehexaamine) (PEHA)	β-cyclodextrin (β-CD)	Nanoparticles (NPs)	The DOX(CSL)-loaded PEHA-β-CD NPs efficiently mediated the apoptosis of colon cancer cells (SW480) and liver cancer cells (SMMC-7721). Moreover, NPs showed lower toxicity to (IC_{50} 0.57 μg/mL) normal epithelial cells (BEAS-2B) as compared to free DOX (IC_{50} 0.21 μg/mL)	[1]
Doxorubicin (DOX)	Poly (2-(dimethylamino) ethyl methacrylate) (PDMAEMA)	β-cyclodextrin (β-CD)	Star polymer	The DOX-loaded star polymers inhibited tumor growth at a higher rate (62.4%) than free DOX (37.2%) in xenograft mice bearing human cervical cell lines.	[2, 3]
Doxorubicin (DOX)	Poly(ß-amino ester)	β-cyclodextrin (β-CD)	Nanoparticles (NPs)	The NPs showed 100% and 60% higher permeability coefficients than that of the dextran control across monolayers of bovine brain microvascular endothelial cells (BBMVECs) and human brain microvascular endothelial cells (HBMVECs).	[4]
Doxorubicin (DOX)	Polyethyleneimine (PEI)	β-cyclodextrin (β-CD)	Ternary conjugates of bisphosphonate and PEI β-CD	In vitro study demonstrated the mitochondrial targeting and potential to induce a targeted drug delivery to different bone-related cancer cells i.e., MG-63 and MDA-MB-231 resulting in superior cytotoxicity. The in vivo evaluation also confirmed the targeting of these conjugates in xenograft mice bearing MG-63 and MDA-MB-23 cells.	[5]
Methotrexate (MTX)	Imidazole and quarternary ammonium	β-cyclodextrin (β-CD)	Nanoparticles (NPs)	MTX-loaded NPs showed relatively higher cytotoxicity (IC_{50} 7.22 μg/mL) than free MTX (IC_{50} 7.82 μg/mL) towards Saos-2 bone cancer cells. Moreover, flow cytometry cell analysis revealed that 9.21% of the MTX-loaded NPs were taken up by the Saos-2 cells during 1 hour	[6]
Methotrexate (MTX)	Poly (2-(dimethylamino) ethyl methacrylate) (PDMAEMA)	β-cyclodextrin	Hydrogels	Hydrogels showed higher cytotoxicity (IC_{50} 27 μg/mL) towards MCF-7 cells than free MTX (IC_{50} 55 μg/mL) and showed pH sensitive drug release profile (32.56% at pH 7.4 vs 85.3% at pH 5.2).	[7]

Paclitaxel (PTX)	Ammonium chloride	β-cyclodextrin (β-CD)	Nanoparticles (NPs)	The NPs reduced the viability of MCF-cell lines to a greater extent 30.7% than NPs based on non-ionic β-CD derivative 51.7%.	[8]
Doxorubicin (DOX)	Quaternary ammonium	β-cyclodextrin	Nanoparticles (NPs)	Cationic cyclodextrin NPs improved the permeability of DOX across *BBMVEC monolayer up to 2.2-fold. NPs were less cytotoxic to BBMVEC monolayer than free DOX. Moreover, NPs killed U87 tumor cells as effectively as bare DOX.	[9]
4-hydroxy-tamoxifen (TMX)	Quaternary ammonium	β-cyclodextrin	Cationic poly(cyclodextrin)/ alginate nanocapsules	In vitro study demonstrated that nanocapsules can efficiently deliver the TMX to immortalized mouse podocyte cells which mediates Cmip exon 8 deletion.	[10]
5-Fluorouracil (5-FU)	Quaternary ammonium	β-cyclodextrin	Cationic-β-CD loaded alginate/chitosan nanoflowers	The nanoformulation showed a pH-dependent and sustained 5-FU release profile up to 24 h. Further, in vivo studies are still in progress to evaluate the effectiveness of these nanoflowers in animal models.	[11]
Camptothecin (CPT)	Quaternary ammonium	Polycationic ß-cyclodextrin derivative (PC ßCDC6)	Nanoparticles (NPs)	NPs showed a promising mucus layer penetration rate (73%) and released a smaller CPT content (48%) in **SGF and ***SIF, hence, demonstrating the potential for colon-targeted drug delivery. Moreover, nanoformulation reduced the viability of HT-29 cells (52.44%) to a greater extent than free CPT solution (83.98%) at an equivalent concentration (0.1 μg/mL)	[12]
Melphalan (MLP)	Gemini surfactant	β-cyclodextrin	Inclusion complexes	MLP complexes showed a significantly reduced IC_{50} than free MLP (27 μM vs 98 μM) against A375 cell lines. Hence, the inclusion of MLP with cationic-cyclodextrin can improve its efficacy.	[13]
Scutellarin (SCU)	Poly(ε-lysine) (ε-PL)	β-cyclodextrin (β-CD)	ε-PL and glycine β-CD (ε-PL-GLY-CD) based inclusion complexes	ε-PL-GLY-C exhibited improved aqueous solubility of SCU (52.82 mg/mL) than that of bare SCU (0.16 mg/mL). Moreover, these inclusion complexes exhibited higher cytotoxicity against HCT116 and LOVO cells (IC_{50} 8.2 and 19.4 μM) as compared to bare SCU (IC_{50} 72.3 and 80.6 μM), respectively.	[14]
Scutellarin (SCU)	Triethylenetetramine	β-cyclodextrin	Inclusion complexes	Complexes showed improved water solubility (50.7 mg/mL) than that of free SCU (0.16 mg/mL). Furthermore,	[15]

				the cytotoxic potential of these inclusion complexes was remarkably higher towards HCT116 and LOVO cells (IC_{50} 0.9, 8.3 μM) than that of free SCU (IC_{50} 72.3, 80.6 μM), respectively.	

Abbreviations: * BBMVEC; bovine brain microvascular endothelial cell, **SGF; simulated gastric fluid, ***SIF; simulated intestinal fluid.

Table S2. Development of cationic cyclodextrin-based systems for anti-inflammatory drug delivery.

Drug	Cationic motif	Type of cyclodextrin	Type of formulation	Outcomes	References
Indomethacin (IDM)	Chitosan	β-cyclodextrin (β-CD)	Nanoparticles (NPs)	The hydrogel attained 90% swelling at pH 1.4 and 60% swelling at pH 7.4, within 6 h. Consequently, a higher drug release rate was noticed at pH 1.4 (~85%) than that at pH 7.4 (~57%). Hence, this cationic-cyclodextrin-based formulation can be employed for targeted and controlled drug delivery.	[16]
Indomethacin (IDM)	Chitosan (CS)	β-cyclodextrin	Electrospun nanofibers	Nanofibers showed a sustained release of IDM and equilibrium was attained within 125 h while ~80% payload was liberated within 300 h. Moreover, nanofibers were biocompatible as the growth of L929 cells was not inhibited to a significant extent.	[17]
Indomethacin (IDM)	Quaternary ammonium	β-cyclodextrin	Hydrogel	The solubility of IDM (0.0615 mmol/L) was 100-fold enhanced after complexation with cationic β-CD (6.5 mmol/L). In simulated gastrointestinal fluid, the hydrogel was completely swollen within 14-16 h and 100% of IDM was released within 21 h. Hence, these hydrogels can serve as a controlled release system for IDM.	[18]
Dexamethasone (DXM)	Quaternary ammonium chitosan (QA-Ch60)	Methyl-β-cyclodextrin (MCD)	Conjugates	The conjugates formed a stable complex with DXM while improving its aqueous solubility and retained significant mucoadhesion. Moreover, conjugate-treated RCE cell lines showed high viability (80%) hence, confirming their biocompatibility.	[19]
Dexamethasone (DXM)	(6-aminohexyl) amino	β-cyclodextrin (β-CD)	Gellan gum complex hydrogel	The cell-cultured Dx@HCD-GG hydrogel showed the highest glycosaminoglycan (GAGs) and double-stranded	[20]

				DNA (dsDNA) contents indicating the enhanced chondroprotective effect of DXM. During the in vivo study (cartilage defect model), the hydrogel-treated group showed highly dense regenerated tissues which were well interacted with the surrounding tissues. Moreover, the formation of glycosaminoglycan matrix was also noticed, hence confirming the cartilage regeneration potential of Dx@HCD-GG hydrogel.	
Dexamethasone (DXM)	Poly (2-(dimethylamino) ethyl methacrylate) (PDMAEMA)	β-cyclodextrin (β-CD)	β-CD-graft-(poly(ε caprolactone)-block- PDMAEMA (β-CD-g-(PCL-PDMAEMA) star-like amphiphilic polymer-based inclusion complex	β-CD-g-(PCL-b-PDMAEMA-loaded DXM efficiently reduced the lipopolysaccharide-mediated release of cytokines (interleukin-1β, interleukin-6, and interleukin-10) in RAW264.7 macrophages than that of free DXM.	[21]
Naproxen (NPX)	Quaternary ammonium	β-cyclodextrin (β-CD)	Inclusion complexes	The cationic β-CD complexation improved the aqueous solubility of NPX up to 120-fold. Conjugates exhibited a faster dissolution rate and higher amount of drug dissolution. Moreover, cationic β-CD exhibited relatively low hemolytic activity compared with parent β-CD hence, confirming the biocompatibility of these drug carriers.	[22]
Ketoprofen (KTP)	Chitosan (CS)	β-cyclodextrin (β-CD)	Nanoparticles (NPs)	CD-g-CS NPs exhibited a sustained release profile at pH 6.8 than that of chitosan NPs and KTP release could be further slowed with increasing substitution degree of cyclodextrin thus reducing the need for frequent dosing of KTP.	[23]
Ketoprofen (KTP)	Chitosan	β-cyclodextrin	Nanoparticles (NPs)	NPs exhibited a sustained KTP release profile in *PBS (at pH 7.4) and attained the equilibrium after 23 h while bare chitosan nanoparticles liberated the entire payload within 9 h. Moreover, NPs were considered biocompatible based on the high viability of L929 cells.	[24]
Diclofenac sodium (DCF)	Amino groups	Poly-β-amino-cyclodextrin derivative	Nanoassemblies	PolyCD-based nanoassemblies were efficiently taken up by **hMSCs (within 2 h) without imparting any cytotoxic impact. Subsequently, interleukin-1β and tumor necrosis factor-α levels were reduced to a significant extent as compared to other treatment groups. Moreover, DCF was released in a controlled manner (~35% within 10 days).	[25]

Ibuprofen (IBU)	K^+ cations	β-cyclodextrin	Metal-organic frameworks (MOFs)	The aqueous solubility of IBU entrapped in MOFs was improved up to 17-fold.	[26]
Niflumic acid (NIF)	K^+ cations	γ-cyclodextrin	Metal-organic frameworks (MOFs)	MOFs showed a pH-dependent drug release i.e., 14% NIF in 1 h and 40% NIF in 6 h at pH 1.6 while 40% NIF in 2 h and the remaining amount was completely released in 8 h. NIF incorporated in the frameworks also showed improved solubility.	[27]
Ketotifen hydrogenfumarate (KHF)	***HHDDP surfactant	γ-cyclodextrin (γ-CD)	Hyaluronic acid (HA)/γ-CD/HHDDP polymer assemblies	The water-insoluble polymer matrix released KHF (water-soluble drug) slowly while free KHF was completely dissolved in a physiological saline solution over 35 min. Hence, this delivery system can be employed to attain a sustained release profile of water-soluble drugs.	[28]
Meloxicam (MLX)	Triethanolamine (TEA)	β-cyclodextrin (β-CD)	Conjugates	The resultant MLX-β-CD-TEA conjugate showed significantly improved dissolution with ~85% cumulative drug dissolved than that of pure MLX (~30%). MLX-β-CD-TEA ternary complexes showed higher edema inhibition (84.38%) as compared to pure MLX (59.37%).	[29]
Budesonide (BUD)	Benzalkonium chloride	β-cyclodextrin (β-CD)	Hydrogel	The resultant hydrogel film showed improved dissolution (87.2% vs 63%) and mucosal permeation (95.8% vs 40.2%) as compared to film without cyclodextrin and benzalkonium. The topical application of the formulation to the rabbit eye was capable of reducing the symptoms of inflammation (redness) within 3 h.	[30]

Abbreviations: *PBS; phosphate buffer saline, **hMSCs; human mesenchymal stromal cells, ***HHDDP; [hexadecyl(2-hydroxyethyl)dimethylammonium dihydrogen phosphate.

Table S3. Development of cationic cyclodextrin-based systems for antibacterial drug delivery.

Drug	Cationic motif	Type of cyclodextrin	Type of formulation	Outcomes	References
Ciprofloxacin (CFX)	Quaternized chitosan	Not-specified	Nanoparticles (NPs)	The nanoformulation initially released the CFX at a higher rate (~35% within 0.5-1 h) due to the desorption of the surface-adsorbed drug. Subsequently, CFX was released in a sustained manner (~90% within 24 h) which was mediated by CFX diffusion through the NPs matrix and erosion of the polymeric matrix. Moreover, nanoformulation showed a highly potent antibacterial activity for both Staphylococcus aureus and Escherichia coli (MIC 6.25 µg/mL).	[31]
Ciprofloxacin (CFX)	Polyamidoamine	β-cyclodextrin (β-CD)	Nanoparticles (NPs) on polyester fabric	The resultant fabric showed higher drug absorption (16.9% vs 3.7% after 24 h) and sustained drug release (45% vs 92% after 3 h) as compared to raw fabric. Modified fabric showed 100% antibacterial efficiency for both *E.coli* and *S. aureus*. Moreover, the fabric didn't impart any cytotoxic impact on fibroblast cells, hence confirming its biocompatibility.	[32]
Vancomycin (VCM)	Oleylamine (OLA)	β-cyclodextrin (β-CD)	Cationic amphiphile derivative	βCD-OLA released the VCM in a sustained manner i.e., ~65% and ~80% after 24 h and 48 h, respectively. Furthermore, BCD-OLA/VCM showed a 4-fold reduced MIC (7.81 µg/mL) towards Methicillin-resistant *S. aureus* as compared to free vancomycin (31.25 µg/mL). BCD-OLA/VCM caused the 459-fold reduction of intracellular bacteria using infected human embryonic kidney cells (HEK), and an 8-fold reduction in infected macrophages as compared to free vancomycin.	[33]
Silver sulfadiazine (SSD)	Chitosan	Not specified	Supramolecular polyelectrolyte complexes (SPEC)	The SSD/SPEC showed smaller zones of inhibitions than that of free SSD i.e., 18 vs 25 mm, 18 vs 20 mm, 17 vs 23 mm for *S.aureus*, *Klebsiella pneumoniae*, and *E. coli*. The improved antibacterial activity might be attributed to the electrostatic interactions between positively charged amine groups of chitosan with the negatively charged bacterial cell wall components.	[34]

Oxacillin (OXA)	Aminoethylthio	γ-cyclodextrin (γ-CD)	Complex	It was demonstrated that γ-CD complex resulted in a 2.3-fold reduction of β-lactamase induced OXA hydrolysis. Moreover, γ-CD complex was efficiently internalized by macrophages i.e., 25% internalization in the first 15 minutes while 99.8% internalization within 24 h. Moreover, γ-CD complex was found as biocompatible based on the viability of L929 cells.	[35]
Rifampicin (RFP)	Aminoethylthio	γ-cyclodextrin (γ-CD)	Complex	Drug-loaded RFP/γCys complex reduced biofilm viability to the background levels (~100%) while Free RFP showed moderated anti-biofilm activity (~60% reduction). The remarkable anti-biofilm potential of γCys/RFP might be attributed to the improved solubility of RFP upon complexation and/or synergistic interference with components of the biofilm.	[36]
Enrofloxacin (ENF)	Potassium ions	γ-cyclodextrin (γ-CD)	Metal-organic frameworks (MOF)	The γ-CD-MOF released 40% of ENF in 1 h and 87.5% of ENF in 4 h. The γ-CD-MOF/NF showed ~100% bacterial inhibition in 6 min and retained ~95% bacterial inhibition till 24 min. However, free ENF exhibited ~85% bacterial inhibition in 6 min while ~70% inhibition in 24 min. Hence, γ-CD-MOF/NF exhibited a higher and longer bacterial growth inhibition potential as compared to free ENF.	[37]
Sulfadiazine (SSD) Sulfamonomethoxine (SMMX) Sulfamethoxazole (SMZ)	Chitosan (CS)	β-cyclodextrin (β-CD)	Inclusion complexes based on CD-grafted with β-CD (CD-g-CS)	The aqueous solubilities of free SSD, SMMX, and SMZ were noticed as 0.026, 0.069, and 0.329 mg/mL, respectively. However, CD-g-CS-based SSD, SMMX, and SMZ complexes showed significantly improved solubilities up to 5.6-fold (0.147 mg/mL), 4.1-fold (0.283 mg/mL), and 2.3-fold (0.747 mg/mL), respectively.	[38]
Levofloxacin (LVX)	Chitosan (CS)	β-cyclodextrin (β-CD)	CS-β-CD inclusion complexes	Simple β-CD conjugates released 100% LVX in 180 min while CS-β-CD complexes released the complete payload in 800 h. Hence, CS-β-CD complexation provided a 4-fold slow drug release in comparison to bare β-CD. These complexes can serve as sustained drug delivery systems.	[39]
Linezolid (LZD)	Amino groups	β-cyclodextrin	Au@Ag bimetallic	MIC values of LZD loaded NPs and free LZD were noticed as 2 vs 1.95 µg/mL for *MRSA, 2 vs 0.97 for *S.aureus*, 2 vs 15.6	[40]

			nanoparticles (BMNPs)	μg/mL for *E. coli*, 3.9 vs 15.6 μg/mL for *P. aeruginosa*. Hence, LZD-nanoformulation showed a broad spectrum of activity than free LZD.	
Triclosan (TR)	Quarterny ammonium	β-cyclodextrin	Inclusion complexes	The water solubility of TR increased linearly until the solubility limit was achieved i.e., 1.4 mg/mL TR with 3.8 mg/mL cationic β-CD. Moreover, cationic β-cyclodextrin polymers were considered biocompatible due to the high viability (99%) of human colon carcinoma Caco-2 cells.	[41]
Triclosan (TR) and Butylparaben (BP)	Quarterny ammonium	β-cyclodextrin (β-CD)	Inclusion complexes	The *E. coli* growth inhibition potential of TR/ β-CD was higher than that of BP/β-CD at lower than 0.5% concentration while above 0.5% concentration both antibiotics exhibited 100% inhibition of bacterial growth. Hence, TR/β-CD complex was more potent than BP/(β-CD). However, BP complex showed faster growth inhibition rated than TR complex in a short contact time (10 minutes).	[42]
Nitric oxide (NO)	Poly(amidoamine) dendron (PAMAM)	β-cyclodextrin (β-CD)	β-CD-PAMAM/NO inclusion complexes	β-CD-PAMAM/NO complexes (50 μg/mL), showed 80% and 94% biofilm inhibition for *E. coli* and *S. aureus* within 18 h. Moreover, β-CD-PAMAM/NO exhibited excellent biocompatibility and didn't impart any cytotoxic impact on NIH 3T3 cells.	[43]
α-mangostin (α-MG)	Quaternary ammonium and chitosan	β-cyclodextrin (β-CD)	Quaternized β-CD grafted-chitosan (QCD-g-CS) based inclusion complexes	QCD-g-CS/α-MG complexes showed an initial burst release of α-MG (up to 5 h) followed by sustained release (up to 24 h). QCD-g-CS/α-MG showed significantly lower MIC i.e., 0.6 and 1.25 mg/mL for *Streptococcus mutans* and *Candida albicans* while bare α-MG showed >10 mg/mL MIC for both microbes.	[44]

Abbreviations: *MRSA, methicillin-resistant *Staphylococcus aureus*.

Table S4. Development of cationic cyclodextrin-based delivery systems for antidiabetic drugs.

Drug	Cationic motif	Type of cyclodextrin	Type of formulation	Outcomes	References
Metformin hydrochloride (MTF)	Diaminodipropylamine	β-cyclodextrine (β-CD)	Nanoparticles (NPs)	Drug-loaded NPs released a significantly lower amount (7.55%) of MTF in a simulated gastric fluid (pH 2) than that of MTF released from solution form (13%). Moreover, NPs released MTF at higher rates with increasing pH values as compared to the drug solution i.e., 33.68% vs 17% at pH 6.3 and 61.98% vs 27% at pH 8. Hence, these NPs can reduce the degradation of MTF in the stomach and promote its absorption in the small intestine	[45]
Liraglutide (LTD)	Propyl-amine	Not specified	Nanoparticles (NPs)	The nanoformulation could prevent the degradation of LTD upon incubation with simulated intestinal fluid supplemented with enzymes (up to 4 h) while the LTD solution rapidly underwent degradation (within 5 min). Followed by intestinal administration, LTD-NPs reduced the glucose load (71%.hr) which was quite close to the hypoglycemic effect (68%.hr) of subcutaneous LTD solution.	[46]
Insulin	Polyethylenimine (PEI)	β-cyclodextrine (β-CD)	Polymers	Hydroxypropyl-β-CD-PEI1800 exhibited the highest insulin absorption enhancing efficiency followed by hydroxypropyl-β-CD-PEI10000 and hydroxypropyl-β-CD-PEI600 with pharmacological availability of 17.40%, 14.03%, and 11.46%, respectively. Hence, the degree of positive charge was linearly correlated with the absorption-enhancing effect of hydroxypropyl-β-CD-PEI.	[47]
Insulin	Diethylenetriamine (DETA)	β-cyclodextrine (β-CD)	Poly(glycidyl methacrylate)s (PGOHMAs) based polyelectrolyte complexes (PECs)	The cumulative release of insulin from CD-series complexes (~80-95%) was higher than that of D-series complexes (~57-67%). The CD-series showed less toxicity towards L929 cells (nearly 100% viability) than the D-series (~10-70% viability) hence, the introduction	[48]

				of cyclodextrin mitigated the toxicity of amino PGOHMA by decreasing the density of amino groups.	
Insulin	Quaternary ammonium	β-cyclodextrine (β-CD)	alginate/chitosan nanoparticles (NPs)	Simple alginate/chitosan NPs released up to 60% insulin in simulated gastric fluid (pH 1.2) while only 18% of insulin was released in simulated intestinal fluid (pH 6.8). Hence, most of the payload was lost before it could reach its target site (intestine) to show any therapeutic effect. The optimized CPβCDs-insulin-loaded alginate/chitosan NPs released 48% of insulin in simulated gastric fluid while 40% of insulin was successfully liberated in simulated intestinal fluid.	[49]

Table S5. Development of cationic cyclodextrin-based delivery systems for miscellaneous drugs.

Drug	Cationic motif	Type of cyclodextrin	Type of formulation	Outcomes	References
Vitamin B_2	Guanidine	β-cyclodextrin	Conjugates	The aqueous solubility of vitamin B_2 conjugates (673 g/L) was significantly higher than that of bare vitamin B2 (0.078 g/L). Moreover, conjugates released the payload in a sustained manner i.e., 89.1%, 71.5%, and 91.7% at pH 10, 7.4, and 1.2 after 23 h.	[50]
Heparin	Quaternary ammonium	β-cyclodextrin	Self-nano-emulsifying drug delivery system (SNEDDS	The cumulative amount of heparin liberated from SNEDDS after 120 min incubation in SGF (pH 1.2) followed by further 240 min incubation in SIF (pH 6.8) was 47.31%. Although,100% of the payload was not released while it was referred that considering the physiological emptying time in gastrointestinal tract, studying the in vitro release profile for 6 h is needed for the prediction of system behavior under in vivo.	[51]
Rebamipide (REB)	*N,N,N*-trimethyl-N-(2-hydroxy-3-metacryloyloxopropyl)-ammonium chloride (QA)	β-cyclodextrin (β-CD)	Conjugates (CDQA)	The solubility of rebamipide was enhanced to a significant extent in CDQA solution (10.27 μM concentration) as compared to that in β-cyclodextrin solution (3.8 μM concentration). Furthermore, the CDQA solution promoted rebamipide penetration	[52]

				across the cornea. The instillation of REB@CDQA resulted in higher lacrimal fluid volume (1.3-fold) and mucin levels (1.5-fold) as compared to the control (rebamipide suspension) along with significant attenuation of tear film breakup.	
Daidzein (DAI)	Glycidyltrimethylammonium chloride (GTMAC) and ethylenediamine (EDA)	γ-cyclodextrin (GCD)	Conjugates	At an equivalent concentration (100 μg/ml) of GCD-GTMAC/DAI and GCD-EDA/DAI, levels of the cellular glycosaminoglycans were dropped down to 72% and 62% of the control, respectively. It was suggested that such cationic GCD derivatives-based daidzein inclusion complexes may be employed to reduce the accumulation of glycosaminoglycans in mucopolysaccharidoses and lysosomal storage diseases.	[53]

Table S6. Development of cationic cyclodextrin-based systems for nucleic acid delivery.

Nucleic acid	Cationic motif	Type of cyclodextrin	Description of vector	Size of polyplexes	Outcomes	References
pDNA	Oligoethylenimine (OEI)	α-cyclodextrin (α-CD)	Cationic star polymer (α-CD-OEI) based on grafting of OEI arms onto an α-CD core	200 nm at 5 *N/P ratio	The star polymer exhibited 50-fold higher pDNA transfection efficiency in HEK293 cells than that of PEI25 KDa in a serum-free environment however, both agents showed comparable transfection efficiencies under serum conditions. Moreover, α-CD-OEI was less cytotoxic to HEK293 cells (~35% viability) as compared to PEI25 KDa (~5% viability).	[54]
pDNA (luciferase plasmid (pCMV-Luc))	Primary, tertiary, and quaternary ammonium groups	β-cyclodextrin	Cationic star polymers with 21 arms (21ACSPs)	80-180 nm	21ACSPs with primary and tertiary amino groups showed satisfactory transfection efficiency to CHSE-214 cells i.e., ~23 and ~28 ng luciferase/mg protein. However, quaternary ammonium containing 21ACP was unable to show any transfection efficiency as the quaternary ammonium cannot be further protonated in the acidic environment of the endosome. Moreover,	[55]

					transfection with 21ACSP/pDNA polyplexes showed high viability of CHSE-214 cells (77-88%) thus, confirming their biocompatibility.	
pDNA	Polyethyleneimine (PEI)	β-cyclodextrin (β-CD)	Supramolecular systems based on coupling of adamantane-grafted PEI with L-cystine-bridged bis(β-CD) (PEI-Ada-LCD)	260-70 nm size at 10-40 N/P ratios	The optimized PEI-Ada-LCD formulation showed almost 2-fold higher gene transfection efficiency (54%) than that of the gold standard i.e., PEI25 KDa (~32%) in 293T cells. Moreover, PEI-Ada-LCD was less cytotoxic toward 293T cells as compared to PEI25 KDa.	[56]
pDNA	Polyethyleneimine 600 (PPC)	β-cyclodextrin (β-CD)	Epsilon-polylysine-grafted-succinic acid-grafted-β-CD- PPC entrapped with adamantane-functionalized poly-(ethylene glycol) derivative (PEG-AD)	200 nm at 20 N/P	The pDNA transfection efficiency of PPC/PEG-AD was almost similar to PEI25 KDa. However, PPC/PEG-AD showed extremely lower cytotoxicity to HEK293 cells (~95% viability) as compared to PEI25 KDa (~10% viability) at an equivalent concentration.	[57]
pDNA (luciferase-encoding plasmid DNA, pCMVLuc)	Amino groups	β-cyclodextrin (β-CD) derivative T2	Polypexes grafted with folic acid (fol-CDplexes)	263 nm at 5 N/P ratio	The fol-CDplexes showed significantly higher gene transfection efficiency than that of plain-CDplexes and standard PE125 KDa polyplexes in HeLa cells i.e., ~68, ~38, ~18 ng luciferase/mg protein, respectively. Further in vivo study demonstrated that gene expression induced by Fol-CDplexes was 2-fold and 4.6-fold higher in liver and lung tissues, respectively as compared to plain-CDplexes	[58]
pDNA	Poly(2-(dimethylamino) ethyl methacrylate (PDMAEMA)	β-cyclodextrin (β-CD)	Star-like amphiphilic β-CD-graft-(poly(ε-caprolactone)-block(PDMAEMA)x (β-CD-g-(PCL-b-PDMAEMA)x) copolymer	220 nm at 0.5 N/P ratio	β-CD-g-(PCL-b-PDMAEMA)x showed significantly higher pDNA transfection efficiency in RAW264.7 macrophages (10.8%) than that of standard lipofetamine (2.6%). Moreover, the copolymer showed low cytotoxicity (IC50 40 μg/mL) hence, confirming its biocompatibility	[21]

pDNA	Oligoethylenimine (OEI)	γ-cyclodextrin (γ-CD)	Sar-shaped polymers grafted with folic acid through disulfide bonds (γ-CD-OEI-SS-FA)	100 to 150 nm at 2.5 N/P ratio	The optimized γ-CDOEI-SS-FA formulation showed a 6-fold higher gene transfection efficiency in KB cells as compared to standard PEI25 KDa while imparting relatively less cytotoxic impacts.	[59]
pDNA	Polyethylenimine 600 Da (PEI)	Hydroxypropyl-γ-cyclodextrin (HP-γ-CD)	Polymeric carriers grafted with MC-10 oligopeptide (HP-γ-CD-PEI-P)	175 nm at 40 N/P ratio	The HP-γ-CD-PEI-P showed 4 and 3.7-fold higher pDNA transfection efficiency to SKOV-3 cells than that of PEI25 KDa and non-functionalized HPg-CD-PEI, respectively. Moreover, HP-γ-CD-PEI-P didn't show any noticeable cytotoxicity towards SKOV-3 cells up to 120 N/P ratio while standard PEI25 KDa showed less than 20% cell viability at 40 N/P ratio.	[60]
pDNA	Dimethylaminoet hyl (DMAE)	α-cyclodextrin (α-CD)	Polyrotaxanes based on cationic α-CD and disulfide-grafted poly(ethylene glycol) (PEG)	178-189 nm at 0.5 N/P ratio	The polyrotaxanes formed stable polyplexes at 0.5 N/P ratio with positive zeta potential (+4.8 mV) while **LPEI22k was unable to form compact polyplex and showed negative zeta potential. Moreover, polyrotaxane exhibited concentration-dependent transfection efficiency and precluded lysosomal degradation of pDNA due to the proton sponge effect.	[61]
pDNA	Oligoethylenimine (OEI)	α-cyclodextrin (α-CD)	Fe_3O_4 nanoparticles functionalized with α-CD-OEI	120 nm at 20 N/P ratio	The implication of magnetic field improved the gene transfection efficiency of nanocarriers up to 10-fold. Magnetic field mediated the accumulation of polyplexes on the cell membrane and facilitated their penetration into the cell.	[62]
miR-34a	Polyethyleneimine (PEI)	β-cyclodextrin (β-CD)	β-CD-PEI cationic polymers grafted with polyethylene glycol and matrix metalloproteinase (MMP)-2 cleavable peptide (CD-PEI-C-PEG)	99.17 at 4 N/P ratio	During the in vivo study, CD-PEI-C-PEG polyplexes exhibited significantly higher tumor inhibition potential (51.79%) than that of CD-PEI (7.01%).	[63]
miR-34a-5p	Polyethyleneimine (PEI)	β-cyclodextrin (β-CD)	Conjugates grafted folic acid (β-CD-PEI-FA)	203.13 at 5 N/P ratio	As compared to the control, β-CD-PEI-FA showed 306.12 and 2.73 times higher miR-34a-5p expression in BCBL-1 and SK-RG cells. Moreover, β-CD-PEIFA showed up to 70% viability of BCBL-1 and SK-RG cells, hence confirming their biocompatibility.	[64]

siRNA	Lysine amino acid	β-cyclodextrin (β-CD)	Conjugates	288.9 at 10 N/P ratio	The resultant polyplexes were efficiently internalized by prostate cancer cells (DU145, VCaP, and PC3 cells) while maintaining > 80% cell viability. The nanoplexes exhibited efficient silencing of the PLK1 gene which is involved in numerous types of cancers. The carriers protected siRNA from nuclease digestion for up to 24 h while naked siRNA was partially degraded within 8 h.	[65]
siRNA	Guanidinium (GD)	β-cyclodextrin (β-CD)	GD and polyethylene glycol attached with primary and secodary surface of β-CD, respectively and conjugate grafted with anisamide (G-CD-PEG-AA)	~210-260 nm at 75 N/P ratio	G-CD-PEG-AA induced prostate cell-specific internalization of siRNA resulting in approximately 80% knockdown luciferase (reporter gene). Followed by intravenous administration, G-CD-PEG-AA/vascular endothelial growth factor (VEGF) siRNA exhibited a 3-times reduction in tumor volume as compared to phosphate buffer saline.	[66]
siRNA	Polyethyleneimine	2-hydroxypopyl-β-cyclodextrin (β-CD)	Conjugates functionalized with folic acid (FA-PEI-HP-β-CD)	250 nm at 24 N/P ratio	FA-PEI-HP-β-CD completely condensed the siRNA into 250 nm particles at 24 N/P ratio. FA-PEI-HP-β-CD/siRNA were internalized by folate receptor enriched HeLa cells to a greater extent (4-fold) than non-targeted PEI-HP-β-CD while maintaining 90% cell viability. FA-PEI-HP-β-CD protected up to 90% of siRNA from nuclease degradation within 12 h while naked siRNA was completely degraded during the specified period.	[67]
MMP-9-siRNA	Poly(amidoamine)	β-cyclodextrin (β-CD)	cationic star-shaped polymers consisting of	268-110 nm at 6-60 N/P ratio	β-CD-PAMAM and standard lipofectamine 2000 exhibited transfection efficiency of 98.78% and 64.89% while inducing 5.57% and 1.21% fibroblast cell death. Furthermore, MMP-9 expression was reduced by 68% and 80% for the cells treated with β-CD-PAMAM/MMP-9-siRNA and lipofectamine 2000/MMP-9-siRNA, respectively.	[68]

siRNA	*N,N′*-dimethylethylene diamine (DMEDA)	*α*-cyclodextrin (*α*-CD)	*α*-CD:poly(ethylene glycol) polyrotaxanes	150-200 nm at 5 N/P ratio	The cationic polyrotaxanes exhibited >10^2-fold lower cytotoxicity than the standard PEI25 KDa. Moreover, polyrotaxanes showed comparable gene silencing efficiencies (60-70% reduced ***GFP expression) to those of Lipofectamine 2000 and PEI25 KDa.	[69]
siRNA	Ionene	*α*-cyclodextrin + β-cyclodextrin	Polyrotaxanes	127 nm at 2 N/P ratio	Polyrotaxanes mediated the siRNA internalization into A549 cell line (10 times higher fluorescence intensity than that of original cells) while exhibiting 60% knockdown efficiency of luciferase gene.	[70]
DNA	Polyethyleneimine 600 (PEI 600)	β-cyclodextrin (β-CD)	Amphiphilic β-CD based vectors	116.6, 96.6, and 100.2 nm at N/P ratio of 5, 10, and 30	PEI600-β-CD showed better gene transfection efficiency (~110% vs ~80% relative to control) than that of PEI25 KDa in 10% serum while both carriers showed almost similar efficiency i.e., 100% in a serum-free environment. Moreover, PEI600-β-CD were less cytotoxic to 293T cells at all the tested concentrations (10-100 μg/mL) i.e., ~60-90% viability vs ~50-80%. Polyplexes were up internalized through caveolae-mediated endocytosis which could prevent the lysosomal degradation of entrapped genes.	[71]
pDNA	Poly(2-(dimethylamino) ethyl methacrylate (PDMAEMA)	β-cyclodextrin (β-CD)	β-CD grafted-poly(ε-caprolactone)-PDMAEMA copolymer-based vectors	218 nm at 2 N/P ratio	β-CD-based vectors showed significantly higher gene transfection efficiency than that of ****PEI 25 KDa i.e., 84% vs 52.9% in HEK293T cells and 23.6% vs 10.6% in HepG2 liver cancer cells. Moreover, β-CD based vectors were less cytotoxic to both cell lines (up to 67.8% viability) as compared to PEI25 KDa (12.7% viability).	[72]
pDNA	Tetraethylenepentamin (TEPA)	β-cyclodextrin (β-CD)	TEPA-β-CD nanoparticles	~350 nm at 1.71 N/P ratio	TEPA-BCD based polyplexes were efficiently internalized by pigment epithelial cell line and a mouse embryonic fibroblast cell line (3T3) through clathrin-and caveolae-mediated endocytosis. TEPA-β-CD/pDNA showed 97% transfection efficiency while maintaining 83% cell viability.	[73]

*****EGFP-mRNA, ******OVA-mRNA	Quaternary ammonium	β-cyclodextrin	Hyper-branched cyclodextrin-based polymer nanoparticles (Ppoly)	201, 167, 301 nm at 1:1, 1:5, 1:10 N/P ratio	77%, 72%, and 26% EGFP-mRNA was taken up by B16–F10 cells at 1:10, 1:5, and 1:1 N/P ratios of Ppoly while 58% lipofectamine treated EGFP-mRNA was internalized at 3:1 N/P ratio. As compared to the untreated group, OVA-mRNA exhibited three times higher tumor suppression by triggering a robust adaptive immune response.	[74]
siRNA	Spermidine amino acid	β-cyclodextrin (β-CD)	β-CD: adamantane - poly (vinyl alcohol)- poly (ethylene glycol) (β-CD: Ad-PVA-PEG) complexes	132 nm at 2:1 N/P ratio	The optimized β-CD: Ad-PVA-PEG complexes showed 90.5% siRNA binding efficiency. The complexes showed almost equivalent siRNA uptake to A549 cells and cell viability (67% vs 65%) to the standard lipofectamine.	[75]
siRNA	Polyethyleneimine 600 (PEI 600)	β-cyclodextrin (β-CD)	Polyethylene glycol-chitosan grafted-PEI- β-CD copolymers (PEG-CT-PEI-β-CD)	80-150 nm at N/P ratio of 8	Standard PEI25 KDa/siRNA showed 20-30% luciferase knock down while CT-PEI-β-CD/siRNA resulted in 60% knock down in L929 cells. PEGlyation of these carriers resulted in 84% luciferase knockdown which was comparable to that of commercial DharmaFECT.	[76]

Abbreviations: *N/P ratio; nitrogen/phosphate ratio, **LPEI22k ; linear polyethyleneimine with 22000 molecular weight, ***GFP; green fluorescent protein, ****PEI; polyethyleneimin, *****EGFP; enhanced green fluorescent protein, ******OVA, oval albumin.

Table S7. Development of cationic cyclodextrin-based systems for combinatorial delivery of drugs and nucleic acids.

Nucleic acid	Drug	Cationic motif	Type of cyclodextrin	Formulation	Size of polyplexes	Outcomes	Refrences
OligoRNA	Doxorubicin	Poly-L-lysine	β-cyclodextrin	Nanocomplexes grafted with hyaluronic acid (HA-NPs)	195.8 nm at 30 N/P ratio	HA-NPs showed higher toxicity to MHCC-97H cells (IC_{50} 6.58 μg/mL) than that of non-functionalized NPs (IC_{50} 11.26 μg/mL). HA-NPs were mostly distributed in the tumor tissues while non-functionalized NPs were distributed both in the liver and tumor tissues.	[77]

siRNA	Doxorubicin (DOX)	Polyethylenimine	β-cyclodextrin	Folic acid grafted-magnetic hollow mesoporous silica NPs (FA- MHMSNs)	178 nm at 20 N/P	Free DOX was unable to impart cytotoxic impact (IC_{50} >500 μg/mL) while FA-MHMSNs NPs showed remarkable cytotoxicity towards MCF-7/ADR cells (IC_{50} > 6.20 μg/mL) which was further increased under the influence of alternating magnetic field (IC_{50} > 4.42 μg/mL).	[78]
MMP-9 shRNA plasmid (pMMP-9)	Doxorubicin (DOX)	Oligoethylenimine	β-cyclodextrin	Conjugates	~220 nm at 20 N/P ratio	The resultant carriers exhibited > 50% transfection efficiency to MCF-7 cells while standard PEI 25KDa showed only 17% transfection efficiency. The tumor-bearing mice treated with these carriers showed only 21% tumor volume as compared to phosphate buffer saline.	[79]
pDNA	Methotrexate (MTX)	Poly(L-lysine) dendron (PLLD)	6-azido-β-cyclodextrin (β-CD)	Conjugates	~200 nm at 20 N/P ratio	The resultant carriers released MTX in a more sustained manner (~45%) than that of β-CD (~50%) and PLLD (~70%) within 25 h. CD-PLLD-MTX treated MCF-cells exhibited < 70% viability at 50 μg/mL concentration.	
MMP-9 siRNA plasmid (pMR3)	Docetaxel (DOC)	Poly(L-lysine) dendron (PLLD)	6-azido-β-cyclodextrin (β-CD)	Conjugates	125 nm at 20 N/P ratio	CD-PLLD/DOC/pMR3 showed greater apoptosis of HNE-1 cells (55.5%) as compared to CD-PLLD/DOC (13.8%) and CD-PLLD/pMR3 (40.7%). The greater antitumor potential of CD-PLLD/DOC/pMR3 was attributed to the fact that released DOC could induce DNA damage while pMR3 could mediate the down-regulation of protein expression.	[80]
siRNA	Methotrexate (MTX)	Poly(amidoamine) dendrons (PAMAM)	Per-6-azido-β-cyclodextrin	Star-shaped polymers	100-200 nm at 30 N/P ratio	The carriers showed better transfection efficiency to fibroblast cells than PAMAM dendrimers i.e., ~85% vs ~75% with serum and ~97% vs ~82% without serum.	

						The carriers released the DOX in a sustained manner for up to 10 h.	

Table S8. Categorization of drug/nucleic acid delivery systems based on types of positively cyclodextrins.

Payload	Cationic motif	Type of cyclodextrin	Type of formulation	Outcomes	References
**Polyethyleneimine**					
Doxorubicin (DOX)	Polyethyleneimine (PEI)	β-cyclodextrin (β-CD)	Ternary conjugates of bisphosphonate and PEI β-CD	In vitro study demonstrated the mitochondrial targeting and potential to induce a targeted drug delivery to different bone-related cancer cells i.e., MG-63 and MDA-MB-231 resulting in superior cytotoxicity. The in vivo evaluation also confirmed the targeting of these conjugates in xenograft mice bearing MG-63 and MDA-MB-23 cells.	[5]
Insulin	Polyethylenimine (PEI)	β-cyclodextrine (β-CD)	Polymers	Hydroxypropyl-β-CD-PEI1800 exhibited the highest insulin absorption enhancing efficiency followed by hydroxypropyl-β-CD-PEI10000 and hydroxypropyl-β-CD-PEI600 with pharmacological availability of 17.40%, 14.03%, and 11.46%, respectively. Hence, the degree of positive charge was linearly correlated with the absorption-enhancing effect of hydroxypropyl-β-CD-PEI.	[47]
pDNA	Polyethyleneimine (PEI)	β-cyclodextrin (β-CD)	Supramolecular systems based on coupling of adamantane-grafted PEI with L-cystine-bridged bis(β-CD) (PEI-Ada-LCD)	The optimized PEI-Ada-LCD formulation showed almost 2-fold higher gene transfection efficiency (54%) than that of the gold standard i.e., PEI25 KDa (~32%) in 293T cells. Moreover, PEI-Ada-LCD was less cytotoxic toward 293T cells as compared to PEI25 KDa.	[56]
pDNA	Polyethyleneimine 600 (PPC)	β-cyclodextrin (β-CD)	Epsilon-polylysine-grafted-succinic acid-grafted-β-CD- PPC entrapped with adamantane-functionalized poly-(ethylene glycol) derivative (PEG-AD)	The pDNA transfection efficiency of PPC/PEG-AD was almost similar to PEI25 KDa. However, PPC/PEG-AD showed extremely lower cytotoxicity to HEK293 cells (~95% viability) as compared to PEI25 KDa (~10% viability) at an equivalent concentration.	[57]

miR-34a	Polyethyleneimine (PEI)	β-cyclodextrin (β-CD)	β-CD-PEI cationic polymers grafted with polyethylene glycol and matrix metalloproteinase (MMP)-2 cleavable peptide (CD-PEI-C-PEG)	During the in vivo study, CD-PEI-C-PEG polyplexes exhibited significantly higher tumor inhibition potential (51.79%) than that of CD-PEI (7.01%).	[63]
miR-34a-5p	Polyethyleneimine (PEI)	β-cyclodextrin (β-CD)	Conjugates grafted folic acid (β-CD-PEI-FA)	As compared to the control, β-CD-PEI-FA showed 306.12 and 2.73 times higher miR-34a-5p expression in BCBL-1 and SK-RG cells. Moreover, β-CD-PEIFA showed up to 70% viability of BCBL-1 and SK-RG cells, hence confirming their biocompatibility.	[64]
siRNA	Polyethyleneimine	2-hydroxypopyl-β-cyclodextrin (β-CD)	Conjugates functionalized with folic acid (FA-PEI-HP-β-CD)	FA-PEI-HP-β-CD completely condensed the siRNA into 250 nm particles at 24 N/P ratio. FA-PEI-HP-β-CD/siRNA were internalized by folate receptor enriched HeLa cells to a greater extent (4-fold) than non-targeted PEI-HP-β-CD while maintaining 90% cell viability. FA-PEI-HP-β-CD protected up to 90% of siRNA from nuclease degradation within 12 h while naked siRNA was completely degraded during the specified period.	[67]
DNA	Polyethyleneimine 600 (PEI 600)	β-cyclodextrin (β-CD)	Amphiphilic β-CD based vectors	PEI600-β-CD showed better gene transfection efficiency (~110% vs ~80% relative to control) than that of PEI25 KDa in 10% serum while both carriers showed almost similar efficiency i.e., 100% in a serum-free environment. Moreover, PEI600-β-CD were less cytotoxic to 293T cells at all the tested concentrations (10-100 μg/mL) i.e., ~60-90% viability vs ~50-80%. Polyplexes were up internalized through caveolae-mediated endocytosis which could prevent the lysosomal degradation of entrapped genes.	[71]
siRNA	Polyethyleneimine 600 (PEI 600)	β-cyclodextrin (β-CD)	Polyethylene glycol-chitosan grafted-PEI- β-CD copolymers (PEG-CT-PEI-β-CD)	Standard PEI25 KDa/siRNA showed 20-30% luciferase knock down while CT-PEI-β-CD/siRNA resulted in 60% knock down in L929 cells. PEGlyation of these carriers resulted in 84% luciferase knockdown which was comparable to that of commercial DharmaFECT.	[76]

pDNA	Polyethylenimine 600 Da (PEI)	Hydroxypropyl-γ-cyclodextrin (HP-γ-CD)	Polymeric carriers grafted with MC-10 oligopeptide (HP-γ-CD-PEI-P)	The HP-γ-CD-PEI-P showed 4 and 3.7-fold higher pDNA transfection efficiency to SKOV-3 cells than that of PEI25 KDa and non-functionalized HPg-CD-PEI, respectively. Moreover, HP-γ-CD-PEI-P didn't show any noticeable cytotoxicity towards SKOV-3 cells up to 120 N/P ratio while standard PEI25 KDa showed less than 20% cell viability at 40 N/P ratio.	[60]
siRNA + Doxorubicin (DOX)	Polyethylenimine	β-cyclodextrin	Folic acid grafted- magnetic hollow mesoporous silica NPs (FA- MHMSNs)	Free DOX was unable to impart cytotoxic impact (IC_{50} > 500 μg/mL) while FA- MHMSNs NPs showed remarkable cytotoxicity towards MCF-7/ADR cells (IC_{50} > 6.20 μg/mL) which was further increased under the influence of alternating magnetic field (IC_{50} > 4.42 μg/mL).	[78]
**Oligoethylenimine**					
pDNA	Oligoethylenimine (OEI)	α-cyclodextrin (α-CD)	Cationic star polymer (α-CD-OEI) based on grafting of OEI arms onto an α-CD core	The star polymer exhibited 50-fold higher pDNA transfection efficiency in HEK293 cells than that of PEI25 KDa in a serum-free environment however, both agents showed comparable transfection efficiencies under serum conditions. Moreover, α-CD-OEI was less cytotoxic to HEK293 cells (~35% viability) as compared to PEI25 KDa (~5% viability).	[54]
pDNA	Oligoethylenimine (OEI)	γ-cyclodextrin (γ-CD)	Sar-shaped polymers grafted with folic acid through disulfide bonds (γ-CD-OEI-SS-FA)	The optimized γ-CDOEI-SS-FA formulation showed a 6-fold higher gene transfection efficiency in KB cells as compared to standard PEI25 KDa while imparting relatively less cytotoxic impacts.	[59]
pDNA	Oligoethylenimine (OEI)	α-cyclodextrin (α-CD)	Fe_3O_4 nanoparticles functionalized with α-CD-OEI	The implication of magnetic field improved the gene transfection efficiency of nanocarriers up to 10-fold. Magnetic field mediated the accumulation of polyplexes on the cell membrane and facilitated their penetration into the cell.	[62]
MMP-9 shRNA plasmid (pMMP-9) +	Oligoethylenimine	β-cyclodextrin	Conjugates	The resultant carriers exhibited > 50% transfection efficiency to MCF-7 cells while standard PEI 25KDa showed only 17% transfection efficiency. The tumor-bearing mice treated with these carriers showed only 21% tumor volume as compared to phosphate buffer saline.	[79]

Doxorubicin (DOX)					
**Quaternary ammonium**					
Methotrexate (MTX)	Imidazole and quarternary ammonium	β-cyclodextrin (β-CD)	Nanoparticles (NPs)	MTX-loaded NPs showed relatively higher cytotoxicity (IC_{50} 7.22 μg/mL) than free MTX (IC_{50} 7.82 μg/mL) towards Saos-2 bone cancer cells. Moreover, flow cytometry cell analysis revealed that 9.21% of the MTX-loaded NPs were taken up by the Saos-2 cells during 1 hour	[6]
Doxorubicin (DOX)	Quaternary ammonium	β-cyclodextrin	Nanoparticles (NPs)	Cationic cyclodextrin NPs improved the permeability of DOX across BBMVEC monolayer up to 2.2-fold. NPs were less cytotoxic to BBMVEC monolayer than free DOX. Moreover, NPs killed U87 tumor cells as effectively as bare DOX.	[9]
4-hydroxy-tamoxifen (TMX)	Quaternary ammonium	β-cyclodextrin	Cationic poly(cyclodextrin)/ alginate nanocapsules	In vitro study demonstrated that nanocapsules can efficiently deliver the TMX to immortalized mouse podocyte cells which mediates Cmip exon 8 deletion.	[10]
5-Fluorouracil (5-FU)	Quaternary ammonium	β-cyclodextrin	Cationic-β-CD loaded alginate/chitosan nanoflowers	The nanoformulation showed a pH-dependent and sustained 5-FU release profile up to 24 h. Further, in vivo studies are still in progress to evaluate the effectiveness of these nanoflowers in animal models.	[11]
Camptothecin (CPT)	Quaternary ammonium	Polycationic ß-cyclodextrin derivative (PC ßCDC6)	Nanoparticles (NPs)	NPs showed a promising mucus layer penetration rate (73%) and released a smaller CPT content (48%) in GF and SIF, hence, demonstrating the potential for colon-targeted drug delivery. Moreover, nanoformulation reduced the viability of HT-29 cells (52.44%) to a greater extent than free CPT solution (83.98%) at an equivalent concentration (0.1 μg/mL)	[12]
Indomethacin (IDM)	Quaternary ammonium	β-cyclodextrin	Hydrogel	The solubility of IDM (0.0615 mmol/L) was 100-fold enhanced after complexation with cationic β-CD (6.5 mmol/L). In simulated gastrointestinal fluid, the hydrogel was completely swollen within 14-16 h and 100% of IDM was released within 21 h. Hence, these hydrogels can serve as a controlled release system for IDM.	[18]

Dexamethasone (DXM)	Quaternary ammonium chitosan (QA-Ch60)	Methyl-β-cyclodextrin (MCD)	Conjugates	The conjugates formed a stable complex with DXM while improving its aqueous solubility and retained significant mucoadhesion. Moreover, conjugate-treated RCE cell lines showed high viability (80%) hence, confirming their biocompatibility.	[19]
Naproxen (NPX)	Quaternary ammonium	β-cyclodextrin (β-CD)	Inclusion complexes	The cationic β-CD complexation improved the aqueous solubility of NPX up to 120-fold. Conjugates exhibited a faster dissolution rate and higher amount of drug dissolution. Moreover, cationic β-CD exhibited relatively low hemolytic activity compared with parent β-CD hence, confirming the biocompatibility of these drug carriers.	[22]
α-mangostin (α-MG)	Quaternary ammonium and chitosan	β-cyclodextrin (β-CD)	Quaternized β-CD grafted-chitosan (QCD-g-CS) based inclusion complexes	QCD-g-CS/α-MG complexes showed an initial burst release of α-MG (up to 5 h) followed by sustained release (up to 24 h). QCD-g-CS/α-MG showed significantly lower MIC i.e., 0.6 and 1.25 mg/mL for *Streptococcus mutans* and *Candida albicans* while bare α-MG showed >10 mg/mL MIC for both microbes.	[44]
Insulin	Quaternary ammonium	β-cyclodextrine (β-CD)	alginate/chitosan nanoparticles (NPs)	Simple alginate/chitosan NPs released up to 60% insulin in simulated gastric fluid (pH 1.2) while only 18% of insulin was released in simulated intestinal fluid (pH 6.8). Hence, most of the payload was lost before it could reach its target site (intestine) to show any therapeutic effect. The optimized CPβCDs-insulin-loaded alginate/chitosan NPs released 48% of insulin in simulated gastric fluid while 40% of insulin was successfully liberated in simulated intestinal fluid.	[49]
Heparin	Quaternary ammonium	β-cyclodextrin	Self-nano-emulsifying drug delivery system (SNEDDS	The cumulative amount of heparin liberated from SNEDDS after 120 min incubation in SGF (pH 1.2) followed by further 240 min incubation in SIF (pH 6.8) was 47.31%. Although,100% of the payload was not released while it was referred that considering the physiological emptying time in gastrointestinal tract, studying the in vitro release profile for 6 h is needed for the prediction of system behavior under in vivo.	[51]

pDNA (luciferase plasmid (pCMV-Luc))	Primary, tertiary, and quaternary ammonium groups	β-cyclodextrin	Cationic star polymers with 21 arms (21ACSPs)	21ACSPs with primary and tertiary amino groups showed satisfactory transfection efficiency to CHSE-214 cells i.e., ~23 and ~28 ng luciferase/mg protein. However, quaternary ammonium containing 21ACP was unable to show any transfection efficiency as the quaternary ammonium cannot be further protonated in the acidic environment of the endosome. Moreover, transfection with 21ACSP/pDNA polyplexes showed high viability of CHSE-214 cells (77-88%) thus, confirming their biocompatibility.	[55]
EGFP-mRNA, OVA-mRNA	Quaternary ammonium	β-cyclodextrin	Hyper-branched cyclodextrin-based polymer nanoparticles (Ppoly)	77%, 72%, and 26% EGFP-mRNA was taken up by B16–F10 cells at 1:10, 1:5, and 1:1 N/P ratios of Ppoly while 58% lipofectamine treated EGFP-mRNA was internalized at 3:1 N/P ratio. As compared to the untreated group, OVA-mRNA exhibited three times higher tumor suppression by triggering a robust adaptive immune response.	[74]
Triclosan (TR)	Quarterny ammonium	β-cyclodextrin	Inclusion complexes	The water solubility of TR increased linearly until the solubility limit was achieved i.e., 1.4 mg/mL TR with 3.8 mg/mL cationic β-CD. Moreover, cationic β-cyclodextrin polymers were considered biocompatible due to the high viability (99%) of human colon carcinoma Caco-2 cells.	[41]
Triclosan (TR) and Butylparaben (BP)	Quarterny ammonium	β-cyclodextrin (β-CD)	Inclusion complexes	The *E. coli* growth inhibition potential of TR/ β-CD was higher than that of BP/β-CD at lower than 0.5% concentration while above 0.5% concentration both antibiotics exhibited 100% inhibition of bacterial growth. Hence, TR/β-CD complex was more potent than BP/(β-CD). However, BP complex showed faster growth inhibition rated than TR complex in a short contact time (10 minutes).	[42]
**Ammonium chloride**					
Paclitaxel (PTX)	Ammonium chloride	β-cyclodextrin (β-CD)	Nanoparticles (NPs)	The NPs reduced the viability of MCF-cell lines to a greater extent 30.7% than NPs based on non-ionic β-CD derivative 51.7%.	[8]
Rebamipide (REB)	*N,N,N*-trimethyl-N-(2-hydroxy-3-	β-cyclodextrin (β-CD)	Conjugates (CDQA)	The solubility of rebamipide was enhanced to a significant extent in CDQA solution (10.27 μM concentration) as	[52]

	metacryloyloxopr opyl)-ammonium chloride (QA)			compared to that in β-cyclodextrin solution (3.8 μM concentration). Furthermore, the CDQA solution promoted rebamipide penetration across the cornea. The instillation of REB@CDQA resulted in higher lacrimal fluid volume (1.3-fold) and mucin levels (1.5-fold) as compared to the control (rebamipide suspension) along with significant attenuation of tear film breakup.	
			**Chitosan**		
Indomethaci n (IDM)	Chitosan	β-cyclodextrin (β-CD)	Nanoparticles (NPs)	The hydrogel attained 90% swelling at pH 1.4 and 60% swelling at pH 7.4, within 6 h. Consequently, a higher drug release rate was noticed at pH 1.4 (~85%) than that at pH 7.4 (~57%). Hence, this cationic-cyclodextrin-based formulation can be employed for targeted and controlled drug delivery.	[16]
Indomethaci n (IDM)	Chitosan (CS)	β-cyclodextrin	Electrospun nanofibers	Nanofibers showed a sustained release of IDM and equilibrium was attained within 125 h while ~80% payload was liberated within 300 h. Moreover, nanofibers were biocompatible as the growth of L929 cells was not inhibited to a significant extent.	[17]
Ketoprofen (KTP)	Chitosan (CS)	β-cyclodextrin (β-CD)	Nanoparticles (NPs)	CD-g-CS NPs exhibited a sustained release profile at pH 6.8 than that of chitosan NPs and KTP release could be further slowed with increasing substitution degree of cyclodextrin thus reducing the need for frequent dosing of KTP.	[23]
Ketoprofen (KTP)	Chitosan	β-cyclodextrin	Nanoparticles (NPs)	NPs exhibited a sustained KTP release profile in *PBS (at pH 7.4) and attained the equilibrium after 23 h while bare chitosan nanoparticles liberated the entire payload within 9 h. Moreover, NPs were considered biocompatible based on the high viability of L929 cells.	[24]
Ciprofloxacin (CFX)	Quaternized chitosan	Not-specified	Nanoparticles (NPs)	The nanoformulation initially released the CFX at a higher rate (~35% within 0.5-1 h) due to the desorption of the surface-adsorbed drug. Subsequently, CFX was released in a sustained manner (~90% within 24 h) which was mediated by CFX diffusion through the NPs matrix and erosion of the polymeric matrix. Moreover, nanoformulation showed a	[31]

				highly potent antibacterial activity for both Staphylococcus aureus and Escherichia coli (MIC 6.25 μg/mL).	
Silver sulfadiazine (SSD)	Chitosan	Not specified	Supramolecular polyelectrolyte complexes (SPEC)	The SSD/SPEC showed smaller zones of inhibitions than that of free SSD i.e., 18 vs 25 mm, 18 vs 20 mm, 17 vs 23 mm for *S.aureus*, *Klebsiella pneumoniae*, and *E. coli*. The improved antibacterial activity might be attributed to the electrostatic interactions between positively charged amine groups of chitosan with the negatively charged bacterial cell wall components.	[34]
Sulfadiazine (SSD) Sulfamonomethoxine (SMMX) Sulfamethoxazole (SMZ)	Chitosan (CS)	β-cyclodextrin (β-CD)	Inclusion complexes based on CD-grafted with β-CD (CD-g-CS)	The aqueous solubilities of free SSD, SMMX, and SMZ were noticed as 0.026, 0.069, and 0.329 mg/mL, respectively. However, CD-g-CS-based SSD, SMMX, and SMZ complexes showed significantly improved solubilities up to 5.6-fold (0.147 mg/mL), 4.1-fold (0.283 mg/mL), and 2.3-fold (0.747 mg/mL), respectively.	[38]
Levofloxacin (LVX)	Chitosan (CS)	β-cyclodextrin (β-CD)	CS-β-CD inclusion complexes	Simple β-CD conjugates released 100% LVX in 180 min while CS-β-CD complexes released the complete payload in 800 h. Hence, CS-β-CD complexation provided a 4-fold slow drug release in comparison to bare β-CD. These complexes can serve as sustained drug delivery systems.	[39]
**Lysine**					
Scutellarin (SCU)	Poly(ε-lysine) (ε-PL)	β-cyclodextrin (β-CD)	ε-PL and glycine β-CD (ε-PL-GLY-CD) based inclusion complexes	ε-PL-GLY-C exhibited improved aqueous solubility of SCU (52.82 mg/mL) than that of bare SCU (0.16 mg/mL). Moreover, these inclusion complexes exhibited higher cytotoxicity against HCT116 and LOVO cells (IC_{50} 8.2 and 19.4 μM) as compared to bare SCU (IC_{50} 72.3 and 80.6 μM), respectively.	[14]
siRNA	Lysine amino acid	β-cyclodextrin (β-CD)	Conjugates	The resultant polyplexes were efficiently internalized by prostate cancer cells (DU145, VCaP, and PC3 cells) while maintaining > 80% cell viability. The nanoplexes exhibited efficient silencing of the PLK1 gene which is involved in numerous types of cancers. The carriers protected siRNA	[65]

				from nuclease digestion for up to 24 h while naked siRNA was partially degraded within 8 h.	
OligoRNA + Doxorubicin	Poly-L-lysine	β-cyclodextrin	Nanocomplexes grafted with hyaluronic acid (HA-NPs)	HA-NPs showed higher toxicity to MHCC-97H cells (IC_{50} 6.58 µg/mL) than that of non-functionalized NPs (IC_{50} 11.26 µg/mL). HA-NPs were mostly distributed in the tumor tissues while non-functionalized NPs were distributed both in the liver and tumor tissues.	[77]
pDNA + Methotrexate (MTX)	Poly(L-lysine) dendron (PLLD)	6-azido-β-cyclodextrin (β-CD)	Conjugates	The resultant carriers released MTX in a more sustained manner (~45%) than that of β-CD (~50%) and PLLD (~70%) within 25 h. CD-PLLD-MTX treated MCF-cells exhibited < 70% viability at 50 µg/mL concentration.	[81]
MMP-9 siRNA plasmid (pMR3) + Docetaxel (DOC)	Poly(L-lysine) dendron (PLLD)	6-azido-β-cyclodextrin (β-CD)	Conjugates	CD-PLLD/DOC/pMR3 showed greater apoptosis of HNE-1 cells (55.5%) as compared to CD-PLLD/DOC (13.8%) and CD-PLLD/pMR3 (40.7%). The greater antitumor potential of CD-PLLD/DOC/pMR3 was attributed to the fact that released DOC could induce DNA damage while pMR3 could mediate the down-regulation of protein expression.	[80]
**Polyamidoamine**					
Ciprofloxacin (CFX)	Polyamidoamine	β-cyclodextrin (β-CD)	Nanoparticles (NPs) on polyester fabric	The resultant fabric showed higher drug absorption (16.9% vs 3.7% after 24 h) and sustained drug release (45% vs 92% after 3 h) as compared to raw fabric. Modified fabric showed 100% antibacterial efficiency for both *E.coli* and *S. aureus*. Moreover, the fabric didn't impart any cytotoxic impact on fibroblast cells, hence confirming its biocompatibility.	[32]
Nitric oxide (NO)	Poly(amidoamine) dendron (PAMAM)	β-cyclodextrin (β-CD)	β-CD-PAMAM/NO inclusion complexes	β-CD-PAMAM/NO complexes (50 µg/mL), showed 80% and 94% biofilm inhibition for *E. coli* and *S. aureus* within 18 h. Moreover, β-CD-PAMAM/NO exhibited excellent biocompatibility and didn't impart any cytotoxic impact on NIH 3T3 cells.	[43]
MMP-9-siRNA	Poly(amidoamine)	β-cyclodextrin (β-CD)	Cationic star-shaped polymers consisting of	β-CD-PAMAM and standard lipofectamine 2000 exhibited transfection efficiency of 98.78% and 64.89% while inducing 5.57% and 1.21% fibroblast cell death. Furthermore, MMP-9 expression was reduced by 68% and 80% for the cells treated	[68]

				with β-CD-PAMAM/MMP-9-siRNA and lipofectamine 2000/MMP-9-siRNA, respectively.	
siRNA + Methotrexate (MTX)	Poly(amidoamine) dendrons (PAMAM)	Per-6-azido-β-cyclodextrin	Star-shaped polymers	The carriers showed better transfection efficiency to fibroblast cells than PAMAM dendrimers i.e., ~85% vs ~75% with serum and ~97% vs ~82% without serum. The carriers released the DOX in a sustained manner for up to 10 h.	[81]
**Poly (2-(dimethylamino) ethyl methacrylate) (PDMAEMA)**					
Doxorubicin (DOX)	Poly (2-(dimethylamino) ethyl methacrylate) (PDMAEMA)	β-cyclodextrin (β-CD)	Star polymer	The DOX-loaded star polymers inhibited tumor growth at a higher rate (62.4%) than free DOX (37.2%) in xenograft mice bearing human cervical cell lines.	[2, 3]
Methotrexate (MTX)	Poly (2-(dimethylamino) ethyl methacrylate) (PDMAEMA)	β-cyclodextrin	Hydrogels	Hydrogels showed higher cytotoxicity (IC_{50} 27 μg/mL) towards MCF-7 cells than free MTX (IC_{50} 55 μg/mL) and showed pH sensitive drug release profile (32.56% at pH 7.4 vs 85.3% at pH 5.2).	[7]
Dexamethasone (DXM)	Poly (2-(dimethylamino) ethyl methacrylate) (PDMAEMA)	β-cyclodextrin (β-CD)	β-CD-graft-(poly(ε caprolactone)-block- PDMAEMA (β-CD-g-(PCL-PDMAEMA) star-like amphiphilic polymer-based inclusion complex	β-CD-g-(PCL-b-PDMAEMA-loaded DXM efficiently reduced the lipopolysaccharide-mediated release of cytokines (interleukin-1β, interleukin-6, and interleukin-10) in RAW264.7 macrophages than that of free DXM.	[21]
pDNA	Poly(2-(dimethylamino) ethyl methacrylate (PDMAEMA)	β-cyclodextrin (β-CD)	Star-like amphiphilic β-CD-graft-(poly(ε-caprolactone)-block(PDMAEMA)x (β-CD-g-(PCL-b-PDMAEMA)x) copolymer	β-CD-g-(PCL-b-PDMAEMA)x showed significantly higher pDNA transfection efficiency in RAW264.7 macrophages (10.8%) than that of standard lipofetamine (2.6%). Moreover, the copolymer showed low cytotoxicity (IC50 40 μg/mL) hence, confirming its biocompatibility	[21]
pDNA	Poly(2-(dimethylamino) ethyl methacrylate (PDMAEMA)	β-cyclodextrin (β-CD)	β-CD grafted-poly(ε-caprolactone)- PDMAEMA copolymer-based vectors	β-CD-based vectors showed significantly higher gene transfection efficiency than that of PEI 25 KDa i.e., 84% vs 52.9% in HEK293T cells and 23.6% vs 10.6% in HepG2 liver cancer cells. Moreover, β-CD based vectors were less cytotoxic to both cell lines (up to 67.8% viability) as compared to PEI25 KDa (12.7% viability).	[72]

**Other amine/amino groups**					
Doxorubicin (DOX) + Celastrol (CSL)	Mono-(6-pentaethylenehexa amine) (PEHA)	β-cyclodextrin (β-CD)	Nanoparticles (NPs)	The DOX(CSL)-loaded PEHA-β-CD NPs efficiently mediated the apoptosis of colon cancer cells (SW480) and liver cancer cells (SMMC-7721). Moreover, NPs showed lower toxicity to (IC_{50} 0.57 μg/mL) normal epithelial cells (BEAS-2B) as compared to free DOX (IC_{50} 0.21 μg/mL)	[1]
Doxorubicin (DOX)	Poly(ß-amino ester)	β-cyclodextrin (β-CD)	Nanoparticles (NPs)	The NPs showed 100% and 60% higher permeability coefficients than that of the dextran control across monolayers of bovine brain microvascular endothelial cells (BBMVECs) and human brain microvascular endothelial cells (HBMVECs).	[4]
Scutellarin (SCU)	Triethylenetetrami ne	β-cyclodextrin	Inclusion complexes	Complexes showed improved water solubility (50.7 mg/mL) than that of free SCU (0.16 mg/mL). Furthermore, the cytotoxic potential of these inclusion complexes was remarkably higher towards HCT116 and LOVO cells ($IC5_0$ 0.9, 8.3 μM) than that of free SCU (IC_{50} 72.3, 80.6 μM), respectively.	[15]
Dexamethaso ne (DXM)	(6-aminohexyl) amino	β-cyclodextrin (β-CD)	Gellan gum complex hydrogel	The cell-cultured Dx@HCD-GG hydrogel showed the highest glycosaminoglycan (GAGs) and double-stranded DNA (dsDNA) contents indicating the enhanced chondroprotective effect of DXM. During the in vivo study (cartilage defect model), the hydrogel-treated group showed highly dense regenerated tissues which were well interacted with the surrounding tissues. Moreover, the formation of glycosaminoglycan matrix was also noticed, hence confirming the cartilage regeneration potential of Dx@HCD-GG hydrogel.	[20]
Meloxicam (MLX)	Triethanolamine (TEA)	β-cyclodextrin (β-CD)	Conjugates	The resultant MLX-β-CD-TEA conjugate showed significantly improved dissolution with ~85% cumulative drug dissolved than that of pure MLX (~30%). MLX-β-CD-TEA ternary complexes showed higher edema inhibition (84.38%) as compared to pure MLX (59.37%).	[29]
Diclofenac sodium	Amino groups	Poly-β-amino-cyclodextrin	Nanoassemblies	PolyCD-based nanoassemblies were efficiently taken up by hMSCs (within 2 h) without imparting any cytotoxic impact.	[25]

(DCF)		derivative		Subsequently, interleukin-1β and tumor necrosis factor-α levels were reduced to a significant extent as compared to other treatment groups. Moreover, DCF was released in a controlled manner (~35% within 10 days).	
Vancomycin (VCM)	Oleylamine (OLA)	β-cyclodextrin (β-CD)	Cationic amphiphile derivative	βCD-OLA released the VCM in a sustained manner i.e., ~65% and ~80% after 24 h and 48 h, respectively. Furthermore, BCD-OLA/VCM showed a 4-fold reduced MIC (7.81 μg/mL) towards Methicillin-resistant *S. aureus* as compared to free vancomycin (31.25 μg/mL). BCD-OLA/VCM caused the 459-fold reduction of intracellular bacteria using infected human embryonic kidney cells (HEK), and an 8-fold reduction in infected macrophages as compared to free vancomycin.	[33]
Linezolid (LZD)	Amino groups	β-cyclodextrin	Au@Ag bimetallic nanoparticles (BMNPs)	MIC values of LZD loaded NPs and free LZD were noticed as 2 vs 1.95 μg/mL for MRSA, 2 vs 0.97 for *S.aureus*, 2 vs 15.6 μg/mL for *E. coli*, 3.9 vs 15.6 μg/mL for *P. aeruginosa*. Hence, LZD-nanoformulation showed a broad spectrum of activity than free LZD.	[40]
Oxacillin (OXA)	Aminoethylthio	γ-cyclodextrin (γ-CD)	Complex	It was demonstrated that γ-CD complex resulted in a 2.3-fold reduction of β-lactamase induced OXA hydrolysis. Moreover, γ-CD complex was efficiently internalized by macrophages i.e., 25% internalization in the first 15 minutes while 99.8% internalization within 24 h. Moreover, γ-CD complex was found as biocompatible based on the viability of L929 cells.	[35]
Rifampicin (RFP)	Aminoethylthio	γ-cyclodextrin (γ-CD)	Complex	Drug-loaded RFP/γCys complex reduced biofilm viability to the background levels (~100%) while Free RFP showed moderated anti-biofilm activity (~60% reduction). The remarkable anti-biofilm potential of γCys/RFP might be attributed to the improved solubility of RFP upon complexation and/or synergistic interference with components of the biofilm.	[36]

Metformin hydrochloride (MTF)	Diaminodipropylamine	β-cyclodextrine (β-CD)	Nanoparticles (NPs)	Drug-loaded NPs released a significantly lower amount (7.55%) of MTF in a simulated gastric fluid (pH 2) than that of MTF released from solution form (13%). Moreover, NPs released MTF at higher rates with increasing pH values as compared to the drug solution i.e., 33.68% vs 17% at pH 6.3 and 61.98% vs 27% at pH 8. Hence, these NPs can reduce the degradation of MTF in the stomach and promote its absorption in the small intestine	[45]
Liraglutide (LTD)	Propyl-amine	Not specified	Nanoparticles (NPs)	The nanoformulation could prevent the degradation of LTD upon incubation with simulated intestinal fluid supplemented with enzymes (up to 4 h) while the LTD solution rapidly underwent degradation (within 5 min). Followed by intestinal administration, LTD-NPs reduced the glucose load (71%.hr) which was quite close to the hypoglycemic effect (68%.hr) of subcutaneous LTD solution.	[46]
Insulin	Diethylenetriamine (DETA)	β-cyclodextrine (β-CD)	Poly(glycidyl methacrylate)s (PGOHMAs) based polyelectrolyte complexes (PECs)	The cumulative release of insulin from CD-series complexes (~80-95%) was higher than that of D-series complexes (~57-67%). The CD-series showed less toxicity towards L929 cells (nearly 100% viability) than the D-series (~10-70% viability) hence, the introduction of cyclodextrin mitigated the toxicity of amino PGOHMA by decreasing the density of amino groups.	[48]
Daidzein (DAI)	Glycidyltrimethyl ammonium chloride (GTMAC) and ethylenediamine (EDA)	γ-cyclodextrin (GCD)	Conjugates	At an equivalent concentration (100 μg/ml) of GCD-GTMAC/DAI and GCD-EDA/DAI, levels of the cellular glycosaminoglycans were dropped down to 72% and 62% of the control, respectively. It was suggested that such cationic GCD derivatives-based daidzein inclusion complexes may be employed to reduce the accumulation of glycosaminoglycans in mucopolysaccharidoses and lysosomal storage diseases.	[53]
Vitamin B_2	Guanidine	β-cyclodextrin	Conjugates	The aqueous solubility of vitamin B_2 conjugates (673 g/L) was significantly higher than that of bare vitamin B2 (0.078 g/L). Moreover, conjugates released the payload in a	[50]

				sustained manner i.e., 89.1%, 71.5%, and 91.7% at pH 10, 7.4, and 1.2 after 23 h.	
siRNA	*N,N′*-dimethylethylene diamine (DMEDA)	*α*-cyclodextrin (*α*-CD)	*α*-CD:poly(ethylene glycol) polyrotaxanes	The cationic polyrotaxanes exhibited > 10^2-fold lower cytotoxicity than the standard PEI25 KDa. Moreover, polyrotaxanes showed comparable gene silencing efficiencies (60-70% reduced GFP expression) to those of Lipofectamine 2000 and PEI25 KDa.	[69]
siRNA	Spermidine amino acid	β-cyclodextrin (β-CD)	β-CD: adamantane - poly (vinyl alcohol)- poly (ethylene glycol) (β-CD: Ad-PVA-PEG) complexes	The optimized β-CD: Ad-PVA-PEG complexes showed 90.5% siRNA binding efficiency. The complexes showed almost equivalent siRNA uptake to A549 cells and cell viability (67% vs 65%) to the standard lipofectamine.	[75]
pDNA (luciferase-encoding plasmid DNA, pCMVLuc)	Amino groups	β-cyclodextrin (β-CD) derivative T2	Polypexes grafted with folic acid (fol-CDplexes)	The fol-CDplexes showed significantly higher gene transfection efficiency than that of plain-CDplexes and standard PE125 KDa polyplexes in HeLa cells i.e., ~68, ~38, ~18 ng luciferase/mg protein, respectively. Further in vivo study demonstrated that gene expression induced by Fol-CDplexes was 2-fold and 4.6-fold higher in liver and lung tissues, respectively as compared to plain-CDplexes	[58]
pDNA	Dimethylaminoethyl (DMAE)	*α*-cyclodextrin (*α*-CD)	Polyrotaxanes based on cationic *α*-CD and disulfide-grafted poly(ethylene glycol) (PEG)	The polyrotaxanes formed stable polyplexes at 0.5 N/P ratio with positive zeta potential (+4.8 mV) while LPEI22k was unable to form compact polyplex and showed negative zeta potential. Moreover, polyrotaxane exhibited concentration-dependent transfection efficiency and precluded lysosomal degradation of pDNA due to the proton sponge effect.	[61]
siRNA	Guanidinium (GD)	β-cyclodextrin (β-CD)	GD and polyethylene glycol attached with primary and secodary surface of β-CD, respectively and conjugate grafted with anisamide (G-CD-PEG-AA)	G-CD-PEG-AA induced prostate cell-specific internalization of siRNA resulting in approximately 80% knockdown luciferase (reporter gene). Followed by intravenous administration, G-CD-PEG-AA/vascular endothelial growth factor (VEGF) siRNA exhibited a 3-times reduction in tumor volume as compared to phosphate buffer saline.	[66]
pDNA	Tetraethylenepentamin (TEPA)	β-cyclodextrin (β-CD)	TEPA-β-CD nanoparticles	TEPA-BCD based polyplexes were efficiently internalized by pigment epithelial cell line and a mouse embryonic fibroblast cell line (3T3) through clathrin-and caveolae-	[73]

				mediated endocytosis. TEPA-β-CD/pDNA showed 97% transfection efficiency while maintaining 83% cell viability.	
**Surfactants**					
Melphalan (MLP)	Gemini surfactant	β-cyclodextrin	Inclusion complexes	MLP complexes showed a significantly reduced IC_{50} than free MLP (27 μM vs 98 μM) against A375 cell lines. Hence, the inclusion of MLP with cationic-cyclodextrin can improve its efficacy.	[13]
Ketotifen hydrogenfumarate (KHF)	HHDDP surfactant	γ-cyclodextrin (γ-CD)	Hyaluronic acid (HA)/γ-CD/HHDDP polymer assemblies	The water-insoluble polymer matrix released KHF (water-soluble drug) slowly while free KHF was completely dissolved in a physiological saline solution over 35 min. Hence, this delivery system can be employed to attain a sustained release profile of water-soluble drugs.	[28]
Budesonide (BUD)	Benzalkonium chloride	β-cyclodextrin (β-CD)	Hydrogel	The resultant hydrogel film showed improved dissolution (87.2% vs 63%) and mucosal permeation (95.8% vs 40.2%) as compared to film without cyclodextrin and benzalkonium. The topical application of the formulation to the rabbit eye was capable of reducing the symptoms of inflammation (redness) within 3 h.	[30]
**K^+ cations**					
Ibuprofen (IBU)	K^+ cations	β-cyclodextrin	Metal-organic frameworks (MOFs)	The aqueous solubility of IBU entrapped in MOFs was improved up to 17-fold.	[26]
Niflumic acid (NIF)	K^+ cations	γ-cyclodextrin	Metal-organic frameworks (MOFs)	MOFs showed a pH-dependent drug release i.e., 14% NIF in 1 h and 40% NIF in 6 h at pH 1.6 while 40% NIF in 2 h and the remaining amount was completely released in 8 h. NIF incorporated in the frameworks also showed improved solubility.	[27]
Enrofloxacin (ENF)	Potassium ions	γ-cyclodextrin (γ-CD)	Metal-organic frameworks (MOF)	The γ-CD-MOF released 40% of ENF in 1 h and 87.5% of ENF in 4 h. The γ-CD-MOF/NF showed ~100% bacterial inhibition in 6 min and retained ~95% bacterial inhibition till 24 min. However, free ENF exhibited ~85% bacterial inhibition in 6 min while ~70% inhibition in 24 min. Hence, γ-CD-MOF/NF exhibited a higher and longer bacterial growth inhibition potential as compared to free ENF.	[37]
**Other**					

siRNA	Ionene	α-cyclodextrin + β-cyclodextrin	Polyrotaxanes	Polyrotaxanes mediated the siRNA internalization into A549 cell line (10 times higher fluorescence intensity than that of original cells) while exhibiting 60% knockdown efficiency of luciferase gene.	[70]

Abbreviations**:** BBMVEC; bovine brain microvascular endothelial cell, SGF; simulated gastric fluid, SIF; simulated intestinal fluid; PBS; phosphate buffer saline, hMSCs; human mesenchymal stromal cells, HHDDP; [hexadecyl(2-hydroxyethyl)dimethylammonium dihydrogen phosphate, MRSA, methicillin-resistant *Staphylococcus aureus*, LPEI22k ; linear polyethyleneimine with 22000 molecular weight, GFP; green fluorescent protein, PEI; polyethyleneimin, EGFP; enhanced green fluorescent protein, OVA, oval albumin.

References

1. Li, B.L.; Zhang, J.; Jin, W.; Chen, X.Y.; Yang, J.M.; Chi, S.M.; Ruan, Q.; Zhao, Y. Oral administration of pH-responsive polyamine modified cyclodextrin nanoparticles for controlled release of anti-tumor drugs. *React. Funct. Polym* **2022**, *172*, 105175-105187
2. Zhang, M.; Xiong, Q.; Chen, J.; Wang, Y.; Zhang, Q. A novel cyclodextrin-containing pH-responsive star polymer for nanostructure fabrication and drug delivery. *Polym. Chem.* **2013**, *4*, 5086-5095.
3. Xiong, Q.; Zhang, M.; Zhang, Z.; Shen, W.; Liu, L.; Zhang, Q. Anti-tumor drug delivery system based on cyclodextrin-containing pH-responsive star polymer. in vitro and in vivo evaluation. *Int. J. Pharm* **2014**, *474*, 232-240.
4. Gil, E.S.; Wu, L.; Xu, L.; Lowe, T.L. β-Cyclodextrin-poly (β-amino ester) nanoparticles for sustained drug delivery across the blood–brain barrier. *Biomacromolecules* **2012**, *13*, 3533-3541.
5. Plesselova, S.; Garcia-Cerezo, P.; Blanco, V.; Reche-Perez, F.J.; Hernandez-Mateo, F.; Santoyo-Gonzalez, F.; Giron-Gonzalez, M.D.; Salto-Gonzalez, R. Polyethylenimine–bisphosphonate–cyclodextrin ternary conjugates: supramolecular systems for the delivery of antineoplastic drugs. *J. Med. Chem.* **2021**, *64*, 12245-12260.
6. Ahmadi, D.; Zarei, M.; Rahimi, M.; Khazaie, M.; Asemi, Z.; Mir, S.M.; Sadeghpour, A.; Karimian, A.; Alemi, F.; Rahmati-Yamchi, M. Preparation and in-vitro evaluation of pH-responsive cationic cyclodextrin coated magnetic nanoparticles for delivery of methotrexate to the Saos-2 bone cancer cells. *J Drug Deliv Sci Technol* **2020**, *57*, 101584-101593.
7. Khoshgard, K.; Ahmadi, N.; Jaymand, M. Stimuli-responsive "theranostic" nanocomposite hydrogels based on β-cyclodextrin containing Fe_3O_4 and Bi_2O_3 nanoparticles for targeted delivery of methotrexate. *Carbohydr. Polym. Technol. Appl. C* **2023**, *6*, 100369-100382.
8. Varan, G.; Benito, J.M.; Mellet, C.O.; Bilensoy, E. Development of polycationic amphiphilic cyclodextrin nanoparticles for anticancer drug delivery. *Beilstein J. Nanotechnol.* **2017**, *8*, 1457-1468.
9. Gil, E.S.; Li, J.; Xiao, H.; Lowe, T.L. Quaternary ammonium β-cyclodextrin nanoparticles for enhancing doxorubicin permeability across the in vitro blood−brain barrier. *Biomacromolecules* **2009**, *10*, 505-516.
10. Belbekhouche, S.; Oniszczuk, J.; Pawlak, A.; El Joukhar, I.; Goffin, A.; Varrault, G.; Carbonnier, B. Cationic poly (cyclodextrin)/alginate nanocapsules: From design to application as efficient delivery vehicle of 4-hydroxy tamoxifen to podocyte in vitro. *Colloids Surf B* **2019**, *179*, 128-135.
11. Lakkakula, J.R.; Matshaya, T.; Krause, R.W.M. Cationic cyclodextrin/alginate chitosan nanoflowers as 5-fluorouracil drug delivery system. *Mater. Sci. Eng. C.* **2017**, *70*, 169-177.

12. Ünal, S.; Aktaş, Y.; Benito, J.M.; Bilensoy, E. Cyclodextrin nanoparticle bound oral camptothecin for colorectal cancer: formulation development and optimization. *Int. J. Pharm.* **2020**, *584*, 119468-119481.
13. Mohammed-Saeid, W.; Karoyo, A.H.; Verrall, R.E.; Wilson, L.D.; Badea, I. Inclusion complexes of melphalan with gemini-conjugated β-cyclodextrin: Physicochemical properties and chemotherapeutic efficacy in in-vitro tumor models. *Pharmaceutics* **2019**, *11*, 427-442.
14. Liao, R.; Liu, Y.; Lv, P.; Wu, D.; Xu, M.; Zheng, X. Cyclodextrin pendant polymer as an efficient drug carrier for scutellarin. *Drug Deliv.* **2020**, *27*, 1741-1749.
15. Liao, R.; Zhao, Y.; Liao, X.; Liu, M.; Gao, C.; Yang, J.; Yang, B. Folic acid-polyamine-β-cyclodextrin for targeted delivery of scutellarin to cancer cells. *Polymer. Adv. Tech.* **2015**, *26*, 487-494.
16. Anirudhan, T.; Dilu, D.; Sandeep, S. Synthesis and characterisation of chitosan crosslinked-β-cyclodextrin grafted silylated magnetic nanoparticles for controlled release of Indomethacin. *J. Magn. Magn. Mater.* **2013**, *343*, 149-156.
17. Norouzi, Z.; Abdouss, M. Electrospun nanofibers using β-cyclodextrin grafted chitosan macromolecules loaded with indomethacin as an innovative drug delivery system. *Int. J. Biol. Macromol.* **2023**, *233*, 123518-123531.
18. Xin, J.; Guo, Z.; Chen, X.; Jiang, W.; Li, J.; Li, M. Study of branched cationic β-cyclodextrin polymer/indomethacin complex and its release profile from alginate hydrogel. *Int. J. Pharm.* **2010**, *386*, 221-228.
19. Piras, A.M.; Zambito, Y.; Burgalassi, S.; Monti, D.; Tampucci, S.; Terreni, E.; Fabiano, A.; Balzano, F.; Uccello-Barretta, G.; Chetoni, P. A water-soluble, mucoadhesive quaternary ammonium chitosan-methyl-β-cyclodextrin conjugate forming inclusion complexes with dexamethasone. *J. Mater. Sci.* **2018**, *29*, 1-13.
20. Choi, J.H.; Park, A.; Lee, W.; Youn, J.; Rim, M.A.; Kim, W.; Kim, N.; Song, J.E.; Khang, G. Preparation and characterization of an injectable dexamethasone-cyclodextrin complexes-loaded gellan gum hydrogel for cartilage tissue engineering. *J. Control. Release.* **2020**, *327*, 747-765.
21. Cheng, H.; Fan, X.; Wu, C.; Wang, X.; Wang, L.J.; Loh, X.J.; Li, Z.; Wu, Y.L. Cyclodextrin-based star-like amphiphilic cationic polymer as a potential pharmaceutical carrier in macrophages. *Macromol. Rapid Commun.* **2019**, *40*, 1800207-1800214.
22. Li, J.; Xiao, H.; Li, J.; Zhong, Y. Drug carrier systems based on water-soluble cationic β-cyclodextrin polymers. *Int. J. Pharm.* **2004**, *278*, 329-342.
23. Yuan, Z.; Ye, Y.; Gao, F.; Yuan, H.; Lan, M.; Lou, K.; Wang, W. Chitosan-graft-β-cyclodextrin nanoparticles as a carrier for controlled drug release. *Int. J. Pharm.* **2013**, *446*, 191-198.
24. Prabaharan, M.; Jayakumar, R. Chitosan-graft-β-cyclodextrin scaffolds with controlled drug release capability for tissue engineering applications. *Int. J. Biol. Macromol* **2009**, *44*, 320-325.
25. Cordaro, A.; Zagami, R.; Malanga, M.; Venkatesan, J.K.; Alvarez-Lorenzo, C.; Cucchiarini, M.; Piperno, A.; Mazzaglia, A. Cyclodextrin cationic polymer-based nanoassemblies to manage inflammation by intra-articular delivery strategies. *Nanomaterials* **2020**, *10*, 1712-1731.
26. Volkova, T.; Surov, A.; Terekhova, I. Metal–organic frameworks based on β-cyclodextrin: design and selective entrapment of non-steroidal anti-inflammatory drugs. *J. Mater. Sci.* **2020**, *55*, 13193-13205.
27. Delyagina, E.; Agafonov, M.; Garibyan, A.; Terekhova, I. γ-Cyclodextrin Based Metal–Organic Framework As a Niflumic Acid Delivery System. *Russ. J. Phys. Chem. A* **2022**, *96*, 1687-1692.
28. Szente, L.; Puskás, I.; Csabai, K.; Fenyvesi, É. Supramolecular proteoglycan aggregate mimics: cyclodextrin-assisted biodegradable polymer assemblies for electrostatic-driven drug delivery. *Chem. Asian J.* **2014**, *9*, 1365-1372.
29. Jafar, M.; Salahuddin, M.; Kayed, T.S.; Ahmad, N.; Al-Eid, H.A.; Al-Qarros, A.H. Buoyant in situ gels of meloxicam-β-cyclodextrintriethanolamine ternary complex for oral delivery; from a box-behnken experimental design to in vivo activity detail. *Asian J Chem* **2017**, *29*, 1275-1284.

30. Pattanaik, S.; Nandi, S.; Sahoo, R.N.; Nanda, A.; Swain, R.; Das, S.; Mallick, S. Budesonide-cyclodextrin in hydrogel system: impact of quaternary surfactant on in vitro-in vivo assessment of mucosal drug delivery. *Rev Chim* **2020**, *71*, 332-345.
31. Dhiman, P.; Bhatia, M. Microwave assisted quaternized cyclodextrin grafted chitosan (QCD-g-CH) nanoparticles entrapping ciprofloxacin. *J. Polym. Res.* **2021**, *28*, 1-14.
32. Keshavarz, A.H.; Montazer, M.; Soleimani, N. In situ synthesis of polyamidoamine/β-cyclodextrin/silver nanocomposites on polyester fabric tailoring drug delivery and antimicrobial properties. *React. Funct. Polym* **2020**, *152*, 104602-104614.
33. Salih, M.; Omolo, C.A.; Agrawal, N.; Walvekar, P.; Waddad, A.Y.; Mocktar, C.; Ramdhin, C.; Govender, T. Supramolecular amphiphiles of beta-cyclodextrin and oleylamine for enhancement of vancomycin delivery. *Int. J. Pharm.* **2020**, *574*, 118881-118898.
34. Evangelista, T.F.; Andrade, G.R.; Nascimento, K.N.; Dos Santos, S.B.; Santos, M.d.F.C.; D'Oca, C.D.R.M.; Estevam, C.d.S.; Gimenez, I.F.; Almeida, L.E. Supramolecular polyelectrolyte complexes based on cyclodextrin-grafted chitosan and carrageenan for controlled drug release. *Carbohydr. Polym.* **2020**, *245*, 116592-116603.
35. Agnes, M.; Thanassoulas, A.; Stavropoulos, P.; Nounesis, G.; Miliotis, G.; Miriagou, V.; Athanasiou, E.; Benkovics, G.; Malanga, M.; Yannakopoulou, K. Designed positively charged cyclodextrin hosts with enhanced binding of penicillins as carriers for the delivery of antibiotics: the case of oxacillin. *Int. J. Pharm.* **2017**, *531*, 480-491.
36. Thomsen, H.; Agnes, M.; Uwangue, O.; Persson, L.; Mattsson, M.; Graf, F.E.; Kasimati, E.-M.; Yannakopoulou, K.; Ericson, M.B.; Farewell, A. Increased antibiotic efficacy and noninvasive monitoring of *Staphylococcus epidermidis* biofilms using per-cysteamine-substituted γ-cyclodextrin–A delivery effect validated by fluorescence microscopy. *Int. J. Pharm.* **2020**, *587*, 119646-119655.
37. Wei, Y.; Chen, C.; Zhai, S.; Tan, M.; Zhao, J.; Zhu, X.; Wang, L.; Liu, Q.; Dai, T. Enrofloxacin/florfenicol loaded cyclodextrin metal-organic-framework for drug delivery and controlled release. *Drug Deliv.* **2021**, *28*, 372-379.
38. Ding, W.Y.; Zheng, S.D.; Qin, Y.; Yu, F.; Bai, J.-W.; Cui, W.-Q.; Yu, T.; Chen, X.-R.; Bello-Onaghise, G.s.; Li, Y.-H. Chitosan grafted with β-cyclodextrin: synthesis, characterization, antimicrobial activity, and role as absorbefacient and solubilizer. *Front. Chem.* **2019**, *6*, 657-661.
39. Le-Deygen, I.M.; Skuredina, A.A.; Mamaeva, P.V.; Kolmogorov, I.M.; Kudryashova, E.V. Conjugates of chitosan with β-Cyclodextrins as promising carriers for the delivery of levofloxacin: spectral and microbiological studies. *Life* **2023**, *13*, 272-287.
40. Hada, A.-M.; Potara, M.; Astilean, S.; Cordaro, A.; Neri, G.; Malanga, M.; Nostro, A.; Mazzaglia, A.; Scala, A.; Piperno, A. Linezolid nanoAntiobiotics and SERS-nanoTags based on polymeric cyclodextrin bimetallic core-shell nanoarchitectures. *Carbohydr. Polym.* **2022**, *293*, 119736-119745.
41. Gómez-Galván, F.; Pérez-Álvarez, L.; Matas, J.; Álvarez-Bautista, A.; Poejo, J.; Duarte, C.M.; Ruiz-Rubio, L.; Vila-Vilela, J.L.; León, L.M. Preparation and characterization of soluble branched ionic β-cyclodextrins and their inclusion complexes with triclosan. *Carbohydr. Polym.* **2016**, *142*, 149-157.
42. Qian, L.; Guan, Y.; Ziaee, Z.; He, B.; Zheng, A.; Xiao, H. Rendering cellulose fibers antimicrobial using cationic β-cyclodextrin-based polymers included with antibiotics. *Cellulose* **2009**, *16*, 309-317.
43. Liu, T.; Li, G.; Wu, X.; Chen, S.; Zhang, S.; Han, H.; Zhang, H.; Luo, X.; Cai, X.; Ma, D. β-cyclodextrin-graft-poly (amidoamine) dendrons as the nitric oxide deliver system for the chronic rhinosinusitis therapy. *Drug Deliv.* **2021**, *28*, 306-318.
44. Qian, L.; Guan, Y.; Xiao, H. Preparation and characterization of inclusion complexes of a cationic β-cyclodextrin polymer with butylparaben or triclosan. *Int. J. Pharm.* **2008**, *357*, 244-251.
45. Teng, J.; Chen, S.; Zhang, J.; Yang, J.; Pang, R.; Zhi, X.; Yang, T.; Zhao, Y. pH-responsive nanoparticles based on sodium dodecylbenzene sulfonate and polyamine-modified cyclodextrins for controlled release of metformin hydrochloride. *Iran. Polym. J.* **2022**, *31*, 1069-1078.

46. Presas, E.; Tovar, S.; Cuñarro, J.; O'Shea, J.P.; O'Driscoll, C.M. Pre-clinical evaluation of a modified cyclodextrin-based nanoparticle for intestinal delivery of liraglutide. *J. Pharm. Sci.* **2021**, *110*, 292-300.
47. Zhang, H.; Huang, X.; Sun, Y.; Lu, G.; Wang, K.; Wang, Z.; Xing, J.; Gao, Y. Improvement of pulmonary absorption of poorly absorbable macromolecules by hydroxypropyl-β-cyclodextrin grafted polyethylenimine (HP-β-CD-PEI) in rats. *Int. J. Pharm.* **2015**, *489*, 294-303.
48. Wang, L.; Yang, Y.-W.; Zhu, M.; Qiu, G.; Wu, G.; Gao, H. β-Cyclodextrin-conjugated amino poly (glycerol methacrylate) s for efficient insulin delivery. *RSC Advances* **2014**, *4*, 6478-6485.
49. Zhang, N.; Li, J.; Jiang, W.; Ren, C.; Li, J.; Xin, J.; Li, K. Effective protection and controlled release of insulin by cationic β-cyclodextrin polymers from alginate/chitosan nanoparticles. *Int. J. Pharm.* **2010**, *393*, 213-219.
50. Heydari, A.; Doostan, F.; Khoshnood, H.; Sheibani, H. Water-soluble cationic poly (β-cyclodextrin-co-guanidine) as a controlled vitamin B_2 delivery carrier. *RSC Adv.* **2016**, *6*, 33267-33278.
51. Soltani, Y.; Goodarzi, N.; Mahjub, R. Preparation and characterization of self nano-emulsifying drug delivery system (SNEDDS) for oral delivery of heparin using hydrophobic complexation by cationic polymer of β-cyclodextrin. *Drug Dev. Ind. Pharm*. **2017**, *43*, 1899-1907.
52. Otake, H.; Kobayashi, K.; Kadowaki, R.; Kosaka, T.; Itahashi, M.; Tsubaki, M.; Matsuda, M.; Iwakiri, N.; Harata, E.; Nagai, N. Copolymerized polymers based on cyclodextrins and cationic groups enhance therapeutic effect of rebamipide in the N-acetylcysteine-treated dry eye model. *Drug Des Devel Ther* **2024**, 4345-4358.
53. Kamiński, K.; Kujdowicz, M.; Kajta, M.; Nowakowska, M.; Szczubiałka, K. Enhanced delivery of daidzein into fibroblasts and neuronal cells with cationic derivatives of gamma-cyclodextrin for the control of cellular glycosaminoglycans. *Eur J Pharm Biopharm* **2015**, *91*, 111-119.
54. Yang, C.; Li, H.; Goh, S.H.; Li, J. Cationic star polymers consisting of α-cyclodextrin core and oligoethylenimine arms as nonviral gene delivery vectors. *Biomaterials* **2007**, *28*, 3245-3254.
55. Li, J.; Guo, Z.; Xin, J.; Zhao, G.; Xiao, H. 21-Arm star polymers with different cationic groups based on cyclodextrin core for DNA delivery. *Carbohydr. Polym.* **2010**, *79*, 277-283.
56. Zhang, Y.; Chen, Y.; Zhang, Y.; Yang, Y.; Chen, J.; Liu, Y. Recycling Gene carrier with high efficiency and low toxicity mediated by L-cystine-bridged bis (β-cyclodextrin). *Sci. Rep.* **2015**, *4*, 7471-7477.
57. Lv, P.; Zhou, C.; Zhao, Y.; Liao, X.; Yang, B. Modified-epsilon-polylysine-grafted-PEI-β-cyclodextrin supramolecular carrier for gene delivery. *Carbohydr. Polym.* **2017**, *168*, 103-111.
58. Aranda, C.; Urbiola, K.; Ardoy, A.M.; Fernández, J.M.G.; Mellet, C.O.; de Ilarduya, C.T. Targeted gene delivery by new folate–polycationic amphiphilic cyclodextrin–DNA nanocomplexes in vitro and in vivo. *Eur. J. Pharm. Biopharm.* **2013**, *85*, 390-397.
59. Zhao, F.; Yin, H.; Zhang, Z.; Li, J. Folic acid modified cationic γ-cyclodextrin-oligoethylenimine star polymer with bioreducible disulfide linker for efficient targeted gene delivery. *Biomacromolecules* **2013**, *14*, 476-484.
60. Huang, H.; Yu, H.; Tang, G.; Wang, Q.; Li, J. Low molecular weight polyethylenimine cross-linked by 2-hydroxypropyl-γ-cyclodextrin coupled to peptide targeting HER2 as a gene delivery vector. *Biomaterials* **2010**, *31*, 1830-1838.
61. Ooya, T.; Choi, H.S.; Yamashita, A.; Yui, N.; Sugaya, Y.; Kano, A.; Maruyama, A.; Akita, H.; Ito, R.; Kogure, K. Biocleavable polyrotaxane−plasmid DNA polyplex for enhanced gene delivery. *J. Am. Chem. Soc.* **2006**, *128*, 3852-3853.
62. Li, H.; Peng, E.; Zhao, F.; Li, J.; Xue, J. Supramolecular surface functionalization of iron oxide nanoparticles with α-cyclodextrin-based cationic star polymer for magnetically-enhanced gene delivery. *Pharmaceutics* **2021**, *13*, 1884-1899.

63. Zeng, Y.; Zhou, Z.; Fan, M.; Gong, T.; Zhang, Z.; Sun, X. PEGylated cationic vectors containing a protease-sensitive peptide as a miRNA delivery system for treating breast cancer. *Mol. Pharmaceutics.* **2017**, *14*, 81-92.
64. Li, F.; Cao, D.; Gu, W.; Cui, L.; Qiu, Z.; Liu, Z.; Li, D.; Guo, X. Delivery of miR-34a-5p by folic acid-modified β-cyclodextrin-grafted polyethylenimine copolymer nanocarriers to resist KSHV. *ACS Appl. Nano Mater.* **2023**, *6*, 10826-10836.
65. Fitzgerald, K.A.; Malhotra, M.; Gooding, M.; Sallas, F.; Evans, J.C.; Darcy, R.; O'Driscoll, C.M. A novel, anisamide-targeted cyclodextrin nanoformulation for siRNA delivery to prostate cancer cells expressing the sigma-1 receptor. *Int. J. Pharm.* **2016**, *499*, 131-145.
66. Guo, J.; Ogier, J.R.; Desgranges, S.; Darcy, R.; Caitriona, O. Anisamide-targeted cyclodextrin nanoparticles for siRNA delivery to prostate tumours in mice. Biomaterials **2012**, 33, 7775-7784.
67. Li, J.M.; Wang, Y.Y.; Zhang, W.; Su, H.; Ji, L.N.; Mao, Z.W. Low-weight polyethylenimine cross-linked 2-hydroxypopyl-β-cyclodextrin and folic acid as an efficient and nontoxic siRNA carrier for gene silencing and tumor inhibition by VEGF siRNA. *Int J Nanomedicine* **2013**, 2101-2117.
68. Li, N.; Luo, H.C.; Yang, C.; Deng, J.J.; Ren, M.; Xie, X.Y.; Lin, D.-Z.; Yan, L.; Zhang, L.-M. Cationic star-shaped polymer as an siRNA carrier for reducing MMP-9 expression in skin fibroblast cells and promoting wound healing in diabetic rats. *Int J Nanomedicine* **2014**, 3377-3387.
69. Kulkarni, A.; DeFrees, K.; Schuldt, R.A.; Hyun, S.-H.; Wright, K.J.; Yerneni, C.K.; VerHeul, R.; Thompson, D.H. Cationic α-cyclodextrin: poly (ethylene glycol) polyrotaxanes for siRNA delivery. *Mol. Pharmaceutics.* **2013**, *10*, 1299-1305.
70. Dandekar, P.; Jain, R.; Keil, M.; Loretz, B.; Muijs, L.; Schneider, M.; Auerbach, D.; Jung, G.; Lehr, C.-M.; Wenz, G. Cellular delivery of polynucleotides by cationic cyclodextrin polyrotaxanes. *J. Control. Release.* **2012**, *164*, 387-393.
71. Wan, N.; Huan, M.-L.; Ma, X.-X.; Jing, Z.-W.; Zhang, Y.-X.; Li, C.; Zhou, S.-Y.; Zhang, B.-L. Design and application of cationic amphiphilic β-cyclodextrin derivatives as gene delivery vectors. *Nanotechnology* **2017**, *28*, 465101-465111.
72. Fan, X.; Cheng, H.; Wu, Y.; Loh, X.J.; Wu, Y.L.; Li, Z. Incorporation of polycaprolactone to cyclodextrin-based nanocarrier for potent gene delivery. *Macromol. Mater. Eng.* **2018**, *303*, 1800255-1800263.
73. Liu, C.-H.; Shih, P.-Y.; Lin, C.-H.; Chen, Y.-J.; Wu, W.-C.; Wang, C.-C. Tetraethylenepentamine-coated β cyclodextrin nanoparticles for dual DNA and siRNA delivery. *Pharmaceutics* **2022**, *14*, 921-941.
74. Khazaei Monfared, Y.; Mahmoudian, M.; Zakeri-Milani, P.; Cecone, C.; Hayashi, T.; Ishii, K.J.; Conde, J.; Matencio, A.; Trotta, F. Intratumoural delivery of mRNA loaded on a cationic hyper-branched cyclodextrin-based polymer induced an anti-tumour immunological response in melanoma. *Cancers* **2023**, *15*, 3748-3767.
75. Seripracharat, C.; Sinthuvanich, C.; Karpkird, T. Cationic cyclodextrin-adamantane poly (vinyl alcohol)-poly (ethylene glycol) assembly for siRNA delivery. *J Drug Deliv Sci Technol* **2022**, *68*, 103052-103062.
76. Ping, Y.; Liu, C.; Zhang, Z.; Liu, K.L.; Chen, J.; Li, J. Chitosan-graft-(PEI-β-cyclodextrin) copolymers and their supramolecular PEGylation for DNA and siRNA delivery. *Biomaterials* **2011**, *32*, 8328-8341.
77. Xiong, Q.; Cui, M.; Bai, Y.; Liu, Y.; Liu, D.; Song, T. A supramolecular nanoparticle system based on β-cyclodextrin-conjugated poly-l-lysine and hyaluronic acid for co-delivery of gene and chemotherapy agent targeting hepatocellular carcinoma. *Colloids Surf B Biointerfaces* **2017**, *155*, 93-103.
78. Mousazadeh, H.; Khorsandi, M.; Zarghami, N. Stimulus-responsive nanocarrier from star-shaped polyethyleneimine-β-cyclodextrin coated mesoporous silica for targeted combination cancer therapy. *J Drug Deliv Sci Technol* **2023**, *88*, 104940-104952.
79. Zhou, X.; Xu, L.; Xu, J.; Wu, J.; Kirk, T.B.; Ma, D.; Xue, W. Construction of a high-efficiency drug and gene co-delivery system for cancer therapy from a pH-sensitive supramolecular inclusion between oligoethylenimine-graft-β-cyclodextrin and hyperbranched polyglycerol derivative. *ACS Appl. Mater. Interfaces* **2018**, *10*, 35812-35829.

80. Liu, T.; Xue, W.; Ke, B.; Xie, M.-Q.; Ma, D. Star-shaped cyclodextrin-poly (l-lysine) derivative co-delivering docetaxel and MMP-9 siRNA plasmid in cancer therapy. *Biomaterials* **2014**, *35*, 3865-3872.
81. Deng, J.; Li, N.; Mai, K.; Yang, C.; Yan, L.; Zhang, L.-M. Star-shaped polymers consisting of a β-cyclodextrin core and poly (amidoamine) dendron arms: binding and release studies with methotrexate and siRNA. *J. Mater. Chem.* **2011**, *21*, 5273-5281.
